# Impact of the TCR Signal on Regulatory T Cell Homeostasis, Function, and Trafficking

**DOI:** 10.1371/journal.pone.0006580

**Published:** 2009-08-11

**Authors:** Joong Kyu Kim, Mark Klinger, Jonathan Benjamin, Yuanyuan Xiao, David J. Erle, Dan R. Littman, Nigel Killeen

**Affiliations:** 1 Department of Microbiology and Immunology, University of California San Francisco, San Francisco, California, United States of America; 2 Howard Hughes Medical Institute and Kimmel Center for Biology and Medicine of the Skirball Institute, Departments of Microbiology and Pathology, New York University School of Medicine, New York, New York, United States of America; 3 Lung Biology Center, Department of Medicine, University of California San Francisco, San Francisco, California, United States of America; New York University School of Medicine, United States of America

## Abstract

Signaling through the T cell antigen receptor (TCR) is important for the homeostasis of naïve and memory CD4^+^ T cells. The significance of TCR signaling in regulatory T (Treg) cells has not been systematically addressed. Using an *Ox40-cre* allele that is prominently expressed in Treg cells, and a conditional null allele of the gene encoding p56*^Lck^*, we have examined the importance of TCR signaling in Treg cells. Inactivation of p56*^Lck^* resulted in abnormal Treg homeostasis characterized by impaired turnover, preferential redistribution to the lymph nodes, loss of suppressive function, and striking changes in gene expression. Abnormal Treg cell homeostasis and function did not reflect the involvement of p56*^Lck^* in CD4 function because these effects were not observed when CD4 expression was inactivated by *Ox40-cre*.The results make clear multiple aspects of Treg cell homeostasis and phenotype that are dependent on a sustained capacity to signal through the TCR.

## Introduction

Regulatory T cells (Treg cells) are defined by expression of the Forkhead transcription factor FoxP3 and by potent immunosuppressive capability [1]–[4]. These cells develop in the thymus through a CD25^hi^CD4^+^CD8^−^ intermediate in a process that depends on the γc cytokines IL-2, -7 or -15 [5]–[7]. Treg cells can also be generated extrathymically in a TGFβ-dependent fashion from conventional naïve T cells [8]–[14] and such induced Treg cells can be of significance for control of destructive immune responses [15]. The crucial importance of Treg cells for preventing autoimmunity is made clear by the systemic disease that develops when they are defective or ablated [2], [3], [16], [17].

T cells show variable dependency on TCR signaling for homeostasis as a function of their lineage and differentiation state. Naïve T cells are dependent on tonic TCR signaling for survival, but they lose this dependency when they become memory cells [18]–[21]. There have, however, been conflicting observations about this [22], [23]. While it has not been systematically addressed, there are indications that like memory CD8^+^ T cells, Treg cell homeostasis is minimally influenced by TCR signaling [24].

In this paper we have examined the importance of TCR signaling in Treg cell homeostasis using a novel mouse strain that features conditional loss of TCR signaling due to inactivation of p56*^Lck^* function. We find that the deficiency substantially changes the gene expression profile and the turnover of Treg cells, while also causing them to redistribute from the spleen and the tissues to the lymph nodes. The results reveal important roles for TCR signaling in maintaining the Treg phenotype and in governing Treg cell migration.

## Results

### Expression of Ox40-cre in Regulatory T Cells

**Figure 1:**
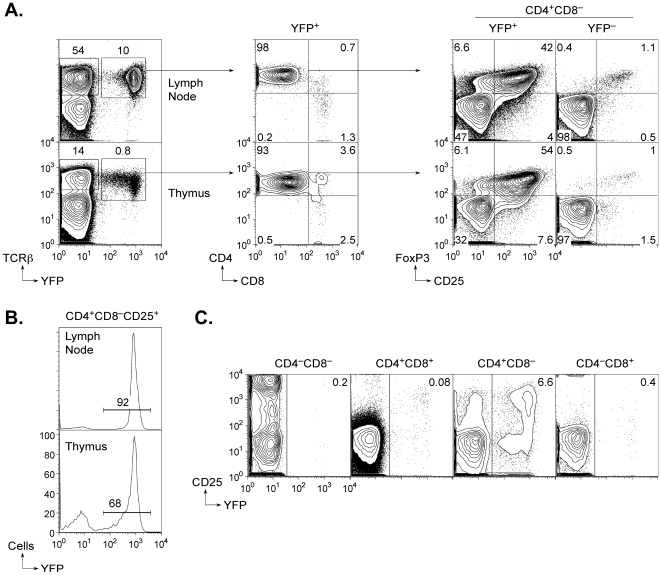
Activity of *Ox40-cre* in regulatory T cells. A. Thymic and lymph node expression of YFP in *Ox40-cre*/YFP mice (i.e., *Ox40-cre* mice carrying a *ROSA26-loxP-STOP-loxP-YFP* allele). The contour plots show that YFP expression is predominantly found on CD4^+^CD8^−^ cells in both tissues (as determined by flow cytometry). FoxP3^+^ cells are enriched 30–40 fold in the YFP^+^CD4^+^CD8^−^population compared to YFP^−^ cells. B. Penetrance of *Ox40-cre* recombination in CD4^+^CD8^−^CD25^+^ cells in the lymph nodes and thymus. The histograms show the frequency of YFP expression in regulatory phenotype cells in both tissues of *Ox40-cre*/YFP mice. C. Developmental stage-specific activity of *Ox40-cre* in the thymus. The plots show YFP expression is predominantly found in cells that are CD4^+^CD8^−^ and is barely detectable at the CD4^+^CD8^+^ stage.

*Ox40-cre* is an allele of the *Ox40* gene that expresses the Cre recombinase in place of OX40 [25]. To determine which cells undergo Cre recombination in *Ox40-cre* mice, we crossed them to mice carrying a Cre-activated *ROSA26-YFP* reporter allele [26]. 98% of peripheral T cells that had undergone recombination and become YFP^+^ in such mice were CD4^+^ (Figure 1A), and among these one-third to one-half were FoxP3^+^ depending on age at the time of analysis. Strikingly, approximately 90% of lymph node or spleen CD25^+^ T cells were YFP^+^ indicating that *Ox40-cre* is highly efficient at inducing recombination in the regulatory lineage (Figure 1B).

Less than 1% of thymocytes in *Ox40-cre*/YFP mice had undergone recombination, and the majority of these were CD4^+^CD8^−^ single-positive cells (Figure 1A). As in the periphery, there was a high frequency of FoxP3^+^ cells in the affected population. Thymic precursors of Treg cells can be discriminated by a CD25^hi^ CD4^+^CD8^−^ phenotype [6], and 65–70% of these were YFP^+^. Double-negative and double-positive thymocytes were negative for YFP expression (Figure 1C). Thus, *Ox40-cre* activity was evident in the thymus at the stage at which cells induce FoxP3 expression and are committed to the Treg lineage.

### Disruption of TCR signaling in Regulatory T cells by Ox40-cre

To examine the importance of TCR signaling in the homeostasis and function of Treg cells, we crossed *Ox40-cre* mice to mice carrying a conditional null (*loxP*-modified) allele of the *Lck* gene (Benjamin *et al.*, in preparation). Cre recombination incapacitates this allele such that it can no longer express a functional p56*^Lck^* protein. Although loss of p56*^Lck^* greatly impairs TCR signaling [27], the related *Src*-family kinase p59*^Fyn^* can partially compensate for its absence in some settings, most notably at the pre-TCR-dependent stage of development in the thymus [28], [29]. For this reason, we combined the *Ox40-cre* and conditional null alleles of *Lck* (*Lck* ^c^) with a null allele of the *Fyn* gene so that we could deprive cells of both kinases when they expressed the *Ox40* gene.

Mice with four types of T cell genotypes were examined in our experiments: wild-type (Control), Fyn-deficient (*Fyn* ^−/−^ referred to as Fyn), Lck-deficient (*Lck* ^c/−^;*Ox40-cre* referred to as Lck), and Lck,Fyn-deficient (*Fyn* ^−/−^;*Lck* ^c/−^;*Ox40-cre* referred to as Lck/Fyn). The *Ox40-cre* allele was always kept heterozygous so that T cells would retain the capacity to express near-normal levels of cell surface OX40 from one copy of the wild-type *Ox40* allele [30]. *Ox40-cre* heterozygous mice (lacking conditional null alleles) were free of detectable abnormal T cell phenotypes (data not shown).

**Figure 2:**
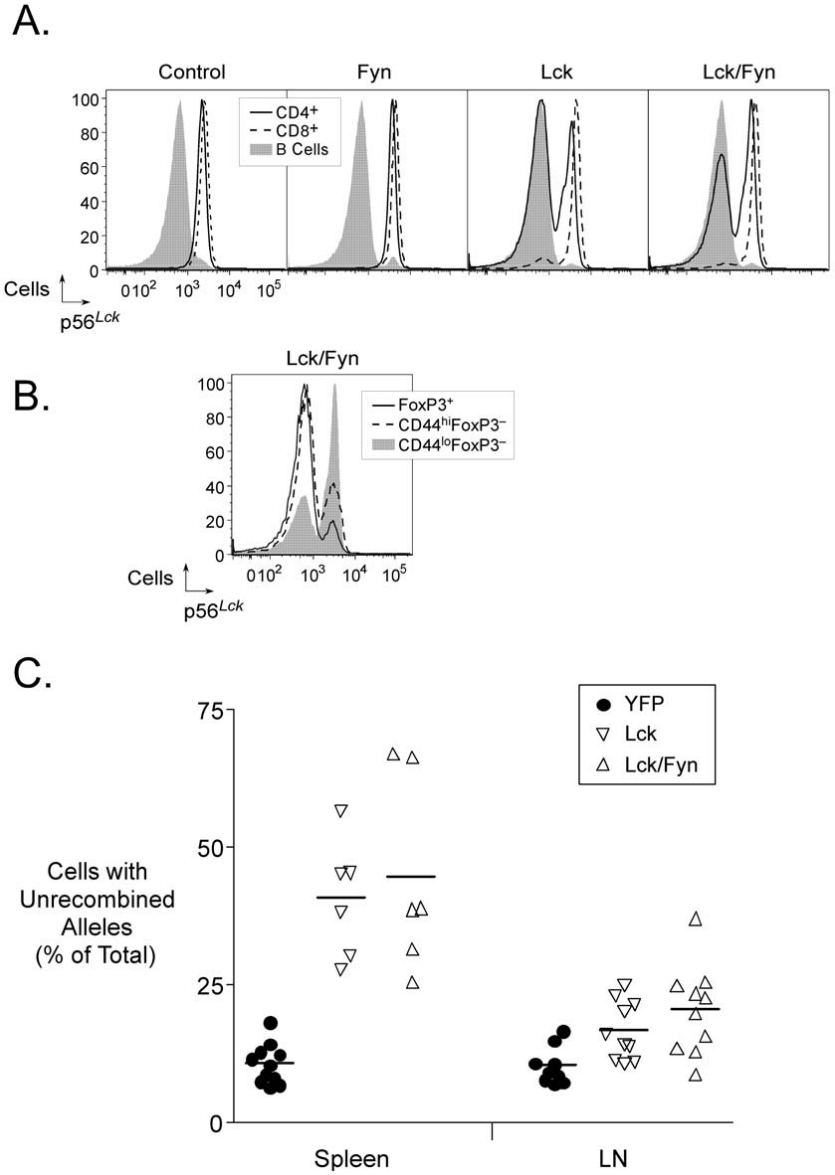
*Ox40-cre*-mediated inactivation of p56*^Lck^* in T cells. A. Selective inactivation of p56*^Lck^* in CD4^+^ T cells in lymph nodes of *Ox40-cre* mice carrying a conditional null allele of the *Lck* gene. The histograms show intracellular p56*^Lck^* detected with the 1F6 monoclonal antibody in B cells, CD4^+^ and CD8^+^ T cells in mice of the indicated genotypes. B. Intracellular *p56^Lck^* (as in A.) in T cells of the indicated phenotypes (regulatory, memory and naïve) from Lck/Fyn mice. C. Increased frequency of Treg cells that had not undergone *Ox40-cre*-mediated recombination in mice carrying the conditional null *Lck* allele compared to mice carrying the *ROSA26-YFP* allele. The graph shows the frequencies of YFP^−^ or p56*^Lck^*-positive (1F6^+^) CD25^+^ Treg cells in individual mice of the indicated genotypes.

*Ox40-cre*-dependent loss of p56*^Lck^* expression could be visualized by intracellular flow cytometry using the 1F6 p56*^Lck^*-specific monoclonal antibody (Figure 2). Whereas CD4^+^ T cells from control or Fyn mice showed equivalent staining with this antibody, there was selective loss of immunoreactivity in CD4^+^ T cells from Lck or Lck/Fyn mice (Figure 2A). Inactivation of p56*^Lck^* could be detected in a small fraction of phenotypically naïve CD4^+^ T cells, a larger fraction of memory-phenotype CD4^+^ T cells, and the majority of FoxP3^+^ T cells (Figure 2B). These observations were largely consistent with the expected pattern of expression of *Ox40-cre* based on the analysis of mice carrying the YFP reporter (Figure 1 and data not shown). We noted, however, that the frequency of Treg cells that retained wild-type p56*^Lck^* was two-to-four-fold higher than the YFP analysis predicted in the lymph nodes and spleen respectively (Figure 2C). This disparity raised the possibility that Treg cell homeostasis was disturbed by inactivation of p56*^Lck^*.

### Abnormal T cell homeostasis caused by inactivation of the Lck gene by Ox40-cre

**Figure 3:**
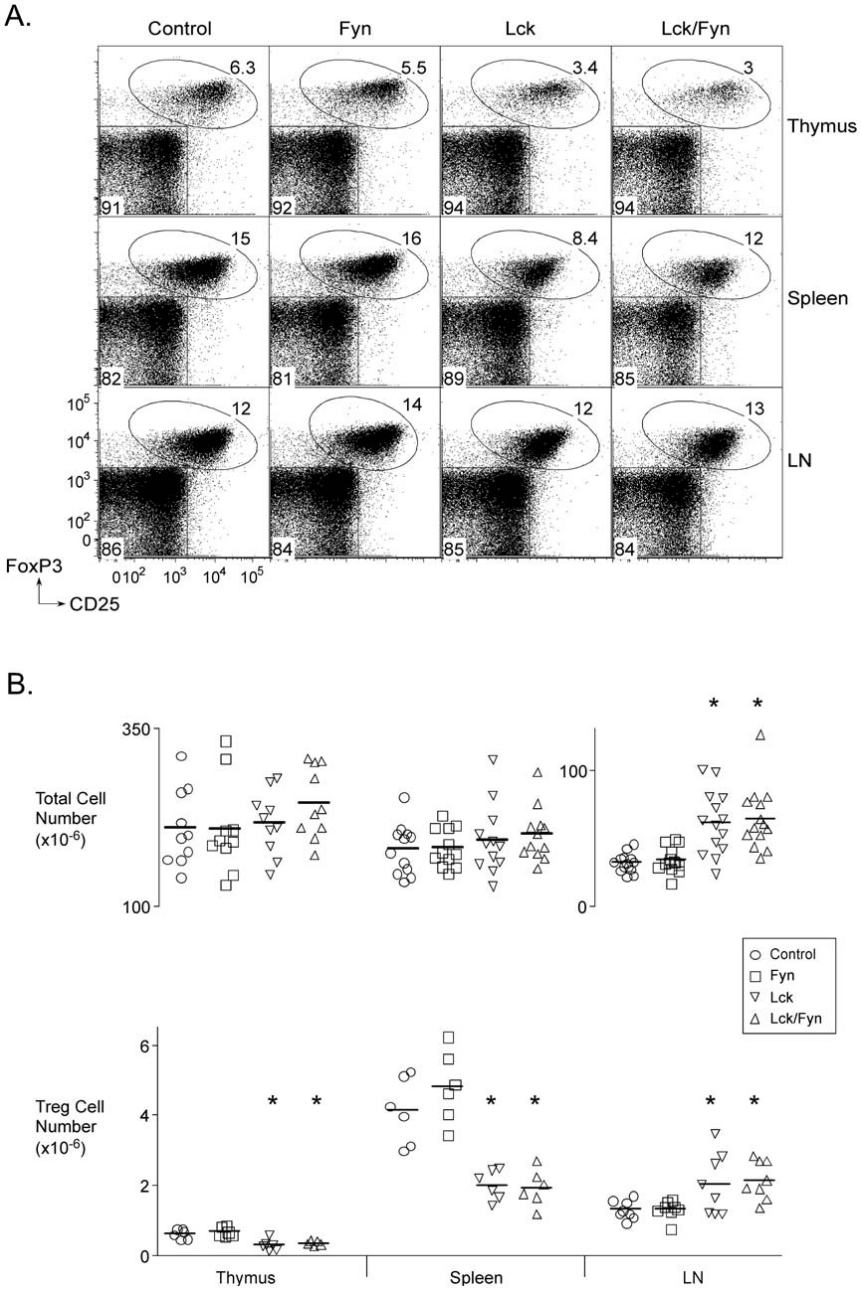
p56*^Lck^*-deficient regulatory T cells. A. Flow cytometry data showing frequencies of Treg cells in mice of the indicated genotypes in the thymus, spleen and lymph nodes. B. Frequencies of thymic and peripheral Treg cells in individual mice of the indicated genotypes (bottom panel). The top panel shows total cell recovery from each of the tissues in the same mice. Asterisks identify differences (compared to control) that were statistically significant by ANOVA (p<0.05).

Regulatory T cells were about two-fold reduced in number in the thymuses and spleens of Lck and Lck/Fyn mice compared to control or Fyn mice (Figure 3AB). By contrast their numbers were increased in the lymph nodes of the two Lck mutants (Figure 3B) but this was associated with little, if any, change in their representation as a fraction of the CD4^+^ population (Figure 3A). Instead, the apparent accumulation of p56*^Lck^*-deficient Treg cells in the lymph nodes was a consequence of mild lymphadenopathy in the mutant mice and an associated increase in T cell numbers (Figure 3B).

p56*^Lck^* deficiency has been found previously to impair proliferation of T cells in lymphopenic recipients [31]. To test whether this was also true of Treg cells and might account for some of the homeostatic defects just described, we purified CD25^+^ and CD25^−^ cells from mice of the four genotypes and transferred them intravenously into T cell-deficient recipients. Wild-type and p59*^Fyn^*-deficient CD25^+^ cells demonstrated characteristic proliferation in the recipient mice, whereas the Lck and Lck/Fyn T cells failed to make this response (Figure S1A). p56*^Lck^*-deficient CD25^−^ cells were capable of some expansion but they failed to go through the same number of divisions as cells that expressed p56*^Lck^*.

To look further at Treg cell homeostasis following loss of p56*^Lck^*, we labeled dividing cells *in vivo* by treating the mice with BrdU for two weeks. We then monitored the decay of BrdU^+^ cells (due to division or death) over time. BrdU labeling was much more prominent in cells that retained p56*^Lck^* than in cells in the same mice (or in control mice) that had lost it (Figure S1B). Moreover, although the control cells showed marked loss of the BrdU label during the analysis period, the frequency of labeled p56*^Lck^*-deficient cells was considerably more stable. Thus, loss of p56*^Lck^* expression impaired Treg cell turnover without imparting a major effect on survival. Consistent with these data, DAPI labeling experiments revealed a much-reduced frequency of mitoses in p56*^Lck^*-deficient compared to p56*^Lck^*-expressing Treg cells (Figure S1C).

### TCR Signaling is Not Required for Retention of FoxP3 Expression, but is Required for Regulatory T cell Function

**Figure 4:**
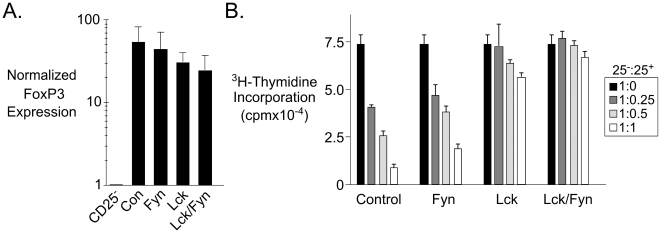
Retention of FoxP3 but abrogation of *in vitro* suppression in *Lck*-mutant regulatory cells. A. Real-time PCR analysis of FoxP3 expression in CD25^+^CD4^+^ T cells from mice of the indicated genotypes. Expression values were calculated relative to HPRT as an internal standard using the ΔΔCt method [76], and with normalization to the expression value obtained for the CD25^−^ sample from control mice. B. The graphs show ^3^H-Thymidine uptake in cultures comprised of CD25^−^ and CD25^+^ T cells at the stated ratios from mice of the indicated genotypes. Lymph node cells from mice of the indicated genotypes were flow-sorted then stimulated with soluble anti-CD3 and anti-CD28 in the presence of irradiated wild-type splenic accessory cells.

Loss of p56*^Lck^* did not abrogate FoxP3 expression as detected by FACS (Figure 3A) or by real-time PCR (Figure 4A) although by both techniques there was a reproducible slight decrease in the mean FoxP3 expression level. Some diminution in FoxP3 levels might be expected because acute activation increases FoxP3 protein expression [2], and such activation would not occur in the mutant Treg cells. The data nonetheless indicated that retention of basal FoxP3 expression is a stable aspect of the Treg lineage and does not depend upon persistent TCR signaling.

To test whether TCR signaling was essential for suppression mediated by Treg cells, we sorted CD25^+^CD4^+^ T cells from the four genotypes and mixed them at varying ratios with CD25^−^CD4^+^ T cells before stimulating them *in vitro* with anti-CD3 in the presence of splenic accessory cells. Whereas wild-type and p59*^Fyn^*-deficient CD25^+^ T cells had equivalent capacity to suppress proliferation, the Lck and Lck/Fyn CD25^+^ T cells were incapable of suppression (Figure 4B). Thus, TCR signaling via p56*^Lck^* is essential for suppression by Treg cells.

The CD4 molecule enhances TCR signaling in part through its capacity to interact with p56*^Lck^* [32]. To determine whether, like p56*^Lck^*, CD4 is also important for Treg cell homeostasis and function, we generated mice in which the *Cd4* gene was inactivated by *Ox40-cre* through use of a conditional null *Cd4* allele [33]. These mice had normal frequencies of Treg cells in their lymphoid organs even though the majority of them lacked CD4 expression (data not shown). Consistent with the involvement of CD4 in T cell activation, its loss correlated with a reduction in the frequency of cells expressing markers of acute activation such as CD69 and CD44 (Figure S2). Despite this, however, the CD4-deficient Treg cells were nonetheless capable of efficiently suppressing T cell responses *in vitro* (data not shown). These data make clear that disturbed Treg cell homeostasis and function caused by loss of p56*^Lck^* reflects the primary involvement of this kinase in the TCR signaling pathway, and by contrast Treg cells show little dependency on CD4 for homeostasis or *in vitro* suppression.

### Changes in regulatory T cell gene expression caused by absence of p56^Lck^

**Figure 5:**
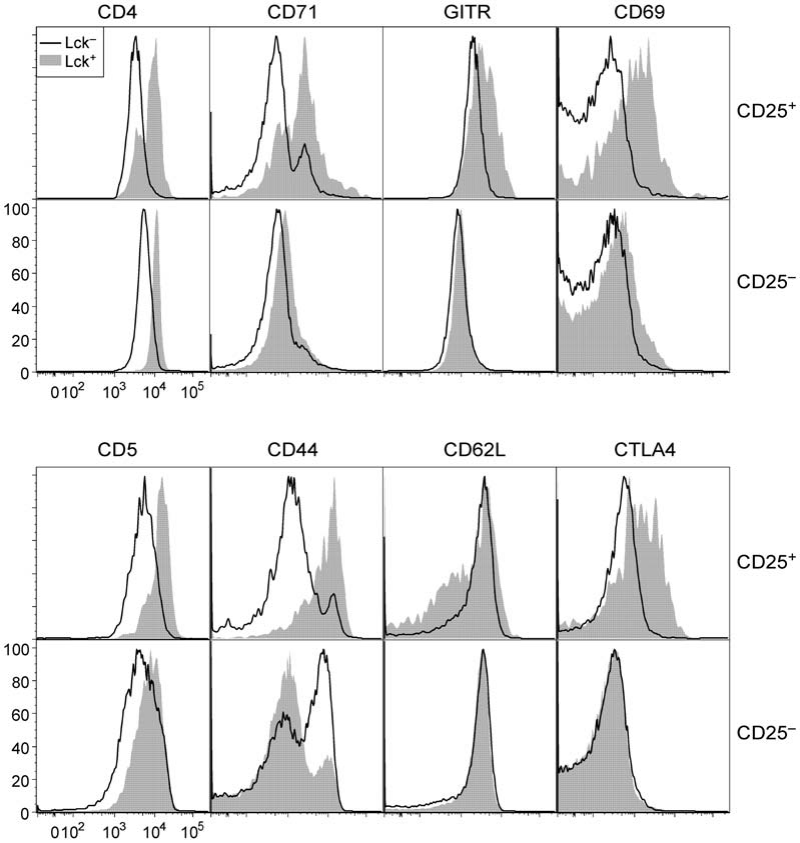
Phenotype of p56*^Lck^*-deficient T cells. Flow cytometry data showing expression of the indicated molecules on CD25^+^ or CD25^−^ lymph node CD4^+^ T cells from Lck/Fyn mice. Cells were permeabilized and stained with the 1F6 antibody to discriminate cells that had undergone *Ox40-cre*-mediated inactivation of the *Lck* gene from those that had not (open versus solid curve respectively).

Although p56*^Lck^*-deficient Treg cells remained positive for FoxP3 and CD25 expression, we considered it likely that the loss of TCR signaling would incur changes in gene expression. This might be predicted if the TCR signal is important for maintaining the differentiated state of the cells, and would also follow from prior data showing that tonic TCR signaling regulates expression of numerous genes in T cells [34]. By flow cytometry, we noted multiple changes in the expression of molecules known to be regulated by TCR signaling such as GITR, CD69, CD44, CD62L and CTLA4 (Figure 5). CD4 expression was also reduced, consistent with the known involvement of p56*^Lck^* in reducing the rate of CD4 endocytosis [35], [36].

To look more exhaustively for changes in gene expression, we used microarrays to compare Treg cells that did or did not express p56*^Lck^*. To identify p56*^Lck^*-dependent changes in gene expression that were unique to Treg cells, we also made the same comparison for CD4^+^CD25^−^CD44^hi^ cells (*i.e.*, memory/effector T cells).

**Figure 6:**
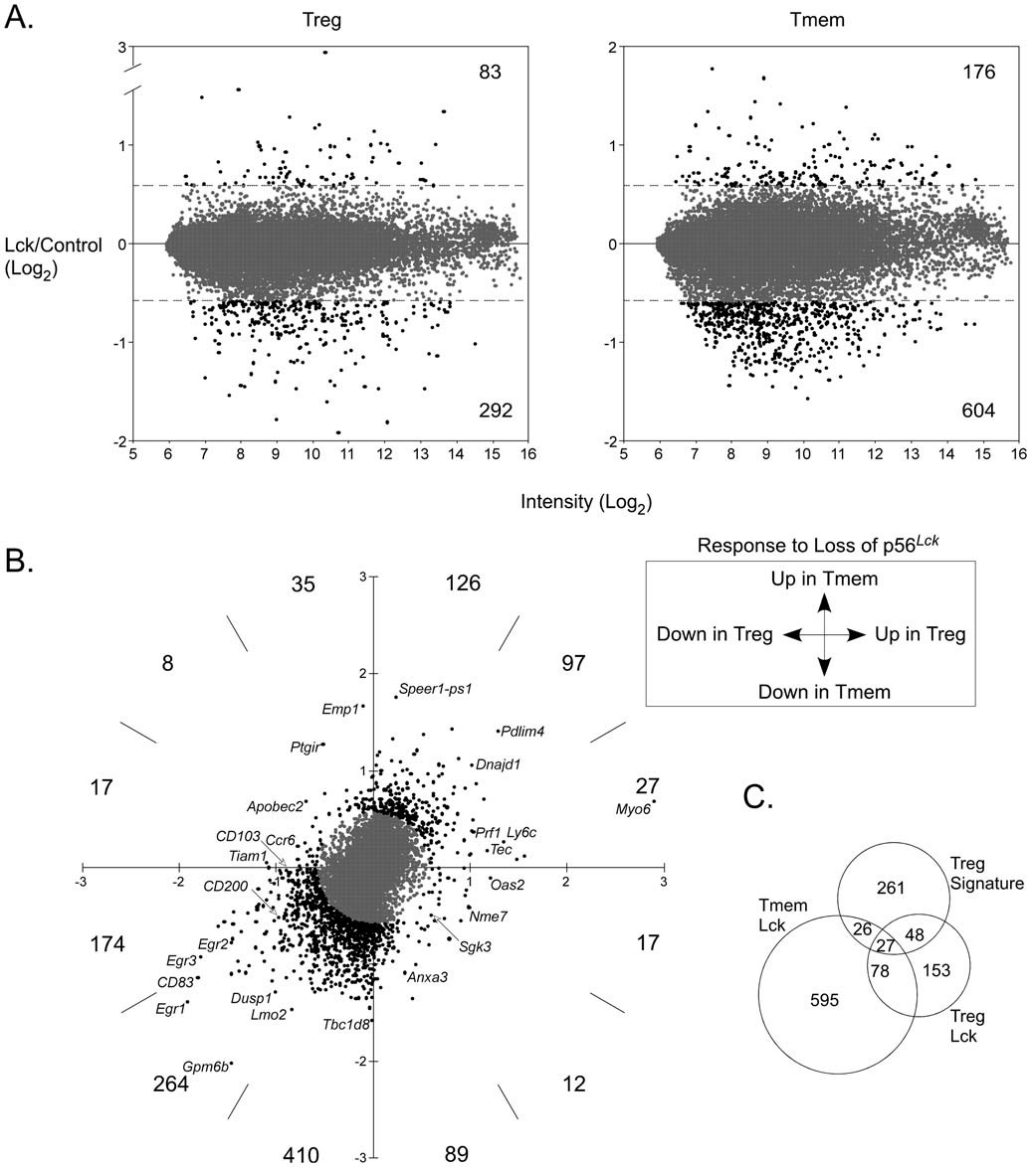
Gene expression in *Lck*-mutant regulatory T cells. A. The graphs show normalized log2 ratios of hybridization signals (Lck/Control) versus average hybridization intensity for all spots on the microarrays. Ratios corresponding to greater than 1.5-fold changes in spot intensity (dotted lines) are colored black instead of grey, and the numbers of these are indicated at the top and bottom right of the graphs. RNA was purified from flow-sorted Treg cells (CD4^+^CD25^+^) labeled differentially according to its origin (Lck mutant vs. control) and hybridized to microarrays spotted with 70-mer oligos from the MEEBO collection as described in Methods and Materials. B. Bivariate plot of Lck/Control spot ratios in memory versus regulatory T cells. Numbers of spots with vectors greater than 1.5 (colored black instead of grey) in the indicated parts of the four quadrants are shown. C. Venn diagram showing overlap between genes that are differentially expressed in control versus Lck memory and/or Treg cells and the Treg signature gene set identified by Hill *et al*. [40].

We identified a collection of genes that changed significantly in their expression in regulatory and/or memory T cells as a consequence of p56*^Lck^* deficiency (Figure 6A and B, Table S1). 20% of these genes increased in expression in both types of p56*^Lck^*-deficient T cells (Figure 6B top right quadrant). An even smaller proportion (13%) registered an increase in one or the other, but not both populations (Figure 6B). In marked contrast, the dominant consequence of the deficiency (in 67% of cases) was a reduction in expression in both types of T cell (Figure 6B bottom left quadrant). In general, therefore, the data showed that p56*^Lck^*, and thus the TCR signal, is more important for maintaining gene expression than for repressing it.

Among the genes that emerged as differentially expressed in the presence versus the absence of p56*^Lck^*, there were many whose regulation had previously been linked to the TCR stimulus in other studies (*e.g.*, genes encoding Egr-1, Egr-2, Egr-3, CD83, c-Myb, CD200, CD44, ALCAM and Stra13). Consistent with this, the differentially expressed collection from both memory and Treg cells was significantly enriched for genes with gene ontology (GO) term annotation related to lymphocyte activation (Table S2). Other enriched categories for both populations included immune system development, cell differentiation, immune response, signaling and apoptosis. The majority of the significantly enriched terms were associated with genes that were downregulated following inactivation of p56*^Lck^* and thus were located in the lower left quadrant of the plot in Figure 6B (q3 in Table S3).

Several studies have identified genes that are differentially expressed in Treg cells compared to conventional naïve T cells [37]–[41]. From a representative set of such genes [40] we identified 362 that were also represented on the microarray we used. Twenty-one percent of these (*i.e.*, 75/362) registered>1.5 fold changes in expression in Treg cells in the absence of p56*^Lck^* (Figure 6C, Table S4). In 73/75 cases, the changes were in the direction of diminishing the Treg signature, *i.e.*, in reducing the difference between Treg cells and conventional naïve T cells. 59 of the 75 genes (i.e., 79%) were normally upregulated in Treg cells, and all of these registered a reduction in expression in p56*^Lck^*-deficient T cells. Similarly, 14 genes were normally downregulated in Treg cells, and these showed upregulation in the absence of p56*^Lck^*. These observations reveal a significant component of the Treg signature as dependent on p56*^Lck^*, and thus point to sustained/basal TCR signaling as important for maintenance of this aspect of the Treg phenotype.

**Figure 7:**
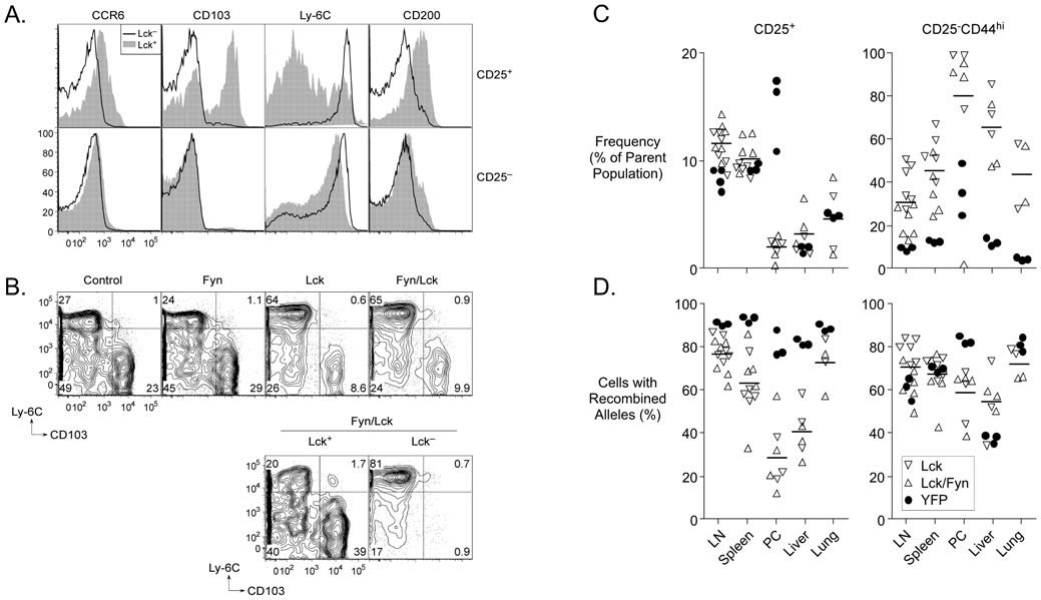
Phenotype and localization of *Lck*-mutant T cells. A. Flow cytometry validation of selected gene expression differences identified by microarray analysis. The overlayed histograms show expression of the indicated molecules in CD25^+^ (top) or CD25^−^ (bottom) T cells that retain (filled curve) or lack (open curve) expression of functional p56*^Lck^* taken from the lymph nodes of Lck mice. B. Expression of Ly-6C and CD103 on cells from mice of the indicated genotypes (top panel) and in cells that retain or lack expression of p56*^Lck^* from Lck/Fyn mice (bottom panel). C. Frequencies of regulatory (CD25^+^) or memory (CD25^−^CD44^hi^) phenotype cells in the indicated locations in Lck, Lck/Fyn or Ox40-cre/YFP mice. Horizontal bars represent means of the combined Lck and Lck/Fyn data for each location. D. Frequencies of cells with recombined alleles resulting in inactivation of p56*^Lck^* or acquisition of YFP in the same mice and populations studied in C.

Selected microarray data were corroborated by other procedures such as reverse transcriptase PCR analysis of mRNA, and flow cytometry. Figure 7A shows representative FACS data for four of the differentially expressed proteins (CCR6, CD103, Ly-6C, and CD200). Strikingly, CD25^+^ cells in Lck or Lck/Fyn mice were markedly enriched for high expression of Ly-6C and low expression of CD103 (Figure 7B). By contrast, cells that retained p56*^Lck^* expression in the same mice retained a near-normal pattern of CD103 and Ly-6C expression (Figure 7B, lower panel) indicating that the effect was intrinsic to cells lacking the kinase and not a consequence of changes in cytokine levels or other systemic extrinsic effects.

CCR6 and CD103 expression distinguish Treg cells with an effector/memory-like phenotype that is induced on activation [41], [42]. Both molecules have been implicated in controlling Treg cell migration [41], [43], [44]. Similarly, there is evidence that Ly-6C expression is of significance for homing of lymphocytes [45]. Given differential expression of these and other molecules involved in cell migration, it was of interest to determine whether loss of the TCR signal due to inactivation of p56*^Lck^* expression impacted Treg cell migration and hence the distribution of Treg cells in the body. To address this, we examined the representation of cells that had lost p56*^Lck^* (due to inactivation of one copy of the *Lck* gene by *Ox40-cre*; the other allele being congenitally null) in various tissues, and compared this to the representation of cells in other mice that had acquired YFP expression (due to *Ox40-cre*-dependent recombination of one copy of the *ROSA26* YFP reporter allele). Expression of YFP was expected to be without significant consequence for Treg cells and should not impact their distribution in the body. Thus the frequency of YFP^+^ cells was a useful reference point against which to look for effects of p56*^Lck^* deficiency.

As mentioned earlier, Treg cells lacking p56*^Lck^* were more abundant in the lymph nodes than in the spleen. This was consistent with the fact that loss of p56*^Lck^* was associated with high expression of CD62L (Figure 5) and a lack of upregulation of molecules such as CD103, CXCR3, the β1 integrin, Tiam1, RGS9 and CCR6, which are all of significance for emigration to tissues or retention in them (Figure 7AB, Table S1) [42], [46]–[48]. The frequency of CD25^+^ T cells as a fraction of the CD4^+^ population of T cells was near normal in the lymph nodes, spleens, livers and lungs of Lck and Lck/Fyn mice, but it was greatly reduced in their peritoneal cavities (Figure 7C). Much more strikingly, we noted a large decrease in the representation of p56*^Lck^*-deficient Treg cells (relative to p56*^Lck^*-expressing Treg cells) in the peritoneal cavity in particular, although a less pronounced effect was evident in all tissues examined (Figure 7D). Collectively, these data point to a critical role for TCR signaling in the regulation of Treg cell migration throughout the body and implicate specific cell surface receptors in this process.

Finally, we noted that CD25^−^CD44^hi^ CD4^+^ T cells were present at elevated frequencies in all tissues examined in both Lck and Lck/Fyn mice (Figure 7C) and the majority of these cells had undergone *Ox40-cre*-mediated inactivation of p56*^Lck^* (Figure 7D). Unlike Treg cells (and with the exception of CD44^hi^ CD4^+^ T cells in the peritoneal cavity) there was no apparent selection for retention of p56*^Lck^* function in this population. Like Treg cells, however, proliferating cells were significantly underrepresented among the CD44^hi^ cells that had undergone recombination (Figures 4B and C) suggesting that their increased numbers could not be explained by a shorter cell cycle. The lack of selective expansion of p56*^Lck^*-deficient cells instead suggested that the increased representation of the CD44^hi^ population was likely to be a cell-extrinsic consequence of impaired regulatory T cell activity.

## Discussion

Inactivation of p56*^Lck^* in Treg cells using *Ox40-cre* and a conditional null allele of the *Lck* gene blocks their capacity to signal through their TCRs and mediate antigen-directed suppression. It also incurs substantial changes in their basal gene expression program contributing to their abnormal distribution in the body. The deficiency blocks proliferation of Treg cells and homeostatic expansion, but it does not markedly impair their survival. The results make clear the importance of the TCR signal for Treg cell function, phenotype and homeostasis.

An induced loss of p56*^Lck^* in peripheral T cells has previously been engineered in mice through complementation of a congenital deficiency with a tetracycline-regulated *Lck* transgene [31]. Naïve p56*^Lck^*-deficient T cells in these mice showed long-term survival but an inability to expand following transfer into lymphopenic recipients. Survival depended on persistence of p59*^Fyn^* function in the naïve T cells, because in its absence the half-life of the cells was reduced 4-5-fold, and the cells also gained extreme sensitivity to blockade of IL-7:IL-7Rα interactions [18]. Basal TCRξ phosphorylation was substantially reduced in T cells lacking p56*^Lck^*, but it was undetectable when both p56*^Lck^* and p59*^Fyn^* were missing [18]. Together with data from other studies [19], [21], [49]–[57] these results indicate that TCR and IL-7 receptor signaling are essential for the survival of naïve T cells. By contrast, p56*^Lck^* deficiency (or combined p56*^Lck^* and p59*^Fyn^* deficiency) was found to have no detectable impact on the survival of memory T cells except in the presence of IL-7 receptor blockade, in which case CD4^+^ memory T cells, but not CD8^+^ T cells, showed impaired survival [20]. We show here that Treg cells persist in the absence of both p56*^Lck^* and p59*^Fyn^*, and in this respect they are therefore more similar to memory T cells than to naïve T cells.

Treg cells develop in the absence of IL-7 [58]. They are also detectable in IL-7Rα-deficient mice [7], [59], although in one of two recent reports they were found to be present in reduced numbers relative to IL-7^−/−^ mice and were also incapable of *in vitro* suppression [59]. These last data raised the possibility that Treg cell development and homeostasis might depend on both IL-7 and thymic stromal lymphopoietin (TSLP), both of which deliver signals through IL-7Rα [60], [61]. Whether Treg cells lacking the TCR signal might show enhanced sensitivity to IL-7Rα and/or TSLPR blockade has not yet been examined, but the mice described in this study would now allow for this.

Deletion of Treg cells results in autoimmune disease. This was initially apparent from studies involving the adoptive transfer of Treg cell-depleted naïve T cells into irradiated recipients [62], or from the analysis of mice that received neonatal thymectomies [62]–[64]. More recently, it has been made strikingly clear by the rapidly fatal disease that develops in genetically engineered mice that feature induced selective depletion of Treg cells [16]. Inactivation of TCR signaling in Treg cells should be expected to result in disease because even though Treg cells can develop (Figure 3) they cannot mediate antigen-directed suppression (Figure 4). Both Lck and Lck/Fyn mice were, however, free of obvious disease throughout a year-long observation period and we noted no increase in unexpected fatalities in the mutant groups relative to controls. Evidence that the mice were nonetheless undergoing immune responses due to impaired Treg cell function included mild lymphadenopathy (Figure 3) and increased frequencies of CD44^hi^CD25^−^CD4^+^ T cells (Figure 7C). These latter T cells lacked expression of activation markers, and were not actively proliferating, indicating that the immune responses that created them were not ongoing. The majority of them had undergone *Ox40-cre*-mediated inactivation of p56*^Lck^* (data not shown) providing a straightforward explanation for why they were not acutely activated, and suggesting that they were the product of clonal expansion that was likely terminated at the point when OX40 was induced. CD8^+^CD44^hi^ T cell numbers were not significantly elevated in the mutant mice, but we nonetheless detected an increase in the frequency of such cells that had undergone *Ox40-cre*-mediated recombination relative to what was observed in *Ox40-cre*/YFP mice (data not shown) again consistent with atypical activation due to impaired Treg cell function. Although the absence of disease in the mutant mice limited their usefulness for studying disease suppression by Treg cells, it was an obvious advantage for studying the Treg cell-intrinsic consequences of a loss of TCR signaling in the absence of complicating immunopathology.

Among the most notable consequences of p56*^Lck^* inactivation in Treg cells there was a substantial change in the steady-state pattern of gene expression. This was immediately evident at the cell surface (Figures 7 and 9) where the loss of p56*^Lck^* function correlated with dramatic changes in the display of several molecules. Most notably, compared to control cells, there was upregulation of Ly-6C and downregulation of CD103, CD200, CCR6, CD5, CD71, GITR, CD5, CD44 and CTLA4. Consistent with decreased levels of surface and intracellular molecules that influence migration to tissues and high levels of surface CD62L, Treg cells that had lost p56*^Lck^* function were under-represented in tissues and the spleen, but were present in normal frequencies in the lymph nodes. Whereas gene expression and distribution in the body were abnormal in p56*^Lck^*-deficient Treg cells, we found no obvious defects in p56*^Lck^*-sufficient cells in the same mice. Thus, the changes observed were intrinsic to Treg cells that had undergone *Ox40-cre*-mediated recombination and were not the result of systemic effects.

Under normal conditions, Treg cells can be subdivided into those that have a ‘naïve-like’ phenotype and those that have an ‘effector/memory’ phenotype [41]. Activation of the former causes at least some of them to differentiate into the latter and to mobilize from the lymph nodes to tissues and inflamed sites [41]. The correlation we have described between changes in gene expression and redistribution of Treg cells lacking p56*^Lck^* function are entirely consistent with this and indicate that the mobilization of Treg cells to tissues is a dynamic process that is strongly dependent on persistent TCR stimulation.

A large fraction of genes showed similar changes in expression in both Treg cells and conventional T cells following p56*^Lck^* inactivation (Figure 6B). These included genes that are well-characterized as being responsive to the TCR signal such as those encoding Egr-1, Egr-2, Egr-3, CD81, CD83, CD200, ALCAM, RANKL, and Stra13. It also included genes for CD137, CD44, Tieg1 and NRP1, variation in the expression of which has been correlated with Treg cell function [65]–[69]. Genes that were substantially affected in regulatory but not conventional T cells included those encoding Myosin-6, Egr-3, PD-1, Ptger2, Galnt14, Tox, RGS10, LIP1, Ly6f, Txk, and Tec. We noted that among an independently-derived list of Treg signature genes [40], approximately 20% were differentially expressed in the mutant Treg cells. While this indicates that a substantial part of the Treg phenotype (measured at the population level) is dependent on persistent TCR signaling, it also shows that perhaps the majority of the Treg gene expression pattern is independent of this. In particular, we found that although there was a modest reduction in FoxP3 transcript levels, this decrease was not readily apparent by intracellular staining for FoxP3 protein. Thus, sustained FoxP3 expression in Treg cells, like that of many other Treg signature genes, was relatively insensitive to loss of the TCR signal.

Two of the more striking consequences of p56*^Lck^* inactivation in Treg cells were the upregulation of Ly-6C and myosin-6 expression. The former is a cell surface molecule of unclear function that has the capacity to influence lymphocyte homing, while the latter is an actin-based motor with unusual properties [45], [70]. Upregulation of both molecules was initially detected in the microarray analysis, but was subsequently confirmed by FACS and real-time RT-PCR respectively. Although the significance of the upregulation of these genes in the mutant Treg cells remains to be established, it is possible that monitoring their expression (and that of other genes identified in this study) could be exploited as a means to identify cells in nonmutant populations of Treg cells that are experiencing less TCR signaling than other cells in the same populations. Such a discrimination might reveal unexpected relationships between self-responses and Treg cell function.

In almost all of the circumstances we examined, the absence of p59*^Fyn^* had no evident additive effect to the inactivation of p56*^Lck^*. Thus, Treg cells were present at the same average frequency in all locations in both Lck and Lck/Fyn mice, and their cell surface phenotype was indistinguishable in both cases. The only difference we noted between the two types of mice was in the reduced frequency of memory phenotype CD25^−^ CD4^+^ T cells in the lymph nodes of Lck/Fyn compared to Lck mice (Figure 7C). This decrease was associated with an increased apparent selection against cells with loss of p56*^Lck^* (Figure 7D). p56*^Lck^* and p59*^Fyn^* have partially overlapping functions at the CD4^−^CD8^−^ double-negative stage of development in the thymus [28], [29]. They also both positively regulate Erk activation in naïve CD4^+^ T cells in response to TCR ligation [71] and they are at least partially redundant in regulating naïve T cell survival [18]. Nonetheless, they phosphorylate distinct substrates and there is evidence that p59*^Fyn^* may be more important for attenuation of T cell signaling than for its propagation particularly in CD8^+^ T cells [72]. Thus, the absence of a more profound consequence of the double deficiency is not unexpected given the primacy of p56*^Lck^* in positive regulation of TCR signaling.

In conclusion, we have shown here that inactivation of p56*^Lck^* – and thus the TCR signal – incapacitates Treg cells and results in changes in their gene expression program. Mutant Treg cells distribute themselves abnormally in the body and although they do not proliferate, they nonetheless are capable of long-term survival. The results make clear aspects of the phenotype of Treg cells that are directly dependent on the TCR signal, while also revealing the utility of the *Ox40-cre* allele for conditional mutagenesis in the Treg lineage.

## Materials and Methods

### Ethics Statement

All experiments involving animals were performed according to protocols approved by the UCSF Institutional Animal Care and Use Committee.

### Mice

*Ox40-cre* mice were generated by gene targeting and contain an open reading frame encoding the cre recombinase inserted into exon 3 of the *Ox40* gene [25]. The neomycin resistance gene used for selection of ES cells was removed by Flp recombination in the germline. The *Ox40-cre* locus expresses Cre in place of OX40; this study employed mice that were heterozygous for the *cre* allele and demonstrated haploid expression of OX40 from a wild-type *Ox40* allele. Heterozygous levels of OX40 are not associated with immunological defects [30]. ROSA26-*loxP-*Stop-*loxP*-YFP mice were kindly provided by Dr. F. Costantini (Columbia University, New York, NY). The conditional null allele of the *Lck* gene has *loxP* sites upstream of the translation initiation codon in exon 1 and downstream of exon 3, and was also generated by gene targeting in ES cells (Benjamin *et al.* in preparation). Through use of a cryptic mRNA splice site, the Cre-recombined form of this allele allows for translation of a truncated form of p56*^Lck^* lacking the amino-terminal myristylation sequence, the CD4/CD8-interaction domain and the epitope recognized by the 1F6 monoclonal antibody. This truncated form of the protein is generated from thymic transcripts that derive from the proximal promoter of the *Lck* gene, but not from peripheral T cell transcripts deriving from the distal promoter. Mice homozygous for the Cre-recombined *Lck* allele manifest a phenotype that is identical to those of other p56*^Lck^*-deficient mice [73] showing that the truncated thymus-expressed protein is not functional, and thus the unrecombined allele can be used as a conditional null allele (Benjamin *et al.*, in preparation). Mice lacking expression of p59*^Fyn^* [74] were provided by Dr. Clifford Lowell. TCRβδ -deficient mice [75] were purchased from the Jackson Laboratory (Bar Harbor, ME). All mice were maintained under specific-pathogen free conditions.

### Antibodies, Flow Cytometry, and Cell Sorting

Conjugated antibodies were purchased from BD-Biosciences (San Diego, CA), Caltag Laboratories (San Francisco, CA), and eBioscience (San Diego, CA). FACS analysis was performed using FACSCalibur and LSR II flow cytometers (Becton Dickinson, Palo Alto, CA). A monoclonal antibody specific for the amino-terminal region of p56*^Lck^* (clone 1F6) originally generated by Dr. J. Bolen was generously provided by Dr. A. Weiss (UCSF). Single-cell suspensions were prepared from tissues using 0.45 µm cell strainers (Falcon; BD Biosciences) and PBS containing BSA (0.3%, w/v). Percoll fractionation was employed in some cases. Sorting was performed using a FACSAria (Becton Dickinson, Palo Alto, CA) to a final cell purity of≥95%. For cell cycle analysis, cells were stained with antibodies specific for cell surface antigens and fixed for 15 minutes in cytofix/cytoperm solution (BD Biosciences). The fixed cells were then washed in perm/wash solution (BD Biosciences) before incubation for at least 10 minutes in perm/wash solution containing DAPI (14 µM; Sigma) prior to analysis by flow cytometry.

### T Cell Proliferation Assays

CD4^+^CD25^−^ cells (2×10^4^ per well, U-bottom 96-well plate) and variable numbers of CD4^+^CD25^+^ cells were incubated together for 72 hours in the presence of irradiated (2000 rad) spleen cells (8×10^4^ per well) and anti-CD3 (clone 145–2C11, 2 µg/ml). ^3^H-thymidine (0.5 µCi/well; Du Pont/NEN) was added during the last 8 hours of culture. Background counts in wells containing APCs alone were always<1,000 cpm.

Flow-sorted CD4^+^CD25^−^ or CD4^+^CD25^+^ cells were labeled with CFSE (2 µM) and washed before intravenous injection into TCRβδ-deficient mice.

### Quantitative PCR to detect FoxP3 expression

Total RNA was extracted from 5×10^5^ flow-sorted cells using StrataPrep Total RNA Microprep kits (Stratagene, La Jolla, CA). To eliminate contaminating genomic DNA, the RNA preparations were treated with RNase-free DNase I (Invitrogen, Carlsbad, CA) and reverse-transcribed using Superscript II reverse-transcriptase and oligo(dT)12–18 as primer (Invitrogen). Real-time PCR was performed using Taqman probes for HPRT and FoxP3 using an MJ Chromo4 machine. Assays were performed in triplicate, analyzed using the ΔΔCt method [76], and repeated four times.

#### Microarray analysis

RNA was prepared from 1–2 million flow-sorted CD4^+^CD25^+^ and CD4^+^CD25^−^CD44^hi^ cells using the RNeasy mini kit (Qiagen) with on-column DNAse digestion. AminoAllyl-modified amplified RNA (aRNA) was prepared from approximately 1 µg of total RNA using the AminoAllyl Message Amp II kit (Ambion). 15 µg of this aRNA in 0.05 M sodium bicarbonate pH 9.0 were then coupled to N-hydroxysuccinimidyl esters of Cy3 or Cy5 dyes (CyScribe, Amersham Biosciences) for 90 minutes in the dark. The labelled aRNA was neutralized with 100 mM sodium acetate pH 5.5 and purified using the RNA clean-up kit-25 (Zymo Research). Immediately prior to hybridization, labelled aRNA samples were fragmented (Ambion Fragmentation buffer, Ambion) and denatured at 95°C for 10 minutes. Hybridizations to Mouse Exonic Evidence Based Oligonucleotide (MEEBO) spotted microarrays were performed at 55°C for 48 hours in Slide Hybridization buffer (Ambion). A total of 16 arrays were used to analyze regulatory and memory subsets from eight mice (two arrays per littermate pair, including dye swaps, were used for each comparison). After hybridization, the arrays were washed and scanned using an Axon 4000B laser scanner (Molecular Devices). Image analysis was performed using Spotreader (Niles Scientific). The ‘print-tip loess’ normalization [77] was used to correct for within-array dye and spatial effects and single channel quantile normalization was used to facilitate comparison between arrays [78]. Functions in the library marray Norm of the R/Bioconductor package were used to perform these normalizations [79]. After normalization the Log ratio, Log_2_(mutant/control), for each feature on each array was determined. No background subtraction was performed. Collections of differentially expressed genes were identified by selecting those genes that were up- or down-regulated by greater than 1.5 fold in Treg or memory T cells with inactivated p56*^Lck^*, and for which the B statistic [80] for the microarray data was greater than zero. A list of Treg cell signature genes [40] was compared with the entire MEEBO gene list using Gene Name as the identifier, yielding 362 genes in common. [81]). The microarray data discussed in this publication are MIAME compliant and have been deposited in NCBI's Gene Expression Omnibus [82] and are accessible through GEO Series accession number GSE13645 (http://www.ncbi.nlm.nih.gov/geo/query/acc.cgi?acc=GSE13645).

## Supporting Information

Figure S1 Enforced quiescence due to inactivation of p56*^Lck^*. A. Flow cytometry analysis of T cells in the spleens of T cell-deficient (Tcrβδ^−/−^) recipient mice one month after intravenous transfer of CD25^+^ or CD25^−^ T cells. CD4^+^ T cells (pooled from the lymph nodes and spleens) from mice of the four indicated genotypes were flow-sorted, and labeled with CFSE before transfer (1×10^6^ cells per recipient). B. Frequencies of BrdU^+^ cells (CD25^+^ or CD25^−^) in mice at the indicated times after the end of a two-week in vivo BrdU labeling period. Cells in Lck and Lck/Fyn mice that had or had not undergone *Ox40-cre*-mediated inactivation of the *Lck* gene were distinguished from one another on the basis of differential surface expression of CD4 (Figure 5). C. Mitotic status of regulatory and memory phenotype T cells in mice of the indicated genotypes. Cells were labeled with DAPI for cell cycle analysis, and with antibodies against surface molecules, p56*^Lck^* and FoxP3. The histograms show the percentages of cells in S, G2 or M phases of the cell cycle.
(0.26 MB TIF)

Figure S2 Regulatory T cells lacking CD4 expression. The graph at left shows the frequencies of FoxP3^+^ Treg cells in the spleens and lymph nodes of mice that differ in whether they feature *Ox40-cre*-dependent inactivation of the *Cd4* gene. The graph at right shows the frequencies of Treg cells with an activated phenotype.
(0.10 MB TIF)

Table S1 Microarray analysis of gene expression in memory and regulatory T cells with inactivated p56*^Lck^*. A. The spreadsheet shows the gene expression ratios (Log2(mutant/control)) and B statistics for individual microarray features from the analysis of Treg cells. B. The spreadsheet shows the gene expression ratios (Log2(mutant/control)) and B statistics for individual microarray features from the analysis of memory T cells.
(0.18 MB PDF)

Table S2 Gene ontology term analysis of differentially expressed genes in memory and regulatory T cells with inactivated p56*^Lck^*. Genes identified as differentially expressed (>1.5 fold fold-change and B statistic>0) in p56*^Lck^*-deficient cells compared to control cells were analyzed for enriched gene ontology (GO) annotation terms relative to the entire mouse genome using the DAVID Functional Annotation Tools [81] (April 2008 release). p-values for enrichment were obtained using a modified Fisher Exact test with adjustment for multiple sampling (Bonferroni). The table shows biological process GO terms (greater than level 1) associated with p-values less than 0.05; terms referring to related functions were grouped then ranked according to fold change (degree of over-representation of the term in the query list relative to representation in the mouse genome).
(0.05 MB PDF)

Table S3 Gene ontology term analysis of differentially expressed genes in memory and regulatory T cells with inactivated p56*^Lck^*. Differentially expressed genes (fold change>1.5) were grouped according to their location in the plot shown in Figure 6B. Quadrants in the figure were labeled sequentially from upper right (q1) clockwise to upper left (q4) and the numbers of differentially expressed genes in each quadrant are shown in brackets at the top of the table. Enrichment of gene ontology terms was determined as in Table 1 except that adjustment for multiple sampling was only used for the analysis of genes in quadrant 3.
(0.03 MB PDF)

Table S4 Treg signature genes affected by p56*^Lck^* inactivation. A. 362 genes common to the microarrays used in this study and those reported in Hill et al. [40]. B. 75 of the genes in Table S4A that showed>1.5 fold change in expression due to inactivation of p56*^Lck^*. 61 genes that are normally upregulated in Treg cells compared to conventional T cells are separated from 14 genes (at the bottom of the list) that are normally downregulated in Treg cells. 59 of the 61 genes were expressed at reduced levels in the absence of p56*^Lck^* than in its presence (i.e., the loss of p56*^Lck^* diminished the Treg signature); the two genes that showed the opposite behavior are listed in italics. All 14 genes that are normally downregulated in Treg cells showed increased expression in the absence of p56*^Lck^* than in its presence (i.e., again, the loss of p56*^Lck^* reduced the Treg signature).
(0.13 MB PDF)
