# Supplementary material for: Impact of the TCR Signal on Regulatory T Cell Homeostasis, Function, and Trafficking
Source: PLoS One. 2009 Aug 11;4(8):e6580. doi: 10.1371/journal.pone.0006580 (PMC2719063; doi:10.1371/journal.pone.0006580)
Supplement: Table S1 — Microarray analysis of gene expression in memory and regulatory T cells with inactivated p56Lck. A. The spreadsheet shows the gene expression ratios (Log2(mutant/control)) and B statistics for individual microarray features from the analysis of Treg cells. B. The spreadsheet shows the gene expression ratios (Log2(mutant/control)) and B statistics for individual microarray features from the analysis of memory T cells. (0.18 MB PDF) [file pone.0006580.s003.pdf]

Table S1A

Symbol	GeneID	Treg fold change (log_2)	B statistic	Description
Egr1	**13653**	**-1.92**	14.83	early growth response 1
Cd83	**12522**	**-1.81**	11.96	CD83 antigen
Egr3	**13655**	**-1.78**	26.1	early growth response 3
Myb	**17863**	**-1.6**	10.67	myeloblastosis oncogene
2610204G07Rik	**72468**	**-1.54**	17.97	RIKEN cDNA 2610204G07 gene
Tnfrsf4	**22163**	**-1.47**	10.28	tumor necrosis factor receptor superfamily, member 4
Gpm6b	**14758**	**-1.47**	8.11	glycoprotein m6b
Egr2	**13654**	**-1.45**	16.18	early growth response 2
Bmyc	**107771**	**-1.45**	17.98	brain expressed myelocytomatosis oncogene
Rad51l1	**19363**	**-1.44**	14.73	RAD51-like 1 (S. cerevisiae)
2610042L04Rik	**67055**	**-1.44**	3.54	RIKEN cDNA 2610042L04 gene
Nrn1	**68404**	**-1.4**	13.93	neuritin 1
Nr4a1	**15370**	**-1.39**	9.54	nuclear receptor subfamily 4, group A, member 1
C80638	**97086**	**-1.36**	10.2	expressed sequence C80638
1300007C21Rik	**67527**	**-1.32**	-1.59	RIKEN cDNA 1300007C21 gene
Dusp4	**319520**	**-1.3**	12.15	dual specificity phosphatase 4
Cybb	**13058**	**-1.28**	15.71	cytochrome b-245, beta polypeptide
Gsto1	**14873**	**-1.22**	10.95	glutathione S-transferase omega 1
Tnfrsf9	**21942**	**-1.21**	17.08	tumor necrosis factor receptor superfamily, member 9
Pdcd1	**18566**	**-1.2**	12.62	programmed cell death 1
A430093F15Rik	**403202**	**-1.2**	17.01	RIKEN cDNA A430093F15 gene
Tnfsf11	**21943**	**-1.19**	10.29	tumor necrosis factor (ligand) superfamily, member 11
1700001E04Rik	**75438**	**-1.18**	11.91	RIKEN cDNA 1700001E04 gene
Ctse	**13034**	**-1.15**	-2.98	cathepsin E
H2afz	**51788**	**-1.14**	15.24	H2A histone family, member Z
Ces2	**234671**	**-1.13**	7.97	carboxylesterase 2
Zfp36l1	**12192**	**-1.12**	18.99	zinc finger protein 36, C3H type-like 1
Tiam1	**21844**	**-1.1**	14.97	T-cell lymphoma invasion and metastasis 1
Samsn1	**67742**	**-1.1**	9.9	SAM domain, SH3 domain and nuclear localisation signals, 1
Nxf	**225872**	**-1.1**	9.01	bHLH-PAS type transcription factor NXF
Gsta4	**14860**	**-1.08**	11.42	glutathione S-transferase, alpha 4
2310001H17Rik	**76432**	**-1.08**	17.92	RIKEN cDNA 2310001H17 gene
Hod	**74318**	**-1.07**	12.47	homeobox only domain
Ptger2	**19217**	**-1.06**	13.4	prostaglandin E receptor 2 (subtype EP2)
Hmgn3	**94353**	**-1.05**	4.65	high mobility group nucleosomal binding domain 3
Tieg1	**21847**	**-1.03**	9.28	TGFB inducible early growth response 1
C030046G05	**327885**	**-1.03**	9.93	hypothetical protein C030046G05
Rgs9	**19739**	**-1.02**	16.1	regulator of G-protein signaling 9
Irf4	**16364**	**-1.02**	11.7	interferon regulatory factor 4
H2-Aa	**14960**	**-1.02**	3.83	histocompatibility 2, class II antigen A, alpha
Dusp1	**19252**	**-1.01**	13.76	dual specificity phosphatase 1
Rasip1	**69903**	**-0.98**	13.25	Ras interacting protein 1
Cd200	**17470**	**-0.98**	11.32	Cd200 antigen
2310067E08Rik	**71946**	**-0.98**	11.7	RIKEN cDNA 2310067E08 gene
Bcl2a1b	**12045**	**-0.97**	5.65	B-cell leukemia/lymphoma 2 related protein A1b
4930555G01Rik	**108978**	**-0.97**	10.81	RIKEN cDNA 4930555G01 gene
Galnt14	**71685**	**-0.96**	10.08	UDP-N-acetyl-alpha-D-galactosamine:polypeptide N-acetylgalactosaminyltransferase 14
Fcer2a	**14128**	**-0.96**	15.48	Fc receptor, IgE, low affinity II, alpha polypeptide
Ephx1	**13849**	**-0.96**	7.73	epoxide hydrolase 1, microsomal
Ptprs	**19280**	**-0.95**	7.06	protein tyrosine phosphatase, receptor type, S
Tacstd1	**17075**	**-0.94**	13.67	tumor-associated calcium signal transducer 1
Cd44	**12505**	**-0.94**	16.78	CD44 antigen
Blnk	**17060**	**-0.94**	5.14	B-cell linker
Bcl2a1d	**12047**	**-0.94**	7.11	B-cell leukemia/lymphoma 2 related protein A1d
Man1c1	**230815**	**-0.92**	14.32	mannosidase, alpha, class 1C, member 1
Bcl2a1a	**12044**	**-0.92**	7.09	B-cell leukemia/lymphoma 2 related protein A1a
Alcam	**11658**	**-0.92**	11.81	activated leukocyte cell adhesion molecule
2300009N04Rik	**69477**	**-0.92**	11.12	RIKEN cDNA 2300009N04 gene
Scd1	**20249**	**-0.91**	7.65	stearoyl-Coenzyme A desaturase 1
Kif27	**75050**	**-0.91**	-3.11	kinesin family member 27
Sh3gl3	**20408**	**-0.9**	8.16	SH3-domain GRB2-like 3
Penk1	**18619**	**-0.9**	2.04	preproenkephalin 1
Itgae	**NA**	**-0.9**	8.21	Mus musculus integrin, alpha E, epithelial-associated (Itgae), mRNA.
BC022623	**224093**	**-0.9**	3.43	cDNA sequence BC022623
Tox	**252838**	**-0.89**	8.8	thymocyte selection-associated HMG box gene
Swap70	**20947**	**-0.89**	13.65	SWAP complex protein
Eno3	**13808**	**-0.89**	12.6	enolase 3, beta muscle
Stmn1	**16765**	**-0.88**	2.12	stathmin 1
Rec8L1	**56739**	**-0.88**	7.62	REC8-like 1 (yeast)
Gucy1a3	**60596**	**-0.88**	11.77	guanylate cyclase 1, soluble, alpha 3
Cyfip1	**20430**	**-0.88**	16.83	cytoplasmic FMR1 interacting protein 1
1810037B05Rik	**69169**	**-0.88**	15.13	RIKEN cDNA 1810037B05 gene
Nt5e	**23959**	**-0.87**	3.66	5' nucleotidase, ecto
Twsg1	**65960**	**-0.86**	6.64	twisted gastrulation homolog 1 (Drosophila)
Serpina3g	**20715**	**-0.86**	7.7	serine (or cysteine) proteinase inhibitor, clade A, member 3G
Mmd	**67468**	**-0.86**	6.62	monocyte to macrophage differentiation-associated
Itgae	**16407**	**-0.86**	14.33	integrin, alpha E, epithelial-associated
Rgs10	**67865**	**-0.85**	13.19	regulator of G-protein signalling 10
Wee1	**22390**	**-0.84**	4.72	wee 1 homolog (S. pombe)
Lmo2	**16909**	**-0.84**	8.13	LIM domain only 2
Kif11	**16551**	**-0.84**	0.1	kinesin family member 11
BC023488	**237221**	**-0.84**	8.48	cDNA sequence BC023488
A430109M19Rik	**77935**	**-0.84**	5.23	RIKEN cDNA A430109M19 gene
Tcte1l	**67117**	**-0.83**	9.18	t-complex-associated-testis-expressed 1-like
Stx11	**74732**	**-0.83**	7.6	syntaxin 11
Nrp1	**18186**	**-0.83**	11.21	neuropilin 1
1110032F04Rik	**68725**	**-0.83**	7.72	RIKEN cDNA 1110032F04 gene
1110020C13Rik	**66151**	**-0.83**	8.37	RIKEN cDNA 1110020C13 gene
Ndg2	**103172**	**-0.82**	7.29	Nur77 downstream gene 2
Cybb	**13058**	**-0.82**	7.89	cytochrome b-245, beta polypeptide

Table S1A

2810465F10Rik	**78771**	**-0.82**	8.64	RIKEN cDNA 2810465F10 gene
Ndg1	**368204**	**-0.81**	11.42	Nur77 downstream gene 1
Hif1a	**15251**	**-0.81**	5.33	hypoxia inducible factor 1, alpha subunit
Cr2	**12902**	**-0.81**	10.48	complement receptor 2
A130034K24Rik	**319774**	**-0.81**	11.36	RIKEN cDNA A130034K24 gene
Odc1	**18263**	**-0.8**	4.35	ornithine decarboxylase, structural 1
D16Bwg1543e	**52910**	**-0.8**	9.01	DNA segment, Chr 16, Brigham & Women's Genetics 1543 expressed
C230027N18Rik	**330940**	**-0.8**	0.19	RIKEN cDNA C230027N18 gene
4732472I07Rik	**271844**	**-0.8**	10.39	RIKEN cDNA 4732472I07 gene
Zfpn1a2	**22779**	**-0.79**	13.56	zinc finger protein, subfamily 1A, 2 (Helios)
Zfp36l1	**12192**	**-0.79**	7.46	zinc finger protein 36, C3H type-like 1
Src	**20779**	**-0.79**	7.46	Rous sarcoma oncogene
Scd2	**20250**	**-0.79**	13.03	stearoyl-Coenzyme A desaturase 2
Itgae	**NA**	**-0.79**	12.81	Mus musculus integrin, alpha E, epithelial-associated (Itgae), mRNA.
D930040M24Rik	**320858**	**-0.79**	-4.42	RIKEN cDNA D930040M24 gene
Anxa2	**12306**	**-0.79**	0.83	annexin A2
4631408O11Rik	**66693**	**-0.79**	6.92	RIKEN cDNA 4631408O11 gene
Nrp	**NA**	**-0.78**	11.52	Mus musculus neuropilin (Nrp), mRNA.
Itgae	**NA**	**-0.78**	11.15	Mus musculus integrin, alpha E, epithelial-associated (Itgae), mRNA.
Inpp5f	**101490**	**-0.78**	8.76	inositol polyphosphate-5-phosphatase F
Ebf1	**13591**	**-0.78**	0.54	early B-cell factor 1
Cst7	**13011**	**-0.78**	12.08	cystatin F (leukocystatin)
Cd79b	**NA**	**-0.78**	12.4	Mus musculus CD79B antigen (Cd79b), mRNA.
Ccr6	**12458**	**-0.78**	3.9	chemokine (C-C motif) receptor 6
Bcl2a1c	**12046**	**-0.78**	0.21	B-cell leukemia/lymphoma 2 related protein A1c
Axl	**26362**	**-0.78**	9.51	AXL receptor tyrosine kinase
Swap70	**20947**	**-0.77**	8.07	SWAP complex protein
Hivep3	**16656**	**-0.77**	10.3	human immunodeficiency virus type I enhancer binding protein 3
Hist1h2an	**319170**	**-0.77**	0.74	histone 1, H2an
4930431P03Rik	**73895**	**-0.77**	10.38	RIKEN cDNA 4930431P03 gene
4732460K03Rik	**58894**	**-0.77**	11.8	RIKEN cDNA 4732460K03 gene
Mef2c	**17260**	**-0.76**	6.06	myocyte enhancer factor 2C
Itgb1	**16412**	**-0.76**	2.48	integrin beta 1 (fibronectin receptor beta)
Dst	**NA**	**-0.76**	3.62	Mus musculus dystonin (Dst), mRNA.
Cxcr3	**12766**	**-0.76**	1.93	chemokine (C-X-C motif) receptor 3
Cks2	**66197**	**-0.76**	1.61	CDC28 protein kinase regulatory subunit 2
Bhlhb2	**20893**	**-0.76**	9.63	basic helix-loop-helix domain containing, class B2
BC003324	**80291**	**-0.76**	9.39	cDNA sequence BC003324
Rad51	**19361**	**-0.75**	-1.54	RAD51 homolog (S. cerevisiae)
Noxa1	**241275**	**-0.75**	16.22	NADPH oxidase activator 1
Lag3	**16768**	**-0.75**	6.17	lymphocyte-activation gene 3
Dnmt3a	**13435**	**-0.75**	0.93	DNA methyltransferase 3A
D930021L15Rik	**319420**	**-0.75**	0.98	RIKEN cDNA D930021L15 gene
Cd79b	**15985**	**-0.75**	7.94	CD79B antigen
Ccna2	**12428**	**-0.75**	-0.54	cyclin A2
AI324046	**380795**	**-0.75**	-1.52	expressed sequence AI324046
Irf5	**27056**	**-0.74**	6.62	interferon regulatory factor 5
H2-Ab1	**14961**	**-0.74**	1.4	histocompatibility 2, class II antigen A, beta 1
8030462D06Rik	**77174**	**-0.74**	15.38	RIKEN cDNA 8030462D06 gene
5031439A09Rik	**68151**	**-0.74**	4.08	RIKEN cDNA 5031439A09 gene
1110060M21Rik	**66208**	**-0.74**	6.69	RIKEN cDNA 1110060M21 gene
IGHD	**NA**	**-0.73**	6.04	Part of the mouse gene for delta-immunoglobulin (CH1 domain).
Glrx1	**93692**	**-0.73**	5.87	glutaredoxin 1 (thioltransferase)
Cd81	**12520**	**-0.73**	6.17	CD 81 antigen
9430025M21Rik	**77300**	**-0.73**	13.78	RIKEN cDNA 9430025M21 gene
0610010E21Rik	**68332**	**-0.73**	6.49	RIKEN cDNA 0610010E21 gene
Cks1	**54124**	**-0.72**	-2.32	CDC28 protein kinase 1
Acate2	**56360**	**-0.72**	4.54	acyl-Coenzyme A thioesterase 2, mitochondrial
A630026H08Rik	**NA**	**-0.72**	7.92	Mus musculus RIKEN cDNA A630026H08 gene (A630026H08Rik), mRNA.
Serpinc1	**11905**	**-0.71**	-1.72	serine (or cysteine) proteinase inhibitor, clade C (antithrombin), member 1
Rog	**58206**	**-0.71**	-0.54	repressor of GATA
LOC56304	**56304**	**-0.71**	1.26	recombinant antineuraminidase single chain Ig VH and VL domains
E130308A19Rik	**230259**	**-0.71**	7.97	RIKEN cDNA E130308A19 gene
Casp4	**12363**	**-0.71**	7.24	caspase 4, apoptosis-related cysteine protease
Tank	**NA**	**-0.7**	8.1	Mus musculus TRAF family member-associated Nf-kappa B activator (Tank), mRNA.
Slc7a10	**53896**	**-0.7**	5.72	solute carrier family 7 (cationic amino acid transporter, y+ system), member 10
Igk-C	**16071**	**-0.7**	-3.41	immunoglobulin kappa chain, constant region
Arhgap20	**244867**	**-0.7**	9.2	Rho GTPase activating protein 20
Apobec2	**11811**	**-0.7**	-0.99	apolipoprotein B editing complex 2
Trpm1	**17364**	**-0.69**	11.3	transient receptor potential cation channel, subfamily M, member 1
Rabgap1l	**29809**	**-0.69**	7.1	RAB GTPase activating protein 1-like
Nr4a2	**18227**	**-0.69**	9.89	nuclear receptor subfamily 4, group A, member 2
Knsl7	**209737**	**-0.69**	-1.13	kinesin-like 7
Hist1h2ah	**319168**	**-0.69**	0.61	histone 1, H2ah
H2-Oa	**15001**	**-0.69**	5.99	histocompatibility 2, O region alpha locus
H2-Ab1	**14961**	**-0.69**	0.67	histocompatibility 2, class II antigen A, beta 1
Gnpnat1	**54342**	**-0.69**	5.97	glucosamine-phosphate N-acetyltransferase 1
D3Ucla1	**28146**	**-0.69**	3.49	DNA segment, Chr 3, University of California at Los Angeles 1
Zfp36l1	**12192**	**-0.68**	4.33	zinc finger protein 36, C3H type-like 1
Sh3md2	**NA**	**-0.68**	2.48	Mus musculus SH3 multiple domains 2 (Sh3md2), transcript variant 2, mRNA.
Serf1	**20365**	**-0.68**	3.63	small EDRK-rich factor 1
Mbnl3	**171170**	**-0.68**	1.43	muscleblind-like 3 (Drosophila)
Hells	**15201**	**-0.68**	-1.23	helicase, lymphoid specific
Farp1	**223254**	**-0.68**	5.53	FERM, RhoGEF (Arhgef) and pleckstrin domain protein 1 (chondrocyte-derived)
Bcat1	**12035**	**-0.68**	6.66	branched chain aminotransferase 1, cytosolic
4931413I07Rik	**113875**	**-0.68**	-3.93	RIKEN cDNA 4931413I07 gene
Zfp52	**22710**	**-0.67**	14.36	zinc finger protein 52
Hist1h2ao	**319171**	**-0.67**	2.08	histone 1, H2ao
Hist1h2ag	**319167**	**-0.67**	0.93	histone 1, H2ag
Gpr56	**14766**	**-0.67**	5.6	G protein-coupled receptor 56
Gpm6a	**234267**	**-0.67**	-4.64	glycoprotein m6a
A330015K06Rik	**320190**	**-0.67**	-2.34	RIKEN cDNA A330015K06 gene

Table S1A

9930021J17Rik	**229488**	**-0.67**	5.96	RIKEN cDNA 9930021J17 gene
1110051B16Rik	**278672**	**-0.67**	-1.16	RIKEN cDNA 1110051B16 gene
Tnfrsf1b	**21938**	**-0.66**	8.81	tumor necrosis factor receptor superfamily, member 1b
Tbc1d4	**210789**	**-0.66**	2.32	TBC1 domain family, member 4
Plk3	**12795**	**-0.66**	0.88	polo-like kinase 3 (Drosophila)
Osbpl3	**71720**	**-0.66**	5.14	oxysterol binding protein-like 3
Mgmt	**17314**	**-0.66**	1.72	O-6-methylguanine-DNA methyltransferase
IGLC3	**NA**	**-0.66**	15.82	Mus musculus domesticus immunoglobulin lambda J3-C3-J1-C1 cluster, 3' flanking sequence.
IGKC	**NA**	**-0.66**	-3.11	Part of the germ line murine gene for kappa-immunoglobulin (last exon).
Bcl11a	**NA**	**-0.66**	9.7	Mus musculus B-cell CLL/lymphoma 11A (zinc finger protein), mRNA (cDNA clone IMAGE:2655319), complete cds.
Arhgap18	**73910**	**-0.66**	5.82	Rho GTPase activating protein 18
Rasgef1b	**320292**	**-0.65**	9.38	RasGEF domain family, member 1B
Ptpn5	**19259**	**-0.65**	8.13	protein tyrosine phosphatase, non-receptor type 5
Ppap2a	**19012**	**-0.65**	8.17	phosphatidic acid phosphatase 2a
Nelf	**56876**	**-0.65**	6.89	nasal embryonic LHRH factor
Mki67	**17345**	**-0.65**	0.68	antigen identified by monoclonal antibody Ki 67
Mastl	**67121**	**-0.65**	-4.25	microtubule associated serine/threonine kinase-like
LOC382646	**NA**	**-0.65**	3.8	Mus musculus similar to Ig delta chain C region, membrane-bound form - mouse (LOC382646), mRNA.
Gla	**11605**	**-0.65**	-2.9	galactosidase, alpha
Ear1	**13586**	**-0.65**	-2.37	eosinophil-associated, ribonuclease A family, member 1
C330008K14Rik	**77619**	**-0.65**	6.23	RIKEN cDNA C330008K14 gene
2410003J06Rik	**71967**	**-0.65**	19.14	RIKEN cDNA 2410003J06 gene
Tnfrsf13c	**72049**	**-0.64**	5.71	tumor necrosis factor receptor superfamily, member 13c
Rasgef1a	**70727**	**-0.64**	-1.63	RasGEF domain family, member 1A
Marveld2	**218518**	**-0.64**	3.67	MARVEL (membrane-associating) domain containing 2
Il1r2	**16178**	**-0.64**	2.61	interleukin 1 receptor, type II
Igk-V28	**16114**	**-0.64**	-4.07	immunoglobulin kappa chain variable 28 (V28)
Hist1h2ak	**319169**	**-0.64**	0	histone 1, H2ak
H2-Eb1	**14969**	**-0.64**	-0.35	histocompatibility 2, class II antigen E beta
Ear1	**13586**	**-0.64**	-2.25	eosinophil-associated, ribonuclease A family, member 1
A530030E21Rik	**320731**	**-0.64**	5.55	RIKEN cDNA A530030E21 gene
A230020G22Rik	**229499**	**-0.64**	11.57	RIKEN cDNA A230020G22 gene
Zdhhc2	**70546**	**-0.63**	0.24	zinc finger, DHHC domain containing 2
Tank	**21353**	**-0.63**	10.77	TRAF family member-associated Nf-kappa B activator
Sytl2	**83671**	**-0.63**	2	synaptotagmin-like 2
Sh3bgrl	**56726**	**-0.63**	8.15	SH3-binding domain glutamic acid-rich protein like
Sf3b1	**81898**	**-0.63**	-3.92	splicing factor 3b, subunit 1
Nkg7	**72310**	**-0.63**	-0.5	natural killer cell group 7 sequence
Ncf2	**17970**	**-0.63**	3.6	neutrophil cytosolic factor 2
Map17	**67182**	**-0.63**	14.71	membrane-associated protein 17
Kdt1	**16543**	**-0.63**	7.51	kidney cell line derived transcript 1
Igfbp7	**29817**	**-0.63**	2.63	insulin-like growth factor binding protein 7
Hist1h2ai	**319191**	**-0.63**	0.83	histone 1, H2ai
Hap1	**15114**	**-0.63**	5.52	huntingtin-associated protein 1
9030411K21Rik	**71557**	**-0.63**	-4.18	RIKEN cDNA 9030411K21 gene
5730453H04Rik	**105072**	**-0.63**	-2.89	RIKEN cDNA 5730453H04 gene
4932412D23Rik	**75722**	**-0.63**	-3.87	RIKEN cDNA 4932412D23 gene
Tns	**21961**	**-0.62**	0.48	tensin
Scrg3	**20286**	**-0.62**	2.03	scrapie responsive gene 3
Sat1	**20229**	**-0.62**	3.13	spermidine/spermine N1-acetyl transferase 1
Pon3	**269823**	**-0.62**	4.21	paraoxonase 3
IGKV6-c	**NA**	**-0.62**	-3.06	Mus musculus (SK/CamRK) germline IgK gene, V-region.
Gch1	**14528**	**-0.62**	-1.52	GTP cyclohydrolase 1
Eomes	**13813**	**-0.62**	8.77	eomesodermin homolog (Xenopus laevis)
E030024M05Rik	**217430**	**-0.62**	7.06	RIKEN cDNA E030024M05 gene
Ctla4	**12477**	**-0.62**	1.29	cytotoxic T-lymphocyte-associated protein 4
Alcam	**11658**	**-0.62**	10.27	activated leukocyte cell adhesion molecule
A530052I06Rik	**99953**	**-0.62**	4.29	RIKEN cDNA A530052I06 gene
6530404N21Rik	**67795**	**-0.62**	-0.41	RIKEN cDNA 6530404N21 gene
Rnase6	**78416**	**-0.61**	5.14	ribonuclease A family, member 6
Ndrg1	**17988**	**-0.61**	3.88	N-myc downstream regulated gene 1
IGKV6-32	**NA**	**-0.61**	-3.3	Mus musculus IgVk 19-32 gene.
Igk-V1	**16081**	**-0.61**	-1.7	immunoglobulin kappa chain variable 1 (V1)
Hist1h2ad	**319165**	**-0.61**	0.01	histone 1, H2ad
D730019B10Rik	**237256**	**-0.61**	2.71	RIKEN cDNA D730019B10 gene
Cd200r1	**57781**	**-0.61**	3.53	CD200 receptor 1
Cd19	**12478**	**-0.61**	5.78	CD19 antigen
C330023F11Rik	**109232**	**-0.61**	5.02	RIKEN cDNA C330023F11 gene
C2ta	**12265**	**-0.61**	-1.09	class II transactivator
Bcl6	**12053**	**-0.61**	14.74	B-cell leukemia/lymphoma 6
9630014C11Rik	**78570**	**-0.61**	0.88	RIKEN cDNA 9630014C11 gene
Samsn1	**67742**	**-0.6**	8.05	SAM domain, SH3 domain and nuclear localisation signals, 1
Reps1	**19707**	**-0.6**	7.02	RalBP1 associated Eps domain containing protein
Ly86	**17084**	**-0.6**	1.23	lymphocyte antigen 86
LOC382646	**NA**	**-0.6**	2.78	Mus musculus similar to Ig delta chain C region, membrane-bound form - mouse (LOC382646), mRNA.
Ii	**16149**	**-0.6**	-0.91	Ia-associated invariant chain
Icsbp1	**15900**	**-0.6**	1.79	interferon consensus sequence binding protein 1
Gstt3	**103140**	**-0.6**	7	glutathione S-transferase, theta 3
Gphn	**268566**	**-0.6**	4.89	gephyrin
Ddit4	**74747**	**-0.6**	3.52	DNA-damage-inducible transcript 4
Casp3	**12367**	**-0.6**	7.72	caspase 3, apoptosis related cysteine protease
A930031D07Rik	**213006**	**-0.6**	6.68	RIKEN cDNA A930031D07 gene
2310026E23Rik	**67621**	**-0.6**	4.24	RIKEN cDNA 2310026E23 gene
1200008O12Rik	**74107**	**-0.6**	-0.91	RIKEN cDNA 1200008O12 gene
Spry1	**24063**	**-0.59**	3.07	sprouty homolog 1 (Drosophila)
Spag5	**54141**	**-0.59**	-0.78	sperm associated antigen 5
Siat8d	**20452**	**-0.59**	9.2	sialyltransferase 8 (alpha-2, 8-sialyltransferase) D
Rmcs1	**114887**	**-0.59**	-0.94	response to metastatic cancers 1
Rln3	**212108**	**-0.59**	13.37	relaxin 3
Phtf2	**68770**	**-0.59**	6.5	putative homeodomain transcription factor 2
Olfr134	**258829**	**-0.59**	-4.19	olfactory receptor 134
Ear3	**53876**	**-0.59**	-1.03	eosinophil-associated, ribonuclease A family, member 3

Table S1A

Chi3l3	**12655**	**-0.59**	3.97	chitinase 3-like 3
Cd24a	**12484**	**-0.59**	-0.05	CD24a antigen
Capn3	**12335**	**-0.59**	5.72	calpain 3
Bcar3	**29815**	**-0.59**	4.35	breast cancer anti-estrogen resistance 3
5830456J23Rik	**76105**	**-0.59**	2.17	RIKEN cDNA 5830456J23 gene
2900026A02Rik	**243219**	**-0.59**	8.78	RIKEN cDNA 2900026A02 gene
Tgtp	**21822**	**0.59**	7.91	T-cell specific GTPase
Sgk3	**NA**	**0.59**	8	Mus musculus serum/glucocorticoid regulated kinase 3 (Sgk3), mRNA.
Satb1	**20230**	**0.59**	4.28	special AT-rich sequence binding protein 1
4930485D02Rik	**75812**	**0.59**	9.47	RIKEN cDNA 4930485D02 gene
1500004A08Rik	**216505**	**0.59**	7.54	RIKEN cDNA 1500004A08 gene
V2r1b	**56552**	**0.6**	-5.71	vomeronasal 2, receptor, 1b
D11Ertd759e	**268513**	**0.61**	6.57	DNA segment, Chr 11, ERATO Doi 759, expressed
C030002B11Rik	**78540**	**0.61**	4.94	RIKEN cDNA C030002B11 gene
9130427A09Rik	**107022**	**0.61**	5.02	RIKEN cDNA 9130427A09 gene
4632430A05Rik	**77587**	**0.61**	-2.81	RIKEN cDNA 4632430A05 gene
Oasl2	**23962**	**0.62**	6.37	2'-5' oligoadenylate synthetase-like 2
Lef1	**16842**	**0.62**	2.25	lymphoid enhancer binding factor 1
Grap2	**17444**	**0.62**	7.76	GRB2-related adaptor protein 2
Eraf	**170812**	**0.62**	0.03	erythroid associated factor
Art4	**109978**	**0.62**	9.84	ADP-ribosyltransferase 4
Lip1	**16889**	**0.63**	9.89	lysosomal acid lipase 1
1110007C02Rik	**71784**	**0.63**	3.79	RIKEN cDNA 1110007C02 gene
Daf1	**13136**	**0.64**	4.79	decay accelerating factor 1
1810045K07Rik	**67887**	**0.64**	10.33	RIKEN cDNA 1810045K07 gene
Slc28a2	**269346**	**0.65**	0.83	solute carrier family 28 (sodium-coupled nucleoside transporter), member 2
Igtp	**16145**	**0.65**	7.89	interferon gamma induced GTPase
E430024C06Rik	**319443**	**0.65**	-2.43	RIKEN cDNA E430024C06 gene
AW536289	**104645**	**0.65**	4.86	expressed sequence AW536289
Pdlim1	**54132**	**0.66**	3.95	PDZ and LIM domain 1 (elfin)
Crlf3	**54394**	**0.66**	4.35	cytokine receptor-like factor 3
Rcn1	**19672**	**0.67**	5.93	reticulocalbin 1
Pdk1	**228026**	**0.67**	15.3	pyruvate dehydrogenase kinase, isoenzyme 1
Ifi44	**99899**	**0.67**	-4.98	interferon-induced protein 44
Bclp2	**229687**	**0.67**	4.48	chitinase like protein 2
Actn2	**11472**	**0.67**	9.35	actinin alpha 2
Abca3	**27410**	**0.67**	-5.16	ATP-binding cassette, sub-family A (ABC1), member 3
2610019F03Rik	**72148**	**0.67**	12.6	RIKEN cDNA 2610019F03 gene
1810045K07Rik	**NA**	**0.67**	5.67	Mus musculus RIKEN cDNA 1810045K07 gene (1810045K07Rik), mRNA.
Zbp1	**58203**	**0.68**	0.45	Z-DNA binding protein 1
Igfbp4	**16010**	**0.68**	2.43	insulin-like growth factor binding protein 4
Adamts7	**108153**	**0.68**	-5.71	a disintegrin-like and metalloprotease (reprolysin type) with thrombospondin type 1 motif, 7
Herc1	**235439**	**0.69**	-5.77	hect (homologous to the E6-AP (UBE3A) carboxyl terminus) domain and RCC1 (CHC1)-like domain (RLD) 1
Slfn5	**327978**	**0.71**	2.21	schlafen 5
Gbp2	**14469**	**0.71**	5.44	guanylate nucleotide binding protein 2
AI661017	**213068**	**0.71**	9.05	expressed sequence AI661017
E430004N04Rik	**210757**	**0.72**	7.17	RIKEN cDNA E430004N04 gene
BC022645	**235633**	**0.73**	2.2	cDNA sequence BC022645
2610204G22Rik	**70448**	**0.73**	6.8	RIKEN cDNA 2610204G22 gene
1700012B18Rik	**71839**	**0.73**	8.81	RIKEN cDNA 1700012B18 gene
Trib3	**228775**	**0.74**	9.2	tribbles homolog 3 (Drosophila)
Ifit3	**15959**	**0.76**	0.88	interferon-induced protein with tetratricopeptide repeats 3
2010002M12Rik	**112419**	**0.76**	4.77	RIKEN cDNA 2010002M12 gene
Nme7	**NA**	**0.77**	12.49	Mus musculus non-metastatic cells 7, protein expressed in (Nme7), mRNA.
Tcrg-V4	**21638**	**0.78**	3.67	T-cell receptor gamma, variable 4
Pdlim4	**30794**	**0.81**	6.82	PDZ and LIM domain 4
Socs3	**12702**	**0.82**	3.59	suppressor of cytokine signaling 3
2310046K10Rik	**NA**	**0.82**	8.57	Mus musculus RIKEN cDNA 2310046K10 gene (2310046K10Rik), transcript variant a, mRNA.
Ifit3	**15959**	**0.83**	1.78	interferon-induced protein with tetratricopeptide repeats 3
Dntt	**21673**	**0.83**	10.52	deoxynucleotidyltransferase, terminal
A130092J06Rik	**241303**	**0.83**	5.48	RIKEN cDNA A130092J06 gene
Tyki	**22169**	**0.87**	1.31	thymidylate kinase family LPS-inducible member
Ifit3	**15959**	**0.87**	1.68	interferon-induced protein with tetratricopeptide repeats 3
5830431A10Rik	**76024**	**0.88**	5.47	RIKEN cDNA 5830431A10 gene
Igfbp4	**16010**	**0.93**	12.88	insulin-like growth factor binding protein 4
Ly6f	**17071**	**0.94**	19.96	lymphocyte antigen 6 complex, locus F
Tubb2	**22151**	**0.95**	17.1	tubulin, beta 2
Slc16a5	**217316**	**0.95**	10.83	solute carrier family 16 (monocarboxylic acid transporters), member 5
2310032F03Rik	**76747**	**0.96**	2.42	RIKEN cDNA 2310032F03 gene
Atp1b1	**11931**	**0.98**	11.38	ATPase, Na+/K+ transporting, beta 1 polypeptide
Sh3bgrl2	**212531**	**0.99**	14.56	SH3 domain binding glutamic acid-rich protein like 2
Nme7	**NA**	**0.99**	12.35	Mus musculus non-metastatic cells 7, protein expressed in (Nme7), mRNA.
Txk	**22165**	**1**	15.13	TXK tyrosine kinase
Ly6a	**110454**	**1.01**	7.31	lymphocyte antigen 6 complex, locus A
Dnajd1	**66148**	**1.01**	6.03	DnaJ (Hsp40) homolog, subfamily D, member 1
Epsti1	**108670**	**1.02**	10.66	epithelial stromal interaction 1 (breast)
Prf1	**18646**	**1.03**	13.95	perforin 1 (pore forming protein)
Ifit1	**15957**	**1.06**	-0.26	interferon-induced protein with tetratricopeptide repeats 1
1500005K14Rik	**76566**	**1.14**	10.75	RIKEN cDNA 1500005K14 gene
Tec	**21682**	**1.17**	12.8	cytoplasmic tyrosine kinase, Dscr28C related (Drosophila)
Oas2	**246728**	**1.21**	7.49	2'-5' oligoadenylate synthetase 2
Gzma	**14938**	**1.28**	-1.73	granzyme A
Ly6c	**17067**	**1.34**	11.85	lymphocyte antigen 6 complex, locus C
BC029719	**270179**	**1.48**	11.6	cDNA sequence BC029719
Bclp2	**229687**	**1.56**	19.52	chitinase like protein 2
Myo6	**17920**	**2.89**	19.95	myosin VI

Table S1B

Symbol	GeneID	Tmem fold change (log_2)	B statistic	Description
Gpm6b	**14758**	**-2.01**	12.58	glycoprotein m6b
Tbc1d8	**54610**	**-1.57**	13.69	TBC1 domain family, member 8
Lmo2	**16909**	**-1.46**	16.11	LIM domain only 2
AW046396	**329173**	**-1.45**	16.89	expressed sequence AW046396
2610012C04Rik	**70299**	**-1.44**	12.46	RIKEN cDNA 2610012C04 gene
Egr1	**13653**	**-1.38**	10.17	early growth response 1
Il4i1	**14204**	**-1.35**	6.96	interleukin 4 induced 1
2210421G13Rik	**108956**	**-1.35**	7.66	RIKEN cDNA 2210421G13 gene
Serpinb10	**241197**	**-1.34**	5.01	serine (or cysteine) proteinase inhibitor, clade B (ovalbumin), member 10
Rtn1	**104001**	**-1.34**	11.93	reticulon 1
Mmp12	**17381**	**-1.33**	11.16	matrix metalloproteinase 12
C230002E15Rik	**319708**	**-1.33**	10.72	RIKEN cDNA C230002E15 gene
Flt3	**14255**	**-1.29**	16.81	FMS-like tyrosine kinase 3
2210409B22Rik	**70174**	**-1.29**	12.82	RIKEN cDNA 2210409B22 gene
Rnase6	**78416**	**-1.28**	15.38	ribonuclease A family, member 6
Dusp1	**19252**	**-1.28**	17.4	dual specificity phosphatase 1
Kmo	**98256**	**-1.26**	19.48	kynurenine 3-monooxygenase (kynurenine 3-hydroxylase)
Emr1	**13733**	**-1.26**	14.55	EGF-like module containing, mucin-like, hormone receptor-like sequence 1
Hap1	**15114**	**-1.24**	14.98	huntingtin-associated protein 1
2900022I03Rik	**72849**	**-1.24**	13.68	RIKEN cDNA 2900022I03 gene
Adam11	**11488**	**-1.23**	2.86	a disintegrin and metalloprotease domain 11
Igsf6	**80719**	**-1.22**	14.03	immunoglobulin superfamily, member 6
Ccnd1	**12443**	**-1.22**	10.25	cyclin D1
Basp1	**70350**	**-1.22**	13.39	brain abundant, membrane attached signal protein 1
Fos	**14281**	**-1.21**	3.11	FBJ osteosarcoma oncogene
Kynu	**70789**	**-1.2**	11.6	kynureninase (L-kynurenine hydrolase)
Klrb1c	**17059**	**-1.2**	7.78	killer cell lectin-like receptor subfamily B member 1C
Gpr124	**NA**	**-1.2**	5.53	Mus musculus G protein-coupled receptor 124 (Gpr124), mRNA.
Ube2e2	**218793**	**-1.19**	9.39	ubiquitin-conjugating enzyme E2E 2 (UBC4/5 homolog, yeast)
Scap2	**54353**	**-1.19**	12.05	src family associated phosphoprotein 2
Ear1	**13586**	**-1.19**	3.58	eosinophil-associated, ribonuclease A family, member 1
Dscam	**13508**	**-1.19**	10.76	Down syndrome cell adhesion molecule
2900052N01Rik	**73040**	**-1.19**	17.07	RIKEN cDNA 2900052N01 gene
Klf4	**16600**	**-1.18**	16.23	Kruppel-like factor 4 (gut)
A130001G05Rik	**320704**	**-1.18**	10.86	RIKEN cDNA A130001G05 gene
4921513O20Rik	**66725**	**-1.18**	14.03	RIKEN cDNA 4921513O20 gene
2010002N04Rik	**106878**	**-1.18**	9.3	RIKEN cDNA 2010002N04 gene
Rp2h	**19889**	**-1.17**	10.39	retinitis pigmentosa 2 homolog (human)
Ogfrl1	**70155**	**-1.17**	10.67	opioid growth factor receptor-like 1
Hmgn3	**94353**	**-1.17**	5.89	high mobility group nucleosomal binding domain 3
0610012D17Rik	**66061**	**-1.17**	13.18	RIKEN cDNA 0610012D17 gene
Spint1	**20732**	**-1.16**	15.86	serine protease inhibitor, Kunitz type 1
BC035044	**232406**	**-1.16**	8.07	cDNA sequence BC035044
Sash1	**70097**	**-1.15**	14.96	SAM and SH3 domain containing 1
Igsf7	**140497**	**-1.15**	16.72	immunoglobulin superfamily, member 7
5031439G07Rik	**223739**	**-1.15**	12.8	RIKEN cDNA 5031439G07 gene
Hspa1b	**15511**	**-1.14**	-5.21	heat shock protein 1B
Ear1	**13586**	**-1.14**	2.82	eosinophil-associated, ribonuclease A family, member 1
D930021L15Rik	**319420**	**-1.14**	5.82	RIKEN cDNA D930021L15 gene
BC035044	**232406**	**-1.14**	9.04	cDNA sequence BC035044
Asb2	**65256**	**-1.14**	11.16	ankyrin repeat and SOCS box-containing protein 2
Cd83	**12522**	**-1.13**	5.4	CD83 antigen
BC022623	**224093**	**-1.13**	6.21	cDNA sequence BC022623
Adam23	**23792**	**-1.13**	8.48	a disintegrin and metalloprotease domain 23
Dnase1l3	**13421**	**-1.12**	11.26	deoxyribonuclease 1-like 3
Sort1	**20661**	**-1.11**	7.35	sortilin 1
Sort1	**20661**	**-1.11**	5.97	sortilin 1
Ryr3	**20192**	**-1.11**	17.97	ryanodine receptor 3
Rp2h	**19889**	**-1.11**	13.32	retinitis pigmentosa 2 homolog (human)
Ptger3	**19218**	**-1.11**	8.38	prostaglandin E receptor 3 (subtype EP3)
Hck	**15162**	**-1.11**	17.02	hemopoietic cell kinase
Sirpb	**320832**	**-1.1**	8.74	signal-regulatory protein beta
E130112L23Rik	**268739**	**-1.1**	9.96	RIKEN cDNA E130112L23 gene
1100001H23Rik	**66857**	**-1.1**	6.94	RIKEN cDNA 1100001H23 gene
Tnfrsf4	**22163**	**-1.09**	6.22	tumor necrosis factor receptor superfamily, member 4
Marcks	**17118**	**-1.09**	14.91	myristoylated alanine rich protein kinase C substrate
Fgr	**14191**	**-1.08**	10.43	Gardner-Rasheed feline sarcoma viral (Fgr) oncogene homolog
Anxa3	**11745**	**-1.08**	7.73	annexin A3
1300007C21Rik	**67527**	**-1.08**	-3.1	RIKEN cDNA 1300007C21 gene
Ncf2	**17970**	**-1.07**	10.56	neutrophil cytosolic factor 2
Fcer1g	**14127**	**-1.07**	12.93	Fc receptor, IgE, high affinity I, gamma polypeptide
9930027N05Rik	**NA**	**-1.07**	12.64	Mus musculus RIKEN cDNA 9930027N05 gene (9930027N05Rik), transcript variant 2, mRNA.
Rgs18	**64214**	**-1.06**	8.07	regulator of G-protein signaling 18
Plxnc1	**54712**	**-1.06**	12.04	plexin C1
Nr4a1	**15370**	**-1.06**	5.9	nuclear receptor subfamily 4, group A, member 1
Cybb	**13058**	**-1.06**	11.45	cytochrome b-245, beta polypeptide
Bcl11a	**NA**	**-1.06**	16.59	Mus musculus B-cell CLL/lymphoma 11A (zinc finger protein), mRNA (cDNA clone IMAGE:2655319), complete cds.
Slamf8	**74748**	**-1.05**	10.94	SLAM family member 8
Mpeg1	**17476**	**-1.05**	5.77	macrophage expressed gene 1
Ifitm6	**213002**	**-1.05**	5.49	interferon induced transmembrane protein 6
Gsn	**NA**	**-1.05**	10.77	Mus musculus gelsolin (Gsn), mRNA.
Gpr124	**78560**	**-1.05**	15.99	G protein-coupled receptor 124
C030046G05	**327885**	**-1.05**	10.14	hypothetical protein C030046G05
Arhgap6	**11856**	**-1.05**	4.8	Rho GTPase activating protein 6
6430529G09Rik	**233274**	**-1.05**	3.89	RIKEN cDNA 6430529G09 gene
Sulf2	**72043**	**-1.04**	16.24	sulfatase 2
BC023928	**268377**	**-1.04**	9.88	cDNA sequence BC023928
App	**11820**	**-1.04**	7.8	amyloid beta (A4) precursor protein
Npl	**74091**	**-1.03**	13.85	N-acetylneuraminate pyruvate lyase
Gpr43	**233079**	**-1.03**	9.92	G protein-coupled receptor 43

Table S1B

Plek	**56193**	**-1.02**	12.23	pleckstrin
9630014C11Rik	**78570**	**-1.02**	6.92	RIKEN cDNA 9630014C11 gene
Tm6sf1	**107769**	**-1.01**	14.37	transmembrane 6 superfamily member 1
Kctd12	**239217**	**-1.01**	9.69	potassium channel tetramerisation domain containing 12
Cybb	**13058**	**-1.01**	12.46	cytochrome b-245, beta polypeptide
Clecsf12	**56644**	**-1.01**	7.38	C-type (calcium dependent, carbohydrate recognition domain) lectin, superfamily member 12
9830002I17Rik	**77577**	**-1.01**	10.48	RIKEN cDNA 9830002I17 gene
2510048K03Rik	**72461**	**-1.01**	14.82	RIKEN cDNA 2510048K03 gene
2210020M01Rik	**66528**	**-1.01**	3.79	RIKEN cDNA 2210020M01 gene
Rhoq	**104215**	**-1**	8.01	ras homolog gene family, member Q
Arhgap22	**239027**	**-1**	10.21	Rho GTPase activating protein 22
TRGC4	**NA**	**-0.99**	0.51	Mus musculus T cell receptor gamma locus, TCR gamma 2 and gamma 4 gene clusters.
Sat1	**20229**	**-0.99**	9.16	spermidine/spermine N1-acetyl transferase 1
BC003277	**214359**	**-0.99**	8.03	cDNA sequence BC003277
A530045L16Rik	**319528**	**-0.99**	0.26	RIKEN cDNA A530045L16 gene
A330066M24Rik	**320357**	**-0.99**	9.31	RIKEN cDNA A330066M24 gene
Pmaip1	**58801**	**-0.98**	10.88	phorbol-12-myristate-13-acetate-induced protein 1
Irf5	**27056**	**-0.98**	10.45	interferon regulatory factor 5
Dna2l	**327762**	**-0.98**	5.92	DNA2 DNA replication helicase 2-like (yeast)
Csf2ra	**12982**	**-0.98**	13.24	colony stimulating factor 2 receptor, alpha, low-affinity (granulocyte-macrophage)
2410025L10Rik	**381668**	**-0.98**	1.95	RIKEN cDNA 2410025L10 gene
Kcnk6	**52150**	**-0.97**	7.67	potassium inwardly-rectifying channel, subfamily K, member 6
Dkk2	**56811**	**-0.97**	5.16	dickkopf homolog 2 (Xenopus laevis)
Ctsh	**13036**	**-0.97**	5.13	cathepsin H
Cd81	**12520**	**-0.97**	10	CD 81 antigen
9030208C03Rik	**320131**	**-0.97**	6.25	RIKEN cDNA 9030208C03 gene
Tyrobp	**22177**	**-0.96**	6.71	TYRO protein tyrosine kinase binding protein
Nrn1	**68404**	**-0.96**	8.68	neuritin 1
Ckb	**12709**	**-0.96**	10	creatine kinase, brain
Ces2	**234671**	**-0.96**	5.72	carboxylesterase 2
Cd209a	**170786**	**-0.96**	15.05	CD209a antigen
1200006F02Rik	**71706**	**-0.96**	12.06	RIKEN cDNA 1200006F02 gene
Tcf4	**21413**	**-0.95**	6.37	transcription factor 4
Rgs2	**19735**	**-0.95**	7.68	regulator of G-protein signaling 2
Ptpns1	**19261**	**-0.95**	7.34	protein tyrosine phosphatase, non-receptor type substrate 1
Lrrc16	**68732**	**-0.95**	13.8	leucine rich repeat containing 16
Cd63	**12512**	**-0.95**	5.95	Cd63 antigen
2810451E09Rik	**72821**	**-0.95**	16.14	RIKEN cDNA 2810451E09 gene
1600010M07Rik	**69781**	**-0.95**	11.09	RIKEN cDNA 1600010M07 gene
Zfhx1b	**24136**	**-0.94**	8.59	zinc finger homeobox 1b
Tgfbi	**21810**	**-0.94**	7.81	transforming growth factor, beta induced
Lrp1	**NA**	**-0.94**	5.79	Mus musculus low density lipoprotein receptor-related protein 1 (Lrp1), mRNA.
Dab2ip	**69601**	**-0.94**	12.75	disabled homolog 2 (Drosophila) interacting protein
C2ta	**12265**	**-0.94**	3.29	class II transactivator
6330407A03Rik	**70720**	**-0.94**	7.19	RIKEN cDNA 6330407A03 gene
Rab32	**67844**	**-0.93**	14.36	RAB32, member RAS oncogene family
Mllt4	**17356**	**-0.93**	7.62	myeloid/lymphoid or mixed lineage-leukemia translocation to 4 homolog (Drosophila)
Hist1h1c	**50708**	**-0.93**	18.7	histone 1, H1c
H2-DMb1	**14999**	**-0.93**	8.62	histocompatibility 2, class II, locus Mb1
E030003N15Rik	**105841**	**-0.93**	12.44	RIKEN cDNA E030003N15 gene
Dock7	**67299**	**-0.93**	3.77	dedicator of cytokinesis 7
B130040O20Rik	**320962**	**-0.93**	10.2	RIKEN cDNA B130040O20 gene
AI132321	**104759**	**-0.93**	6.48	expressed sequence AI132321
Rnf149	**67702**	**-0.92**	5.94	ring finger protein 149
Gcnt2	**14538**	**-0.92**	10.58	glucosaminyl (N-acetyl) transferase 2, I-branching enzyme
Egr3	**13655**	**-0.92**	18.81	early growth response 3
BC049354	**269799**	**-0.92**	9.49	cDNA sequence BC049354
A530088I07Rik	**212167**	**-0.92**	9.11	RIKEN cDNA A530088I07 gene
Tlr13	**279572**	**-0.91**	6.85	toll-like receptor 13
Rgs1	**50778**	**-0.91**	2.83	regulator of G-protein signaling 1
Lyzs	**17105**	**-0.91**	0.59	lysozyme
Igk-C	**16071**	**-0.91**	-1.79	immunoglobulin kappa chain, constant region
Hhex	**15242**	**-0.91**	14.95	hematopoietically expressed homeobox
D930015E06Rik	**229473**	**-0.91**	14.99	RIKEN cDNA D930015E06 gene
Ccnd1	**12443**	**-0.91**	6.65	cyclin D1
Slc8a1	**20541**	**-0.9**	8.46	solute carrier family 8 (sodium/calcium exchanger), member 1
Slamf7	**75345**	**-0.9**	15.93	SLAM family member 7
Pla2g4a	**18783**	**-0.9**	8.8	phospholipase A2, group IVA (cytosolic, calcium-dependent)
Phca	**66190**	**-0.9**	10.55	phytoceramidase, alkaline
Lrrk1	**233328**	**-0.9**	12.07	leucine-rich repeat kinase 1
Itgax	**16411**	**-0.9**	15.18	integrin alpha X
Il1b	**16176**	**-0.9**	11.92	interleukin 1 beta
Frmd4a	**209630**	**-0.9**	4.34	FERM domain containing 4A
Dnmt3a	**13435**	**-0.9**	2.9	DNA methyltransferase 3A
Ccnd1	**12443**	**-0.9**	14.6	cyclin D1
Bcl11a	**14025**	**-0.9**	7.21	B-cell CLL/lymphoma 11A (zinc finger protein)
Pira4	**18727**	**-0.89**	8.93	paired-Ig-like receptor A4
Pfc	**18636**	**-0.89**	6.17	properdin factor, complement
Gpr82	**319200**	**-0.89**	16.43	G protein-coupled receptor 82
Apg4c	**242557**	**-0.89**	12.82	APG4 (ATG4) autophagy-related homolog C (S. cerevisiae)
Anxa4	**11746**	**-0.89**	9.24	annexin A4
Havcr2	**171285**	**-0.88**	8.38	hepatitis A virus cellular receptor 2
Card12	**268973**	**-0.88**	14.79	caspase recruitment domain family, member 12
1810009M01Rik	**65963**	**-0.88**	6.62	RIKEN cDNA 1810009M01 gene
Sucnr1	**84112**	**-0.87**	7.03	succinate receptor 1
Spry1	**24063**	**-0.87**	8.03	sprouty homolog 1 (Drosophila)
Sirpb	**320832**	**-0.87**	7.15	signal-regulatory protein beta
Rassf4	**213391**	**-0.87**	12.84	Ras association (RalGDS/AF-6) domain family 4
Qk	**19317**	**-0.87**	10.52	quaking
Mdh2	**17448**	**-0.87**	5.57	malate dehydrogenase 2, NAD (mitochondrial)
Kdr	**16542**	**-0.87**	5.18	kinase insert domain protein receptor
Ctse	**13034**	**-0.87**	-4.55	cathepsin E

Table S1B

A430090E18Rik	**195564**	**-0.87**	12.89	RIKEN cDNA A430090E18 gene
2810442I21Rik	**72735**	**-0.87**	5.54	RIKEN cDNA 2810442I21 gene
TRGV1	**NA**	**-0.86**	-0.91	Mouse T-cell receptor germline gamma chain gene V-region (V108B).
Tns	**21961**	**-0.86**	3.97	tensin
IGKV8-24	**NA**	**-0.86**	0.98	Mus musculus IgVk 8-24 gene.
H2-Eb1	**14969**	**-0.86**	2.66	histocompatibility 2, class II antigen E beta
Ddhd1	**114874**	**-0.86**	1.92	DDHD domain containing 1
1810033B17Rik	**69189**	**-0.86**	1.41	RIKEN cDNA 1810033B17 gene
1110055L24Rik	**66205**	**-0.86**	5.32	RIKEN cDNA 1110055L24 gene
Swap70	**20947**	**-0.85**	13.06	SWAP complex protein
Rmcs1	**114887**	**-0.85**	2.55	response to metastatic cancers 1
Ramp1	**51801**	**-0.85**	3.66	receptor (calcitonin) activity modifying protein 1
Ptprs	**19280**	**-0.85**	5.51	protein tyrosine phosphatase, receptor type, S
Plcg2	**234779**	**-0.85**	14.35	phospholipase C, gamma 2
Mgl1	**17312**	**-0.85**	5.99	macrophage galactose N-acetyl-galactosamine specific lectin 1
Ifi30	**65972**	**-0.85**	6.35	interferon gamma inducible protein 30
H2-Oa	**15001**	**-0.85**	8.93	histocompatibility 2, O region alpha locus
Clic4	**29876**	**-0.85**	10.45	chloride intracellular channel 4 (mitochondrial)
B630019K06Rik	**102941**	**-0.85**	5.98	RIKEN cDNA B630019K06 gene
6230421P05Rik	**109033**	**-0.85**	5.6	RIKEN cDNA 6230421P05 gene
2900024D24Rik	**72925**	**-0.85**	13.66	RIKEN cDNA 2900024D24 gene
Vav2	**22325**	**-0.84**	13.33	Vav2 oncogene
Tacstd1	**17075**	**-0.84**	12.06	tumor-associated calcium signal transducer 1
Rasgrp3	**240168**	**-0.84**	9.93	RAS, guanyl releasing protein 3
Lzf	**66049**	**-0.84**	14.85	leucine zipper domain protein
LOC329416	**329416**	**-0.84**	12.57	nitric oxide synthase trafficker
Igk-V28	**16114**	**-0.84**	-2.66	immunoglobulin kappa chain variable 28 (V28)
Ier5	**15939**	**-0.84**	9.37	immediate early response 5
Gpr120	**107221**	**-0.84**	7.98	G protein-coupled receptor 120
Ahrr	**11624**	**-0.84**	10.37	aryl-hydrocarbon receptor repressor
Xcl1	**16963**	**-0.83**	2.82	chemokine (C motif) ligand 1
Rem1	**19700**	**-0.83**	9.12	rad and gem related GTP binding protein 1
Pirb	**18733**	**-0.83**	4.34	paired-Ig-like receptor B
Klra12	**16630**	**-0.83**	-1.14	killer cell lectin-like receptor subfamily A, member 12
Blnk	**17060**	**-0.83**	3.61	B-cell linker
BC023105	**207269**	**-0.83**	6.57	cDNA sequence BC023105
Adam23	**NA**	**-0.83**	4.54	Mus musculus a disintegrin and metalloprotease domain 23 (Adam23), mRNA.
A130095M15Rik	**319928**	**-0.83**	6.02	RIKEN cDNA A130095M15 gene
2310057H16Rik	**67951**	**-0.83**	12.76	RIKEN cDNA 2310057H16 gene
Tnfrsf5	**21939**	**-0.82**	13.16	tumor necrosis factor receptor superfamily, member 5
Pik3cb	**74769**	**-0.82**	9.12	phosphatidylinositol 3-kinase, catalytic, beta polypeptide
Nucb2	**53322**	**-0.82**	7.46	nucleobindin 2
Klra20	**93967**	**-0.82**	-1.84	killer cell lectin-like receptor subfamily A, member 20
IGHV1S56	**NA**	**-0.82**	2.59	Mus musculus immunoglobin heavy chain precursor (IgH J558-43Y) gene, V-region, partial cds.
H2-Aa	**14960**	**-0.82**	1.35	histocompatibility 2, class II antigen A, alpha
Gsto1	**14873**	**-0.82**	5.45	glutathione S-transferase omega 1
Ear10	**93725**	**-0.82**	1.87	eosinophil-associated, ribonuclease A family, member 10
Cyp27a1	**104086**	**-0.82**	7.08	cytochrome P450, family 27, subfamily a, polypeptide 1
Ctss	**13040**	**-0.82**	8.34	cathepsin S
Bcl6	**12053**	**-0.82**	19.16	B-cell leukemia/lymphoma 6
AW490415	**101361**	**-0.82**	10.96	expressed sequence AW490415
Zfhx1b	**24136**	**-0.81**	10.52	zinc finger homeobox 1b
Slc6a12	**14411**	**-0.81**	8.24	solute carrier family 6 (neurotransmitter transporter, betaine/GABA), member 12
Nphp3	**74025**	**-0.81**	-0.08	nephronophthisis 3 (adolescent)
Ly86	**17084**	**-0.81**	4.6	lymphocyte antigen 86
Cd209d	**170779**	**-0.81**	2.36	CD209d antigen
Catnd2	**18163**	**-0.81**	6.49	catenin delta 2
B230311A21	**328314**	**-0.81**	13.51	hypothetical protein B230311A21
Alcam	**11658**	**-0.81**	10.02	activated leukocyte cell adhesion molecule
AI426953	**103207**	**-0.81**	1.62	expressed sequence AI426953
4631426J05Rik	**77590**	**-0.81**	15.32	RIKEN cDNA 4631426J05 gene
2810046M22Rik	**269633**	**-0.81**	8.73	RIKEN cDNA 2810046M22 gene
1110060D06Rik	**68813**	**-0.81**	5.17	RIKEN cDNA 1110060D06 gene
Tns	**21961**	**-0.8**	4.86	tensin
Siat4c	**20443**	**-0.8**	15.61	sialyltransferase 4C (beta-galactoside alpha-2,3-sialytransferase)
Rasgef1b	**320292**	**-0.8**	12.34	RasGEF domain family, member 1B
Pip5k2a	**18718**	**-0.8**	9.54	phosphatidylinositol-4-phosphate 5-kinase, type II, alpha
Lzp-s	**17110**	**-0.8**	0.68	P lysozyme structural
Ifngr2	**15980**	**-0.8**	11.84	interferon gamma receptor 2
Csf2rb2	**12984**	**-0.8**	6.66	colony stimulating factor 2 receptor, beta 2, low-affinity (granulocyte-macrophage)
Chka	**12660**	**-0.8**	9.57	choline kinase alpha
Cd80	**12519**	**-0.8**	4.34	CD80 antigen
1110032F04Rik	**68725**	**-0.8**	7.24	RIKEN cDNA 1110032F04 gene
Siat8f	**241230**	**-0.79**	3.44	sialyltransferase 8 (alpha-2, 8-sialyltransferase) F
Serpina3g	**20715**	**-0.79**	6.38	serine (or cysteine) proteinase inhibitor, clade A, member 3G
Ppfia4	**68507**	**-0.79**	14.59	protein tyrosine phosphatase, receptor type, f polypeptide (PTPRF), interacting protein (liprin), alpha 4
Pira1	**18722**	**-0.79**	9.28	paired-Ig-like receptor A1
Mef2c	**17260**	**-0.79**	6.56	myocyte enhancer factor 2C
Lmo4	**16911**	**-0.79**	0.47	LIM domain only 4
IGKC	**NA**	**-0.79**	-2.07	Part of the germ line murine gene for kappa-immunoglobulin (last exon).
Fgr	**14191**	**-0.79**	14.61	Gardner-Rasheed feline sarcoma viral (Fgr) oncogene homolog
Fgd2	**26382**	**-0.79**	6.9	FYVE, RhoGEF and PH domain containing 2
Fes	**14159**	**-0.79**	4.91	feline sarcoma oncogene
D630012G11Rik	**320904**	**-0.79**	5.83	RIKEN cDNA D630012G11 gene
Cerk	**223753**	**-0.79**	9.81	ceramide kinase
1810018P12Rik	**74170**	**-0.79**	6.36	RIKEN cDNA 1810018P12 gene
1810011H11Rik	**69069**	**-0.79**	3.18	RIKEN cDNA 1810011H11 gene
Ncf1	**17969**	**-0.78**	14.83	neutrophil cytosolic factor 1
Klra16	**27424**	**-0.78**	-1.17	killer cell lectin-like receptor, subfamily A, member 16
Igk-V1	**16081**	**-0.78**	0.39	immunoglobulin kappa chain variable 1 (V1)
Emr4	**52614**	**-0.78**	5.57	EGF-like module containing, mucin-like, hormone receptor-like sequence 4
Cd68	**12514**	**-0.78**	12.21	CD68 antigen

Table S1B

Alcam	**11658**	**-0.78**	13.6	activated leukocyte cell adhesion molecule
5730407K14Rik	**70479**	**-0.78**	11.57	RIKEN cDNA 5730407K14 gene
1300002K09Rik	**74152**	**-0.78**	13.46	RIKEN cDNA 1300002K09 gene
1110018F16Rik	**68594**	**-0.78**	5	RIKEN cDNA 1110018F16 gene
Lag3	**16768**	**-0.77**	6.45	lymphocyte-activation gene 3
Kctd12	**239217**	**-0.77**	9.11	potassium channel tetramerisation domain containing 12
Igsf4a	**54725**	**-0.77**	2.77	immunoglobulin superfamily, member 4A
Igh-VJ558	**16061**	**-0.77**	-4.23	immunoglobulin heavy chain (J558 family)
Hsd17b4	**15488**	**-0.77**	6.24	hydroxysteroid (17-beta) dehydrogenase 4
Cd86	**12524**	**-0.77**	10.94	CD86 antigen
Cbfa2t3h	**12398**	**-0.77**	12.79	core-binding factor, runt domain, alpha subunit 2, translocated to, 3 homolog (human)
Bcl2a1b	**12045**	**-0.77**	2.71	B-cell leukemia/lymphoma 2 related protein A1b
Avpi1	**69534**	**-0.77**	5.22	arginine vasopressin-induced 1
A930004K21Rik	**228564**	**-0.77**	6.07	RIKEN cDNA A930004K21 gene
Usp12	**22217**	**-0.76**	2.51	ubiquitin specific protease 12
Tgm2	**NA**	**-0.76**	9.91	Mus musculus transglutaminase 2, C polypeptide (Tgm2), mRNA.
St14	**19143**	**-0.76**	6.97	suppression of tumorigenicity 14 (colon carcinoma)
Rassf4	**NA**	**-0.76**	6.51	Mus musculus Ras association (RalGDS/AF-6) domain family 4 (Rassf4), mRNA.
Pla2g7	**27226**	**-0.76**	7.96	phospholipase A2, group VII (platelet-activating factor acetylhydrolase, plasma)
LOC193676	**193676**	**-0.76**	7.76	hypothetical protein LOC193676
Igk-V21	**16098**	**-0.76**	-3.15	immunoglobulin kappa chain variable 21 (V21)
Btk	**12229**	**-0.76**	11.24	Bruton agammaglobulinemia tyrosine kinase
Bcl2a1d	**12047**	**-0.76**	4.23	B-cell leukemia/lymphoma 2 related protein A1d
2900046G09Rik	**78408**	**-0.76**	8.24	RIKEN cDNA 2900046G09 gene
2810048G17Rik	**72691**	**-0.76**	6.14	RIKEN cDNA 2810048G17 gene
2310009N05Rik	**66943**	**-0.76**	9.43	RIKEN cDNA 2310009N05 gene
Syk	**20963**	**-0.75**	12.09	spleen tyrosine kinase
Npc1	**18145**	**-0.75**	4.51	Niemann Pick type C1
Lair1	**52855**	**-0.75**	0.9	leukocyte-associated Ig-like receptor 1
Klra22	**93969**	**-0.75**	-0.51	killer cell lectin-like receptor subfamily A, member 22
IGHV1S132	**NA**	**-0.75**	2.1	Mus musculus clone J558.h immunoglobulin heavy chain variable region gene, partial cds.
Hpgd	**15446**	**-0.75**	3.78	hydroxyprostaglandin dehydrogenase 15 (NAD)
Gpr3	**14748**	**-0.75**	0.73	G-protein coupled receptor 3
Esam1	**69524**	**-0.75**	4.4	endothelial cell-specific adhesion molecule
Ear3	**53876**	**-0.75**	1.2	eosinophil-associated, ribonuclease A family, member 3
Dok1	**13448**	**-0.75**	2.92	docking protein 1
Btbd4	**72147**	**-0.75**	5.43	BTB (POZ) domain containing 4
A530090P03Rik	**320407**	**-0.75**	5.36	RIKEN cDNA A530090P03 gene
6430402L03Rik	**331474**	**-0.75**	9.22	RIKEN cDNA 6430402L03 gene
1810037B05Rik	**69169**	**-0.75**	12.99	RIKEN cDNA 1810037B05 gene
Tnni2	**21953**	**-0.74**	10.31	troponin I, skeletal, fast 2
Syngr2	**20973**	**-0.74**	6.07	synaptogyrin 2
Samd7	**75953**	**-0.74**	3.2	sterile alpha motif domain containing 7
Rgs2	**19735**	**-0.74**	7.88	regulator of G-protein signaling 2
Pbxip1	**229534**	**-0.74**	-4.38	pre-B-cell leukemia transcription factor interacting protein 1
Nav1	**215690**	**-0.74**	8.29	neuron navigator 1
Napsa	**16541**	**-0.74**	12.31	napsin A aspartic peptidase
IGHV1S33	**NA**	**-0.74**	0.96	Mouse germline immunoglobulin V(H)II gene H13-1.
Icsbp1	**15900**	**-0.74**	4.16	interferon consensus sequence binding protein 1
Cyfip1	**20430**	**-0.74**	14.54	cytoplasmic FMR1 interacting protein 1
C330023F11Rik	**109232**	**-0.74**	7.6	RIKEN cDNA C330023F11 gene
B130050K08	**224180**	**-0.74**	8.4	hypothetical protein B130050K08
Alox5ap	**11690**	**-0.74**	6.53	arachidonate 5-lipoxygenase activating protein
6530404N21Rik	**67795**	**-0.74**	1.19	RIKEN cDNA 6530404N21 gene
1110060M21Rik	**66208**	**-0.74**	6.67	RIKEN cDNA 1110060M21 gene
Tcrg-V4	**21638**	**-0.73**	2.71	T-cell receptor gamma, variable 4
Snx8	**231834**	**-0.73**	6.09	sorting nexin 8
Slc2a6	**227659**	**-0.73**	10.84	solute carrier family 2 (facilitated glucose transporter), member 6
Rgl1	**19731**	**-0.73**	7.35	ral guanine nucleotide dissociation stimulator,-like 1
Mlp	**17357**	**-0.73**	13.61	MARCKS-like protein
Lyn	**17096**	**-0.73**	6.32	Yamaguchi sarcoma viral (v-yes-1) oncogene homolog
Klra1	**16627**	**-0.73**	0.1	killer cell lectin-like receptor, subfamily A, member 1
Gng10	**14700**	**-0.73**	6.78	guanine nucleotide binding protein (G protein), gamma 10
Gadd45b	**17873**	**-0.73**	11.79	growth arrest and DNA-damage-inducible 45 beta
Egr2	**13654**	**-0.73**	6.59	early growth response 2
Cnr2	**12802**	**-0.73**	9.43	cannabinoid receptor 2 (macrophage)
AA960558	**101476**	**-0.73**	8.38	expressed sequence AA960558
1810054O13Rik	**67893**	**-0.73**	7.4	RIKEN cDNA 1810054O13 gene
Tnfrsf5	**NA**	**-0.72**	9.28	Mus musculus tumor necrosis factor receptor superfamily, member 5 (Tnfrsf5), mRNA.
Tmprss8	**30943**	**-0.72**	18.92	transmembrane protease, serine 8 (intestinal)
Tlr7	**170743**	**-0.72**	4.46	toll-like receptor 7
Snn	**20621**	**-0.72**	4.98	stannin
P2ry6	**233571**	**-0.72**	13.74	pyrimidinergic receptor P2Y, G-protein coupled, 6
Hgfac	**54426**	**-0.72**	7.8	hepatocyte growth factor activator
Gpr126	**215798**	**-0.72**	3.68	G protein-coupled receptor 126
Egfl4	**269878**	**-0.72**	5.1	EGF-like-domain, multiple 4
D930007M16Rik	**319417**	**-0.72**	-0.86	RIKEN cDNA D930007M16 gene
Bcl2a1a	**12044**	**-0.72**	3.95	B-cell leukemia/lymphoma 2 related protein A1a
BC065078	**225912**	**-0.72**	6.29	cDNA sequence BC065078
A530023O14Rik	**244183**	**-0.72**	9.76	RIKEN cDNA A530023O14 gene
4933415L06Rik	**108811**	**-0.72**	2.49	RIKEN cDNA 4933415L06 gene
Tcrg-V2	**21636**	**-0.71**	-1.03	T-cell receptor gamma, variable 2
Syngr2	**20973**	**-0.71**	6.05	synaptogyrin 2
Spire1	**68166**	**-0.71**	9.62	spire homolog 1 (Drosophila)
Sh3tc1	**231147**	**-0.71**	7.63	SH3 domain and tetratricopeptide repeats 1
Sell	**20343**	**-0.71**	1.9	selectin, lymphocyte
Mefv	**54483**	**-0.71**	5.46	Mediterranean fever
Lair1	**52855**	**-0.71**	2.42	leukocyte-associated Ig-like receptor 1
Ii	**16149**	**-0.71**	0.51	Ia-associated invariant chain
Igk-V5	**381777**	**-0.71**	1.23	immunoglobulin kappa chain variable 5 (V5 family)
Icam1	**15894**	**-0.71**	10.31	intercellular adhesion molecule
Hoxa7	**15404**	**-0.71**	6.53	homeo box A7

Table S1B

Hk2	**15277**	**-0.71**	9.94	hexokinase 2
Edg2	**14745**	**-0.71**	4.12	endothelial differentiation, lysophosphatidic acid G-protein-coupled receptor, 2
D130019J16Rik	**320171**	**-0.71**	-0.61	RIKEN cDNA D130019J16 gene
C230090D14	**270198**	**-0.71**	3.16	hypothetical protein C230090D14
AW742481	**101013**	**-0.71**	9.71	expressed sequence AW742481
Atbf1	**11906**	**-0.71**	8.37	AT motif binding factor 1
9930027N05Rik	**NA**	**-0.71**	7.18	Mus musculus RIKEN cDNA 9930027N05 gene (9930027N05Rik), transcript variant 1, mRNA.
5830436I19Rik	**319946**	**-0.71**	-0.85	RIKEN cDNA 5830436I19 gene
2410004L22Rik	**76478**	**-0.71**	5	RIKEN cDNA 2410004L22 gene
Tpd52	**21985**	**-0.7**	13.54	tumor protein D52
Shb	**230126**	**-0.7**	9.57	src homology 2 domain-containing transforming protein B
Pftk1	**18647**	**-0.7**	10.99	PFTAIRE protein kinase 1
Pfkfb3	**170768**	**-0.7**	10.55	6-phosphofructo-2-kinase/fructose-2,6-biphosphatase 3
Jag1	**16449**	**-0.7**	6.17	jagged 1
Il1r2	**16178**	**-0.7**	3.57	interleukin 1 receptor, type II
Ifitm3	**66141**	**-0.7**	7.12	interferon induced transmembrane protein 3
H2-Ab1	**14961**	**-0.7**	0.61	histocompatibility 2, class II antigen A, beta 1
Eif2ak4	**27103**	**-0.7**	9.45	eukaryotic translation initiation factor 2 alpha kinase 4
Dusp16	**70686**	**-0.7**	7.38	dual specificity phosphatase 16
Csen	**56461**	**-0.7**	10.31	calsenilin, presenilin binding protein, EF hand transcription factor
Cd79b	**15985**	**-0.7**	6.86	CD79B antigen
BC013667	**226856**	**-0.7**	7.7	cDNA sequence BC013667
AV216087	**211896**	**-0.7**	6.9	expressed sequence AV216087
Acvr1	**11477**	**-0.7**	2.28	activin A receptor, type 1
A230020G22Rik	**NA**	**-0.7**	4.23	Mus musculus RIKEN cDNA A230020G22 gene (A230020G22Rik), mRNA.
2900026A02Rik	**243219**	**-0.7**	11.06	RIKEN cDNA 2900026A02 gene
Stk32c	**57740**	**-0.69**	8.63	serine/threonine kinase 32C
Sh3bp2	**24055**	**-0.69**	10.02	SH3-domain binding protein 2
Scrg3	**20286**	**-0.69**	3.13	scrapie responsive gene 3
Scd1	**20249**	**-0.69**	3.92	stearoyl-Coenzyme A desaturase 1
Procr	**19124**	**-0.69**	-1.84	protein C receptor, endothelial
Ppp1r14a	**NA**	**-0.69**	6.42	Mus musculus protein phosphatase 1, regulatory (inhibitor) subunit 14A (Ppp1r14a), mRNA.
Mgmt	**17314**	**-0.69**	2.13	O-6-methylguanine-DNA methyltransferase
IGKV8-21	**NA**	**-0.69**	-2.86	Mus musculus V kappa 8-21 gene, partial.
Haao	**107766**	**-0.69**	9.12	3-hydroxyanthranilate 3,4-dioxygenase
Gga2	**74105**	**-0.69**	9.47	golgi associated, gamma adaptin ear containing, ARF binding protein 2
Dst	**NA**	**-0.69**	2.32	Mus musculus dystonin (Dst), mRNA.
Btbd4	**72147**	**-0.69**	10.07	BTB (POZ) domain containing 4
Brunol4	**108013**	**-0.69**	9.02	bruno-like 4, RNA binding protein (Drosophila)
AL024069	**98496**	**-0.69**	15.99	expressed sequence AL024069
A430106J12Rik	**NA**	**-0.69**	10.93	Mus musculus RIKEN cDNA A430106J12 gene (A430106J12Rik), mRNA.
1100001I19Rik	**244745**	**-0.69**	5.62	RIKEN cDNA 1100001I19 gene
Zfp36l1	**12192**	**-0.68**	12.05	zinc finger protein 36, C3H type-like 1
Uvrag1	**78610**	**-0.68**	2.01	UV radiation resistance associated gene
Ugt1a13	**394430**	**-0.68**	10.94	UDP glycosyltransferase 1 family polypeptide A13
Slc38a2	**67760**	**-0.68**	11.84	solute carrier family 38, member 2
Relb	**19698**	**-0.68**	7.23	avian reticuloendotheliosis viral (v-rel) oncogene related B
Plac8	**231507**	**-0.68**	0.64	placenta-specific 8
Hps5	**246694**	**-0.68**	5.64	Hermansky-Pudlak syndrome 5 homolog (human)
Fcer2a	**14128**	**-0.68**	10.69	Fc receptor, IgE, low affinity II, alpha polypeptide
Dscam	**13508**	**-0.68**	15.14	Down syndrome cell adhesion molecule
Cri1	**58521**	**-0.68**	4.35	CREBBP/EP300 inhibitory protein 1
BC032935	**270103**	**-0.68**	1.87	cDNA sequence BC032935
4631426J05Rik	**77590**	**-0.68**	8.94	RIKEN cDNA 4631426J05 gene
1110012O05Rik	**66158**	**-0.68**	11.37	RIKEN cDNA 1110012O05 gene
Zfp238	**30928**	**-0.67**	2.17	zinc finger protein 238
Slco5a1	**240726**	**-0.67**	2.8	solute carrier organic anion transporter family, member 5A1
Sall2	**50524**	**-0.67**	7.49	sal-like 2 (Drosophila)
Ryr1	**20190**	**-0.67**	9.41	ryanodine receptor 1, skeletal muscle
Rassf4	**213391**	**-0.67**	11.33	Ras association (RalGDS/AF-6) domain family 4
LOC73899	**73899**	**-0.67**	5.75	hypothetical LOC73899
Klra21	**93968**	**-0.67**	0.77	killer cell lectin-like receptor subfamily A, member 21
Keo4	**226144**	**-0.67**	8.46	similar to Caenorhabditis elegans protein C42C1.9
Ear6	**93719**	**-0.67**	0.03	eosinophil-associated, ribonuclease A family, member 6
Ctsz	**64138**	**-0.67**	6.1	cathepsin Z
AI115600	**102442**	**-0.67**	2.93	expressed sequence AI115600
A230020G22Rik	**229499**	**-0.67**	12.35	RIKEN cDNA A230020G22 gene
2900076A13Rik	**73002**	**-0.67**	7.13	RIKEN cDNA 2900076A13 gene
Upb1	**103149**	**-0.66**	5.96	ureidopropionase, beta
Sart2	**212898**	**-0.66**	4.49	squamous cell carcinoma antigen recognized by T cells 2
Samsn1	**67742**	**-0.66**	3.18	SAM domain, SH3 domain and nuclear localisation signals, 1
Prkcd	**18753**	**-0.66**	7.27	protein kinase C, delta
Lst1	**16988**	**-0.66**	2.94	leukocyte specific transcript 1
Klrd1	**16643**	**-0.66**	1.88	killer cell lectin-like receptor, subfamily D, member 1
Il10ra	**16154**	**-0.66**	3.97	interleukin 10 receptor, alpha
Hist1h2bc	**68024**	**-0.66**	4.99	histone 1, H2bc
Gpr83	**14608**	**-0.66**	5.97	G protein-coupled receptor 83
Gkap1	**56278**	**-0.66**	5.59	G kinase anchoring protein 1
Entpd1	**12495**	**-0.66**	7.07	ectonucleoside triphosphate diphosphohydrolase 1
Ctla4	**12477**	**-0.66**	1.71	cytotoxic T-lymphocyte-associated protein 4
Cst3	**13010**	**-0.66**	5.94	cystatin C
Cd2ap	**12488**	**-0.66**	10.26	CD2-associated protein
C80638	**97086**	**-0.66**	0.98	expressed sequence C80638
C030044C12Rik	**77483**	**-0.66**	2.91	RIKEN cDNA C030044C12 gene
Aif1	**11629**	**-0.66**	2.13	allograft inflammatory factor 1
9130403I23Rik	**71585**	**-0.66**	2.56	RIKEN cDNA 9130403I23 gene
5830462O15Rik	**76110**	**-0.66**	-1.62	RIKEN cDNA 5830462O15 gene
4921501M20Rik	**74039**	**-0.66**	6.1	RIKEN cDNA 4921501M20 gene
Zfp263	**74120**	**-0.65**	5.91	zinc finger protein 263
Sumf1	**58911**	**-0.65**	10.14	sulfatase modifying factor 1
Pira5	**18728**	**-0.65**	8.41	paired-Ig-like receptor A5
Myo1b	**17912**	**-0.65**	10.42	myosin IB

Table S1B

Mef2c	**17260**	**-0.65**	6.72	myocyte enhancer factor 2C
Lincr	**214854**	**-0.65**	9.95	lung-inducible neuralized-related C3HC4 RING domain protein
H2-Ob	**15002**	**-0.65**	3.17	histocompatibility 2, O region beta locus
Gngt2	**14710**	**-0.65**	5.01	guanine nucleotide binding protein (G protein), gamma transducing activity polypeptide 2
Col12a1	**12816**	**-0.65**	7.64	procollagen, type XII, alpha 1
Adssl1	**11565**	**-0.65**	5.66	adenylosuccinate synthetase like 1
A930041H05Rik	**77834**	**-0.65**	3.29	RIKEN cDNA A930041H05 gene
4933431N12Rik	**71310**	**-0.65**	11.1	RIKEN cDNA 4933431N12 gene
4833418N02Rik	**74597**	**-0.65**	7.14	RIKEN cDNA 4833418N02 gene
Tnfrsf5	**21939**	**-0.64**	10	tumor necrosis factor receptor superfamily, member 5
Rnf130	**59044**	**-0.64**	13.81	ring finger protein 130
Rassf4	**213391**	**-0.64**	7.55	Ras association (RalGDS/AF-6) domain family 4
Luc7l2	**192196**	**-0.64**	0.97	LUC7-like 2 (S. cerevisiae)
IGKV8-30	**NA**	**-0.64**	-1.99	Mus musculus IgVk 8-30 gene.
Igj	**16069**	**-0.64**	-2.95	immunoglobulin joining chain
Gns	**75612**	**-0.64**	8.08	glucosamine (N-acetyl)-6-sulfatase
Ephx1	**13849**	**-0.64**	2.57	epoxide hydrolase 1, microsomal
E130014J05Rik	**214133**	**-0.64**	6.49	RIKEN cDNA E130014J05 gene
Dusp16	**NA**	**-0.64**	8.33	Mus musculus dual specificity phosphatase 16 (Dusp16), mRNA.
D4st1	**72136**	**-0.64**	4.66	dermatan 4 sulfotransferase 1
5033403D15Rik	**240024**	**-0.64**	7.48	RIKEN cDNA 5033403D15 gene
Wbscr5	**56743**	**-0.63**	8.02	Williams-Beuren syndrome chromosome region 5 homolog (human)
Tex2	**21763**	**-0.63**	2.84	testis expressed gene 2
Soat1	**20652**	**-0.63**	6.72	sterol O-acyltransferase 1
Slc30a4	**22785**	**-0.63**	-0.64	solute carrier family 30 (zinc transporter), member 4
Rgs1	**50778**	**-0.63**	6.75	regulator of G-protein signaling 1
Ptger3	**NA**	**-0.63**	3.46	Mus musculus prostaglandin E receptor 3 (subtype EP3) (Ptger3), mRNA.
Pira6	**18729**	**-0.63**	3.76	paired-Ig-like receptor A6
Pira2	**18725**	**-0.63**	10.45	paired-Ig-like receptor A2
Nfkbiz	**80859**	**-0.63**	4.56	nuclear factor of kappa light polypeptide gene enhancer in B-cells inhibitor, zeta
Myadm	**50918**	**-0.63**	10.48	myeloid-associated differentiation marker
Man2b1	**17159**	**-0.63**	6.29	mannosidase 2, alpha B1
Hcph	**15170**	**-0.63**	1.8	hemopoietic cell phosphatase
Gria3	**53623**	**-0.63**	1.23	glutamate receptor, ionotropic, AMPA3 (alpha 3)
Gpr35	**64095**	**-0.63**	11.21	G protein-coupled receptor 35
Gpr34	**23890**	**-0.63**	2.66	G protein-coupled receptor 34
D17H6S56E-5	**110956**	**-0.63**	6.32	DNA segment, Chr 17, human D6S56E 5
Cox6a2	**12862**	**-0.63**	1.64	cytochrome c oxidase, subunit VI a, polypeptide 2
Cklfsf3	**68119**	**-0.63**	4.75	chemokine-like factor super family 3
Asahl	**67111**	**-0.63**	6.53	N-acylsphingosine amidohydrolase (acid ceramidase)-like
A230102O09Rik	**98354**	**-0.63**	2.48	RIKEN cDNA A230102O09 gene
4930429A08Rik	**74648**	**-0.63**	6.37	RIKEN cDNA 4930429A08 gene
4930403L05Rik	**67395**	**-0.63**	13.14	RIKEN cDNA 4930403L05 gene
4733401I05Rik	**70848**	**-0.63**	1.83	RIKEN cDNA 4733401I05 gene
2210406H18Rik	**70133**	**-0.63**	9.81	RIKEN cDNA 2210406H18 gene
Tgm2	**21817**	**-0.62**	4.73	transglutaminase 2, C polypeptide
Sox4	**20677**	**-0.62**	-0.91	SRY-box containing gene 4
Rnf13	**24017**	**-0.62**	11.55	ring finger protein 13
Ptpre	**19267**	**-0.62**	11.54	protein tyrosine phosphatase, receptor type, E
Parc	**78309**	**-0.62**	8.4	p53-associated parkin-like cytoplasmic protein
Napsa	**NA**	**-0.62**	0.4	Mus musculus napsin A aspartic peptidase (Napsa), mRNA.
Ms4a6c	**73656**	**-0.62**	7.18	membrane-spanning 4-domains, subfamily A, member 6C
Lrp1	**16971**	**-0.62**	6.56	low density lipoprotein receptor-related protein 1
Lmyc1	**16918**	**-0.62**	6.79	lung carcinoma myc related oncogene 1
Klk6	**16612**	**-0.62**	1.38	kallikrein 6
H2-Ab1	**14961**	**-0.62**	-0.44	histocompatibility 2, class II antigen A, beta 1
Gp2	**67133**	**-0.62**	7.76	glycoprotein 2 (zymogen granule membrane)
E130103I17Rik	**319845**	**-0.62**	8.92	RIKEN cDNA E130103I17 gene
D330017J20Rik	**320609**	**-0.62**	14.66	RIKEN cDNA D330017J20 gene
Cr2	**12902**	**-0.62**	6.79	complement receptor 2
Cdh1	**12550**	**-0.62**	-3.22	cadherin 1
Cd244	**18106**	**-0.62**	13.98	CD244 natural killer cell receptor 2B4
Cbr3	**109857**	**-0.62**	9.25	carbonyl reductase 3
Catna1	**12385**	**-0.62**	4.42	catenin alpha 1
BC027057	**212937**	**-0.62**	14.28	cDNA sequence BC027057
BC004728	**207818**	**-0.62**	8.84	cDNA sequence BC004728
Abi3	**66610**	**-0.62**	4.15	ABI gene family, member 3
Abca9	**217262**	**-0.62**	6.57	ATP-binding cassette transporter sub-family A member 9
9830005G06Rik	**232414**	**-0.62**	4.61	RIKEN cDNA 9830005G06 gene
9630007J19Rik	**102493**	**-0.62**	8.5	RIKEN cDNA 9630007J19 gene
2300003P22Rik	**67855**	**-0.62**	8.33	RIKEN cDNA 2300003P22 gene
Swap70	**20947**	**-0.61**	4.93	SWAP complex protein
Spint1	**20732**	**-0.61**	2.59	serine protease inhibitor, Kunitz type 1
Plxnb2	**140570**	**-0.61**	5.87	plexin B2
Pira3	**18726**	**-0.61**	9.13	paired-Ig-like receptor A3
Nav1	**215690**	**-0.61**	6.2	neuron navigator 1
Mtmr13	**319934**	**-0.61**	-0.26	myotubularin related protein 13
Kit	**16590**	**-0.61**	4.69	kit oncogene
Ifitm1	**68713**	**-0.61**	3.45	interferon induced transmembrane protein 1
Dusp9	**75590**	**-0.61**	2.93	dual specificity phosphatase 9
Cdkn2b	**12579**	**-0.61**	11.25	cyclin-dependent kinase inhibitor 2B (p15, inhibits CDK4)
Ccr5	**12774**	**-0.61**	2.15	chemokine (C-C motif) receptor 5
BC013481	**NA**	**-0.61**	0.53	Mus musculus cDNA sequence BC013481 (BC013481), mRNA.
Adam4	**11498**	**-0.61**	8.78	a disintegrin and metalloprotease domain 4
5830456J23Rik	**76105**	**-0.61**	2.49	RIKEN cDNA 5830456J23 gene
Tusc1	**69136**	**-0.6**	11.11	tumor suppressor candidate 1
Tnfrsf19l	**320100**	**-0.6**	11.43	tumor necrosis factor receptor superfamily, member 19-like
Stx7	**53331**	**-0.6**	3.44	syntaxin 7
Soat1	**20652**	**-0.6**	3.86	sterol O-acyltransferase 1
Sgk3	**170755**	**-0.6**	4.55	serum/glucocorticoid regulated kinase 3
Rfx5	**53970**	**-0.6**	5.81	regulatory factor X, 5 (influences HLA class II expression)
Prkcb1	**18751**	**-0.6**	8.56	protein kinase C, beta 1

Table S1B

Plek	**56193**	**-0.6**	10.67	pleckstrin
Pira7	**18730**	**-0.6**	7.03	paired-Ig-like receptor A7
Pglyrp1	**21946**	**-0.6**	0.93	peptidoglycan recognition protein 1
Pacsin1	**23969**	**-0.6**	3.62	protein kinase C and casein kinase substrate in neurons 1
Myb	**17863**	**-0.6**	-1.08	myeloblastosis oncogene
Mmp9	**17395**	**-0.6**	2.21	matrix metalloproteinase 9
Mmd	**67468**	**-0.6**	2.12	monocyte to macrophage differentiation-associated
Malt1	**240354**	**-0.6**	3.58	mucosa associated lymphoid tissue lymphoma translocation gene 1
Lyl1	**17095**	**-0.6**	10.38	lymphoblastomic leukemia
Klra17	**170733**	**-0.6**	4.14	killer cell lectin-like receptor, subfamily A, member 17
Igk-V8	**384422**	**-0.6**	-4.7	immunoglobulin kappa chain variable 8 (V8)
Grn	**14824**	**-0.6**	0.85	granulin
Gata4	**14463**	**-0.6**	5.78	GATA binding protein 4
Dfy	**13349**	**-0.6**	5.32	Duffy blood group
Chst7	**60322**	**-0.6**	5.89	carbohydrate (N-acetylglucosamino) sulfotransferase 7
BC028528	**229600**	**-0.6**	4.47	cDNA sequence BC028528
Asah1	**11886**	**-0.6**	9.6	N-acylsphingosine amidohydrolase 1
A530023O14Rik	**244183**	**-0.6**	10.02	RIKEN cDNA A530023O14 gene
A030011A13Rik	**319515**	**-0.6**	2.08	RIKEN cDNA A030011A13 gene
6530401C20Rik	**231842**	**-0.6**	9.71	RIKEN cDNA 6530401C20 gene
4631422O05Rik	**78749**	**-0.6**	5.44	RIKEN cDNA 4631422O05 gene
1810009J06Rik	**73626**	**-0.6**	9.79	RIKEN cDNA 1810009J06 gene
Tfb2m	**15278**	**-0.59**	6.46	transcription factor B2, mitochondrial
Tceal8	**66684**	**-0.59**	9.14	transcription elongation factor A (SII)-like 8
Rel	**19696**	**-0.59**	8.63	reticuloendotheliosis oncogene
Pdcd1lg1	**60533**	**-0.59**	9.36	programmed cell death 1 ligand 1
Nalp10	**244202**	**-0.59**	6.1	NACHT, leucine rich repeat and PYD containing 10
Man2b1	**17159**	**-0.59**	3.87	mannosidase 2, alpha B1
LOC56304	**56304**	**-0.59**	-0.68	recombinant antineuraminidase single chain Ig VH and VL domains
L259	**NA**	**-0.59**	7.43	Mus musculus L259 (L259), mRNA.
Klk5	**16622**	**-0.59**	1.74	kallikrein 5
IGKV1-115	**NA**	**-0.59**	0.37	Mus musculus IgVk cz1 pseudogene.
Igk-V1	**16081**	**-0.59**	3.29	immunoglobulin kappa chain variable 1 (V1)
Ifi30	**NA**	**-0.59**	3.34	Mus musculus interferon gamma inducible protein 30 (Ifi30), mRNA.
Hspa5bp1	**98170**	**-0.59**	10.92	heat shock 70kDa protein 5 binding protein 1
Fut4	**14345**	**-0.59**	7.78	fucosyltransferase 4
Entpd1	**12495**	**-0.59**	9.05	ectonucleoside triphosphate diphosphohydrolase 1
Dusp22	**105352**	**-0.59**	12.56	dual specificity phosphatase 22
Cyp4f18	**72054**	**-0.59**	5.49	cytochrome P450, family 4, subfamily f, polypeptide 18
Cyp4f16	**70101**	**-0.59**	1.5	cytochrome P450, family 4, subfamily f, polypeptide 16
A630038E17Rik	**219065**	**-0.59**	4.88	RIKEN cDNA A630038E17 gene
9530009G21Rik	**320305**	**-0.59**	3.03	RIKEN cDNA 9530009G21 gene
6720401G13Rik	**103012**	**-0.59**	1.81	RIKEN cDNA 6720401G13 gene
6330415F13Rik	**70747**	**-0.59**	5.92	RIKEN cDNA 6330415F13 gene
5730496F10Rik	**70604**	**-0.59**	4.54	RIKEN cDNA 5730496F10 gene
4930527B16Rik	**75834**	**-0.59**	4.21	RIKEN cDNA 4930527B16 gene
2810465F10Rik	**78771**	**-0.59**	4.23	RIKEN cDNA 2810465F10 gene
Hsd11b1	**NA**	**0.59**	2.55	Mus musculus hydroxysteroid 11-beta dehydrogenase 1 (Hsd11b1), mRNA.
H2-Q10	**15007**	**0.59**	1.2	histocompatibility 2, Q region locus 10
Gzmk	**14945**	**0.59**	-3.67	granzyme K
Golph2	**105348**	**0.59**	11.99	golgi phosphoprotein 2
Entpd5	**12499**	**0.59**	5.22	ectonucleoside triphosphate diphosphohydrolase 5
Eef2	**NA**	**0.59**	5.2	Mus musculus eukaryotic translation elongation factor 2 (Eef2), mRNA.
Eef2	**13629**	**0.59**	3.84	eukaryotic translation elongation factor 2
Cspg6	**13006**	**0.59**	0.56	chondroitin sulfate proteoglycan 6
Cd3z	**12503**	**0.59**	5.47	CD3 antigen, zeta polypeptide
Art4	**109978**	**0.59**	8.93	ADP-ribosyltransferase 4
A130096G17Rik	**319725**	**0.59**	1.46	RIKEN cDNA A130096G17 gene
Ubash3a	**328795**	**0.6**	1.34	ubiquitin associated and SH3 domain containing, A
Tmem23	**208449**	**0.6**	5.44	transmembrane protein 23
Prkx	**19108**	**0.6**	2.49	protein kinase, X-linked
Lrrc6	**54562**	**0.6**	-0.27	leucine rich repeat containing 6 (testis)
Gp49a	**14727**	**0.6**	6.11	glycoprotein 49 A
Cd28	**NA**	**0.6**	5.97	Mus musculus CD28 antigen (Cd28), mRNA.
Capn2	**12334**	**0.6**	5.52	calpain 2
Bcl2	**NA**	**0.6**	2.45	Mus musculus B-cell leukemia/lymphoma 2 (Bcl2), mRNA.
Vim	**NA**	**0.61**	4.69	Mus musculus vimentin (Vim), mRNA.
Vim	**NA**	**0.61**	3.69	Mus musculus vimentin (Vim), mRNA.
Vim	**22352**	**0.61**	0.36	vimentin
Stk38	**106504**	**0.61**	4.71	serine/threonine kinase 38
Slc25a24	**229731**	**0.61**	2.15	solute carrier family 25 (mitochondrial carrier, phosphate carrier), member 24
Rbl2	**NA**	**0.61**	2.84	Mus musculus retinoblastoma-like 2 (Rbl2), mRNA.
Prkx	**19108**	**0.61**	8.2	protein kinase, X-linked
Hadhsc	**15107**	**0.61**	4	L-3-hydroxyacyl-Coenzyme A dehydrogenase, short chain
AI414495	**NA**	**0.61**	3.8	Mus musculus dickkopf-like 1 (Dkkl1), mRNA.
1110032D12Rik	**56334**	**0.61**	0.8	RIKEN cDNA 1110032D12 gene
Trim10	**19824**	**0.62**	-2.37	tripartite motif protein 10
Spnb2	**NA**	**0.62**	2.97	Mus musculus spectrin beta 2 (Spnb2), transcript variant 1, mRNA.
Rasgrp1	**19419**	**0.62**	3.99	RAS guanyl releasing protein 1
Pgm2l1	**70974**	**0.62**	3.13	phosphoglucomutase 2-like 1
Pdlim1	**54132**	**0.62**	3.13	PDZ and LIM domain 1 (elfin)
Eif4a2	**NA**	**0.62**	3.26	Mus musculus eukaryotic translation initiation factor 4A2 (Eif4a2), mRNA.
Dnajc6	**72685**	**0.62**	8.59	DnaJ (Hsp40) homolog, subfamily C, member 6
BC006662	**223267**	**0.62**	5.73	cDNA sequence BC006662
TRAV14D-2	**NA**	**0.63**	6.31	Mus musculus BALB/c T-cell receptor alpha chain (Va2 family) gene, exons 1 and 2, partial cds.
Nasp	**NA**	**0.63**	1.37	Mus musculus nuclear autoantigenic sperm protein (histone-binding) (Nasp), mRNA.
Lgals9	**NA**	**0.63**	5.16	Mus musculus lectin, galactose binding, soluble 9 (Lgals9), mRNA.
Il22	**50929**	**0.63**	9.86	interleukin 22
Cdc25b	**12531**	**0.63**	10.26	cell division cycle 25 homolog B (S. cerevisiae)
Btg1	**12226**	**0.63**	0.89	B-cell translocation gene 1, anti-proliferative
6330500D04Rik	**193385**	**0.63**	3.41	RIKEN cDNA 6330500D04 gene
2610027H17Rik	**71811**	**0.63**	-5.88	RIKEN cDNA 2610027H17 gene

Table S1B

2010002M12Rik	**112419**	**0.63**	2.49	RIKEN cDNA 2010002M12 gene
Psip1	**NA**	**0.64**	2.67	Mus musculus PC4 and SFRS1 interacting protein 1 (Psip1), mRNA.
Il9r	**16199**	**0.64**	4.64	interleukin 9 receptor
Il23r	**209590**	**0.64**	12.16	interleukin 23 receptor
Capn2	**12334**	**0.64**	0.69	calpain 2
Btg1	**12226**	**0.64**	0.71	B-cell translocation gene 1, anti-proliferative
TRAV12D-3	**NA**	**0.65**	4.42	Mouse germ line TCR V -alpha F3.3 gene.
Tcrb-V8.2	**21607**	**0.65**	5.63	T-cell receptor beta, variable 8.2
Arl7	**320982**	**0.65**	9.04	ADP-ribosylation factor-like 7
2610204G22Rik	**70448**	**0.65**	5.08	RIKEN cDNA 2610204G22 gene
2310004K06Rik	**NA**	**0.65**	7.29	Mus musculus RIKEN cDNA 2310004K06 gene (2310004K06Rik), mRNA.
1110034G24Rik	**73747**	**0.65**	9.31	RIKEN cDNA 1110034G24 gene
TRBV15	**NA**	**0.66**	-0.14	Mus musculus TCR beta locus from bases 250554 to 501917 (section 2 of 3) of the complete sequence.
TRAV7D-3	**NA**	**0.66**	4.11	Mouse germline gene for T-cell receptor V-alpha 5H.
TRAV12D-2	**NA**	**0.66**	4.21	Mouse germline TCR V-alpha F3.2 gene.
Sgk	**20393**	**0.66**	4.18	serum/glucocorticoid regulated kinase
Rasa3	**19414**	**0.66**	10	RAS p21 protein activator 3
Mrps5	**NA**	**0.66**	7.83	Mitochondrial 28S ribosomal protein S5 (MRP-S5).
Edg1	**13609**	**0.66**	3.13	endothelial differentiation sphingolipid G-protein-coupled receptor 1
Dnaja1	**NA**	**0.66**	1.67	Mus musculus DnaJ (Hsp40) homolog, subfamily A, member 1 (Dnaja1), mRNA.
Actr10	**NA**	**0.66**	4.41	Mus musculus ARP10 actin-related protein 10 homolog (S. cerevisiae) (Actr10), mRNA.
Slc43a1	**72401**	**0.67**	7.62	solute carrier family 43, member 1
Rasgrp1	**19419**	**0.67**	0.57	RAS guanyl releasing protein 1
Lpd	**94180**	**0.67**	12.98	lipidosin
6330500D04Rik	**193385**	**0.67**	3.06	RIKEN cDNA 6330500D04 gene
Tnfrsf19	**29820**	**0.68**	0.94	tumor necrosis factor receptor superfamily, member 19
Stat4	**20849**	**0.68**	4.76	signal transducer and activator of transcription 4
Pitpnc1	**71795**	**0.68**	4.33	phosphatidylinositol transfer protein, cytoplasmic 1
Itga4	**16401**	**0.68**	1.6	integrin alpha 4
Hivep2	**15273**	**0.68**	4.21	human immunodeficiency virus type I enhancer binding protein 2
9330161A08Rik	**320112**	**0.68**	-3.06	RIKEN cDNA 9330161A08 gene
6720435I21Rik	**77734**	**0.68**	4.04	RIKEN cDNA 6720435I21 gene
2210013M04Rik	**329384**	**0.68**	8.91	RIKEN cDNA 2210013M04 gene
Sh3kbp1	**NA**	**0.69**	5.64	Mus musculus SH3-domain kinase binding protein 1 (Sh3kbp1), mRNA.
Myo6	**17920**	**0.69**	0.93	myosin VI
IGHG1	**NA**	**0.69**	6.92	Mouse Ig germline D-J-C region alpha gene and secreted tail.
Hlf	**217082**	**0.69**	-0.1	hepatic leukemia factor
Ass1	**NA**	**0.69**	8.72	Mus musculus argininosuccinate synthetase 1 (Ass1), mRNA.
Apobec2	**11811**	**0.69**	-1.32	apolipoprotein B editing complex 2
Spnb2	**NA**	**0.7**	4.77	Mus musculus spectrin beta 2 (Spnb2), transcript variant 1, mRNA.
LOC381240	**381240**	**0.7**	5.15	similar to calcium and DAG-regulated guanine nucleotide exchange factor I
Lef1	**16842**	**0.7**	9.27	lymphoid enhancer binding factor 1
Eef2	**NA**	**0.7**	4.54	Mus musculus eukaryotic translation elongation factor 2 (Eef2), mRNA.
Adk	**NA**	**0.7**	9.21	Mus musculus adenosine kinase (Adk), mRNA.
Vim	**NA**	**0.71**	6.09	Mus musculus vimentin (Vim), mRNA.
Nckap1	**50884**	**0.71**	4.52	NCK-associated protein 1
AW320017	**103030**	**0.71**	6.73	expressed sequence AW320017
TRAV7D-2	**NA**	**0.72**	5.12	Mus musculus T-cell receptor alpha locus BAC clone MBAC01 from 14D1-D2, complete sequence.
Scotin	**NA**	**0.72**	3.19	Mus musculus scotin gene (Scotin), mRNA.
Rnf125	**67664**	**0.72**	10.06	ring finger protein 125
Il7r	**16197**	**0.72**	11.71	interleukin 7 receptor
Hrh4	**225192**	**0.72**	8.16	histamine H4 receptor
E430024C06Rik	**319443**	**0.72**	-1.76	RIKEN cDNA E430024C06 gene
Dnajc6	**72685**	**0.72**	6.54	DnaJ (Hsp40) homolog, subfamily C, member 6
BC022645	**235633**	**0.72**	2.01	cDNA sequence BC022645
Anapc5	**NA**	**0.72**	8.91	Mus musculus anaphase-promoting complex subunit 5 (Anapc5), mRNA.
1500005K14Rik	**76566**	**0.72**	4.46	RIKEN cDNA 1500005K14 gene
Wdr12	**57750**	**0.73**	8.71	WD repeat domain 12
Rora	**19883**	**0.73**	6.13	RAR-related orphan receptor alpha
Klf2	**16598**	**0.73**	4.34	Kruppel-like factor 2 (lung)
5730469M10Rik	**70564**	**0.73**	11.66	RIKEN cDNA 5730469M10 gene
Grap2	**17444**	**0.75**	10.31	GRB2-related adaptor protein 2
Igh-4	**16017**	**0.76**	6.85	immunoglobulin heavy chain 4 (serum IgG1)
6330500D04Rik	**193385**	**0.76**	5.34	RIKEN cDNA 6330500D04 gene
4933439K08Rik	**71223**	**0.76**	2.67	RIKEN cDNA 4933439K08 gene
TRBV3	**NA**	**0.77**	4.28	Mus musculus TCR beta locus from bases 1 to 250611 (section 1 of 3) of the complete sequence.
Socs3	**12702**	**0.77**	2.8	suppressor of cytokine signaling 3
Eraf	**170812**	**0.77**	2.15	erythroid associated factor
9030406N13Rik	**211329**	**0.77**	4.58	RIKEN cDNA 9030406N13 gene
Il18r1	**16182**	**0.78**	3.71	interleukin 18 receptor 1
Sh3kbp1	**58194**	**0.79**	5.52	SH3-domain kinase binding protein 1
Crip1	**12925**	**0.79**	8.37	cysteine-rich protein 1 (intestinal)
Vim	**NA**	**0.8**	8.82	Mus musculus vimentin (Vim), mRNA.
Vim	**NA**	**0.8**	8.6	Mus musculus vimentin (Vim), mRNA.
TRBV26	**NA**	**0.8**	3.09	Mouse T-cell receptor beta-chain germline V-region gene.
Sytl3	**83672**	**0.8**	7.75	synaptotagmin-like 3
Smc4l1	**70099**	**0.8**	3.85	SMC4 structural maintenance of chromosomes 4-like 1 (yeast)
Cd96	**84544**	**0.8**	6.27	CD96 antigen
Rnf125	**67664**	**0.81**	6.4	ring finger protein 125
Ltb4r1	**16995**	**0.81**	13.05	leukotriene B4 receptor 1
Vim	**NA**	**0.82**	7.08	Mus musculus vimentin (Vim), mRNA.
Slc16a5	**217316**	**0.83**	8.92	solute carrier family 16 (monocarboxylic acid transporters), member 5
Glipr2	**384009**	**0.83**	7.81	GLI pathogenesis-related 2
Capn2	**12334**	**0.83**	7.78	calpain 2
Atp10a	**11982**	**0.83**	6.56	ATPase, class V, type 10A
Il7r	**16197**	**0.84**	7.44	interleukin 7 receptor
Eif4g2	**NA**	**0.84**	0.92	Mus musculus eukaryotic translation initiation factor 4, gamma 2 (Eif4g2), mRNA.
A130092J06Rik	**241303**	**0.84**	5.57	RIKEN cDNA A130092J06 gene
1200015M12Rik	**71739**	**0.85**	8.2	RIKEN cDNA 1200015M12 gene
Prss12	**19142**	**0.86**	17.38	protease, serine, 12 neurotrypsin (motopsin)
Ifit1	**15957**	**0.87**	-1.98	interferon-induced protein with tetratricopeptide repeats 1
Tcrim	**77647**	**0.88**	9.39	T-cell receptor interacting molecule

Table S1B

Mal	**17153**	**0.88**	6.12	myelin and lymphocyte protein, T-cell differentiation protein
Atp1b3	**11933**	**0.88**	13	ATPase, Na+/K+ transporting, beta 3 polypeptide
Lef1	**16842**	**0.89**	6.54	lymphoid enhancer binding factor 1
Klhl6	**239743**	**0.9**	5.3	kelch-like 6 (Drosophila)
Il1r1	**16177**	**0.94**	6.24	interleukin 1 receptor, type I
Tcrb-J	**21580**	**0.95**	11.85	T-cell receptor beta, joining region
2610019F03Rik	**72148**	**0.95**	17.81	RIKEN cDNA 2610019F03 gene
TRBV31	**NA**	**0.96**	9.47	Mouse germline T-cell receptor V-beta 14 gene segment 10 kb 3' to C-beta 2 gene.
Trib2	**217410**	**0.98**	15.66	tribbles homolog 2 (Drosophila)
Tcf7	**21414**	**0.98**	11.15	transcription factor 7, T-cell specific
Slc22a4	**30805**	**0.98**	6.33	solute carrier family 22 (organic cation transporter), member 4
AI661017	**213068**	**0.98**	13.84	expressed sequence AI661017
1110060I01Rik	**66207**	**0.98**	6.78	RIKEN cDNA 1110060I01 gene
TRBV6	**NA**	**1.01**	8.56	Mus musculus TCR beta locus from bases 1 to 250611 (section 1 of 3) of the complete sequence.
Il17re	**57890**	**1.01**	19.4	interleukin 17 receptor E
2610304F08Rik	**70468**	**1.02**	3.54	RIKEN cDNA 2610304F08 gene
E430004N04Rik	**210757**	**1.05**	12.42	RIKEN cDNA E430004N04 gene
TRBV1	**NA**	**1.06**	5.12	Mus musculus TCR beta locus from bases 1 to 250611 (section 1 of 3) of the complete sequence.
Dnajd1	**66148**	**1.06**	6.53	DnaJ (Hsp40) homolog, subfamily D, member 1
1810045K07Rik	**NA**	**1.06**	12	Mus musculus RIKEN cDNA 1810045K07 gene (1810045K07Rik), mRNA.
Actn2	**11472**	**1.08**	16.34	actinin alpha 2
Ggt1	**14598**	**1.1**	3.92	gamma-glutamyltransferase 1
Nsg2	**18197**	**1.11**	8.84	neuron specific gene family member 2
5830431A10Rik	**76024**	**1.13**	8.73	RIKEN cDNA 5830431A10 gene
Pparg	**19016**	**1.17**	12.8	peroxisome proliferator activated receptor gamma
C030046M14Rik	**NA**	**1.18**	12.04	Mus musculus RIKEN cDNA C030046M14 gene (C030046M14Rik), mRNA.
Morc	**17450**	**1.2**	14.59	microrchidia
Cpm	**70574**	**1.22**	8.28	carboxypeptidase M
Ptgir	**19222**	**1.28**	14.74	prostaglandin I receptor (IP)
Speer6-ps1	**73266**	**1.34**	11.56	spermatogenesis associated glutamate (E)-rich protein 6, pseudogene 1
Tcrim	**77647**	**1.38**	15.7	T-cell receptor interacting molecule
Gzma	**14938**	**1.42**	-1.07	granzyme A
Pdlim4	**30794**	**1.44**	15.11	PDZ and LIM domain 4
Emp1	**13730**	**1.68**	12.36	epithelial membrane protein 1
Speer1-ps1	**70896**	**1.77**	21.13	spermatogenesis associated glutamate (E)-rich protein 1, pseudogene 1
