# Supplementary material for: Impact of the TCR Signal on Regulatory T Cell Homeostasis, Function, and Trafficking
Source: PLoS One. 2009 Aug 11;4(8):e6580. doi: 10.1371/journal.pone.0006580 (PMC2719063; doi:10.1371/journal.pone.0006580)
Supplement: Table S2 — Gene ontology term analysis of differentially expressed genes in memory and regulatory T cells with inactivated p56Lck. Genes identified as differentially expressed (>1.5 fold fold-change and B statistic>0) in p56Lck-deficient cells compared to control cells were analyzed for enriched gene ontology (GO) annotation terms relative to the entire mouse genome using the DAVID Functional Annotation Tools [81] (April 2008 release). p-values for enrichment were obtained using a modified Fisher Exact test with adjustment for multiple sampling (Bonferroni). The table shows biological process GO terms (greater than level 1) associated with p-values less than 0.05; terms referring to related functions were grouped then ranked according to fold change (degree of over-representation of the term in the query list relative to representation in the mouse genome). (0.05 MB PDF) [file pone.0006580.s004.pdf]

Table S2

Regulatory

T cell activation	**GO ID**	**Fold Enrichment**
Lymphocyte activation	0046649	4.6
Leukocyte activation	0045321	4.6
Cell activation	0001775	4.2
Apoptosis		
Death	0016265	2.2
Signaling / Immune Response / Differentiation / Development		
Regulation of cell differentiation	0045595	4.6
Hemopoietic or lymphoid organ development	0048534	3.8
Immune system development	0002520	3.7
Regulation of developmental process	0050793	3.6
Immune response	0006955	3.2

Memory

T cell activation	**GO ID**	**Fold Enrichment**
Regulation of lymphocyte activation	0051249	4.6
Regulation of cell activation	0050865	4.7
Lymphocyte activation	0046649	4.2
T cell activation	0042110	3.2
Leukocyte activation	0045321	4.4
Proliferation/Cell Cycle		
Lymphocyte proliferation	0046651	4.6
Mononuclear cell proliferation	0032943	4.6
Cell proliferation	0008283	1.9
Apoptosis		
Cell death	0008219	1.9
Death	0016265	1.8
Signaling / Immune Response / Differentiation / Development		
Immune response-regulating cell surface receptor signaling pathway	0002768	9.3
Antigen receptor-mediated signaling pathway	0050851	7.9
Immune response-activating cell surface receptor signaling pathway	0002429	8.6
Immune response-regulating signal transduction	0002764	8.2
Immune response-activating signal transduction	0002757	7.9
