# Supplementary material for: Impact of the TCR Signal on Regulatory T Cell Homeostasis, Function, and Trafficking
Source: PLoS One. 2009 Aug 11;4(8):e6580. doi: 10.1371/journal.pone.0006580 (PMC2719063; doi:10.1371/journal.pone.0006580)
Supplement: Table S3 — Gene ontology term analysis of differentially expressed genes in memory and regulatory T cells with inactivated p56Lck. Differentially expressed genes (fold change>1.5) were grouped according to their location in the plot shown in Figure 6B. Quadrants in the figure were labeled sequentially from upper right (q1) clockwise to upper left (q4) and the numbers of differentially expressed genes in each quadrant are shown in brackets at the top of the table. Enrichment of gene ontology terms was determined as in Table 1 except that adjustment for multiple sampling was only used for the analysis of genes in quadrant 3. (0.03 MB PDF) [file pone.0006580.s005.pdf]

Table S2

	GO Term ID	Fold Enrichment q1 (250)	q2 (118)	q3 (848)	q4 (60)
**Activation**	0002274 (8), 0051250 (8), 0050864 (9), 0042113 (19), 0051249 (18), 0050865 (18), 0051251 (12), 0050863 (13), 0045321 (36), 0001775 (38), 0046649 (33), 0042110 (19)			4.2-7.9	
**Proliferation**	0042100 (8), 0032946 ((9), 0032944 (12), 0050670 (12), 0032943 (16), 0046651 (16), 0042098 (10), 0042127 (28), 0008283 (37), 0007049 (40)			1.7-8.5	
	0030261 (2), 0051301 (4), 0022403 (5), 0000278 (4), 0000279 (4), 0022402 (5)				3.8-41.3
**Apoptosis**	0008219 (13), 0016265 (13), 0006915 (12), 0012501 (12)	2.1-2.2			
	0016265 (47), 0042981 (30), 0008219 (47), 0006915 (42), 0012501 (42)			1.9-2.0	
**Signaling**	0009967 (13), 0051056 (17), 0009966 (39), 0050790 (30), 0007242 (78)			2.0-3.8	
	0019932 (5), 0006955 (17), 0007242 (23), 0016310 (13), 0048468 (17)	1.7-4.3			
**Immune Response**	0045087 (3), 0001817 (8), 0045619 (7), 0002768 (8), 0001816 (17), 0002443 (15), 0002429 (7), 0002250 (13), 0002460 (13), 0030098 (14), 0002449 (13), 0030099 (14), 0002521 (19), 0050776 (14), 0002252 (16), 0002682 (14), 0006954 (22), 0006955 (47), 0030154 (99)			1.7-13.4	
**Development**	0030097 (30), 0048534 (32), 0002520 (34), 0048468 (7), 0050793 (21), 0048869 (99), 0048468 (65), 0048731 (79)			1.4-3.8	
