# Supplementary material for: Impact of the TCR Signal on Regulatory T Cell Homeostasis, Function, and Trafficking
Source: PLoS One. 2009 Aug 11;4(8):e6580. doi: 10.1371/journal.pone.0006580 (PMC2719063; doi:10.1371/journal.pone.0006580)
Supplement: Table S4 — Treg signature genes affected by p56Lck inactivation. A. 362 genes common to the microarrays used in this study and those reported in Hill et al. [40]. B. 75 of the genes in Table S4A that showed>1.5 fold change in expression due to inactivation of p56Lck. 61 genes that are normally upregulated in Treg cells compared to conventional T cells are separated from 14 genes (at the bottom of the list) that are normally downregulated in Treg cells. 59 of the 61 genes were expressed at reduced levels in the absence of p56Lck than in its presence (i.e., the loss of p56Lck diminished the Treg signature); the two genes that showed the opposite behavior are listed in italics. All 14 genes that are normally downregulated in Treg cells showed increased expression in the absence of p56Lck than in its presence (i.e., again, the loss of p56Lck reduced the Treg signature). (0.13 MB PDF) [file pone.0006580.s006.pdf]

Table S4A

Symbol	Gene ID	Description	Treg Lck fold change (log$_2$)	Treg/Tconv signature (log$_2$)
Il2ra	16184	interleukin 2 receptor, alpha chain	-0.14	5.558
Foxp3	20371	forkhead box P3	-0.18	5.178
Plagl1	22634	pleiomorphic adenoma gene-like 1	-0.32	4.300
Itgb8	320910	integrin beta 8	-0.09	4.285
Socs2	216233	suppressor of cytokine signaling 2	-0.09	4.263
Dusp4	319520	dual specificity phosphatase 4	-1.3	3.621
4631408O11Rik	66693	RIKEN cDNA 4631408O11 gene	-0.79	3.392
Il2rb	16185	Interleukin 2 receptor, beta chain	-0.35	3.336
Ctla4	12477	cytotoxic T-lymphocyte-associated protein 4	-0.62	3.202
Gpr83	14608	G protein-coupled receptor 83	-0.39	3.104
Zfpn1a4	22781	zinc finger protein, subfamily 1A, 4 (Eos)	-0.09	3.104
Itih5	209378	inter-alpha (globulin) inhibitor H5	-0.44	3.018
A730095J18Rik	319462	RIKEN cDNA A730095J18 gene	0.04	2.963
Zfpn1a2	22779	zinc finger protein, subfamily 1A, 2 (Helios)	-0.79	2.848
Rgs16	19734	regulator of G-protein signaling 16	-0.32	2.828
Nrp1	18186	neuropilin 1	-0.83	2.807
Tiam1	21844	T-cell lymphoma invasion and metastasis 1	-1.1	2.766
Hmgn3	94353	high mobility group nucleosomal binding domain 3	-1.05	2.678
Itgae	16407	integrin, alpha E, epithelial-associated	-0.9	2.609
Prnp	19122	prion protein	0.03	2.536
Ptger2	19217	prostaglandin E receptor 2 (subtype EP2)	-1.06	2.511
Rgs1	50778	regulator of G-protein signaling 1	-0.11	2.459
Myo9a	272606	Myosin IXa	0.04	2.459
Sh3bgrl2	212531	SH3 domain binding glutamic acid-rich protein like 2	0.99	2.459
8430427H17Rik	329540	RIKEN cDNA 8430427H17 gene	-0.35	2.433
Prg4	96875	proteoglycan 4 (megakaryocyte stimulating factor, articular superficial zone protein)	-0.21	2.433
Melk	17279	maternal embryonic leucine zipper kinase	-0.08	2.433
Tnfrsf4	22163	tumor necrosis factor receptor superfamily, member 4	-1.47	2.406
Mbnl3	171170	muscleblind-like 3 (Drosophila)	-0.68	2.406
Slc22a2	20518	solute carrier family 22 (organic cation transporter), member 2	-0.46	2.379
Map3k8	26410	mitogen activated protein kinase kinase kinase 8	-0.12	2.379
1110032F04Rik	68725	RIKEN cDNA 1110032F04 gene	-0.83	2.350
Arhgap20	244867	Rho GTPase activating protein 20	-0.7	2.350
Rragd	52187	Ras-related GTP binding D	-0.16	2.322
Dst	13518	dystonin	-0.44	2.263
Usp27x	54651	ubiquitin specific peptidase 27, X chromosome	0.03	2.263
Serpina3g	20715	serine (or cysteine) peptidase inhibitor, clade A, member 3G	-0.86	2.233
Cpe	12876	carboxypeptidase E	0.24	2.202
6430570G24	327989	Hypothetical protein 6430570G24	-0.54	2.170
Folr4	64931	folate receptor 4 (delta)	-0.22	2.170
Gata1	14460	GATA binding protein 1	0.14	2.170
5830456J23Rik	76105	RIKEN cDNA 5830456J23 gene	-0.59	2.104
Klrg1	50928	killer cell lectin-like receptor subfamily G, member 1	-0.19	2.070
Pmaip1	58801	phorbol-12-myristate-13-acetate-induced protein 1	-0.44	2.036
Ptpn13	19249	protein tyrosine phosphatase, non-receptor type 13	-0.07	2.036
Gbp4	55932	guanylate nucleotide binding protein 4	0.26	2.036
Rabgap1l	29809	RAB GTPase activating protein 1-like	-0.69	2.000
Spry1	24063	sprouty homolog 1 (Drosophila)	-0.59	2.000
Gnaq	14682	guanine nucleotide binding protein, alpha q polypeptide	-0.2	2.000
H2-Ob	15002	histocompatibility 2, O region beta locus	-0.12	2.000
4921524J06Rik	77057	RIKEN cDNA 4921524J06 gene	0	2.000
Vav2	22325	Vav2 oncogene	-0.57	1.963
Cd200	17470	Cd200 antigen	-0.98	1.926
Trim59	66949	Tripartite motif-containing 59	-0.39	1.926
Frmd4b	232288	FERM domain containing 4B	-0.06	1.926
Niban	63913	niban protein	-0.52	1.888
Cd86	12524	CD86 antigen	-0.3	1.888
Cd83	12522	CD83 antigen	-1.81	1.848
Eno3	13808	enolase 3, beta muscle	-0.89	1.807
Bhlhb2	20893	basic helix-loop-helix domain containing, class B2	-0.76	1.807
Nt5e	23959	5' nucleotidase, ecto	-0.87	1.766
Ndg2	103172	Nur77 downstream gene 2	-0.82	1.766
Plcl1	227120	Phospholipase C-like 1	-0.31	1.766
Slc35d1	242585	Solute carrier family 35 (UDP-glucuronic acid/UDP-N-acetylgalactosamine dual transporter), member D1	-0.29	1.766
Gpr34	23890	G protein-coupled receptor 34	0.53	1.766
Rcn1	19672	reticulocalbin 1	0.67	1.766
Hivep3	16656	human immunodeficiency virus type I enhancer binding protein 3	-0.77	1.722
Kif13a	16553	kinesin family member 13A	-0.48	1.722
Gfi1	14581	growth factor independent 1	-0.46	1.722
Adamts6	108154	a disintegrin-like and metallopeptidase (reprolysin type) with thrombospondin type 1 motif, 6	-0.4	1.722
Il1rl1	17082	interleukin 1 receptor-like 1	0.07	1.722
Swap70	20947	SWA-70 protein	-0.89	1.678
Capn3	12335	calpain 3	-0.59	1.678
Maf	17132	avian musculoaponeurotic fibrosarcoma (v-maf) AS42 oncogene homolog	-0.43	1.678
Lrig1	16206	leucine-rich repeats and immunoglobulin-like domains 1	-0.35	1.678
Gbp1	14468	guanylate nucleotide binding protein 1	-0.06	1.678
Plscr1	22038	phospholipid scramblase 1	-0.4	1.632
Adam19	11492	a disintegrin and metallopeptidase domain 19 (meltrin beta)	0.01	1.632
Phtf2	68770	Putative homeodomain transcription factor 2	-0.59	1.585
2510009E07Rik	72190	RIKEN cDNA 2510009E07 gene	-0.3	1.585
Cttn	13043	cortactin	0.15	1.585
Stx11	74732	syntaxin 11	-0.83	1.536
Cd81	12520	CD 81 antigen	-0.73	1.536

Table S4A

Igf1r	16001	Insulin-like growth factor I receptor	-0.25	1.536
Coro2a	107684	coronin, actin binding protein 2A	-0.19	1.536
Ecm1	13601	extracellular matrix protein 1	-0.13	1.536
Cish	12700	cytokine inducible SH2-containing protein	-0.07	1.536
Pip5k1a	18719	phosphatidylinositol-4-phosphate 5-kinase, type 1 alpha	0.02	1.536
Gsto1	14873	glutathione S-transferase omega 1	-1.22	1.485
Ccr6	12458	chemokine (C-C motif) receptor 6	-0.78	1.485
1200007D18Rik	67458	RIKEN cDNA 1200007D18 gene	-0.49	1.485
Ahr	11622	aryl-hydrocarbon receptor	-0.38	1.485
Slc2a3	20527	solute carrier family 2 (facilitated glucose transporter), member 3	-0.12	1.485
Cd38	12494	CD38 antigen	-0.1	1.485
Tktl1	83553	transketolase-like 1	-0.06	1.485
Sh3gl3	20408	SH3-domain GRB2-like 3	-0.9	1.433
Cyfip1	20430	cytoplasmic FMR1 interacting protein 1	-0.88	1.433
Cerk	223753	ceramide kinase	-0.39	1.433
Serpinb6b	20708	serine (or cysteine) peptidase inhibitor, clade B, member 6b	-0.16	1.433
Traf1	22029	Tnf receptor-associated factor 1	0.03	1.433
Camk2b	12323	Calcium/calmodulin-dependent protein kinase II, beta	0.04	1.433
Hipk2	15258	Homeodomain interacting protein kinase 2	0.05	1.433
Tnfrsf9	21942	tumor necrosis factor receptor superfamily, member 9	-1.21	1.379
Tnfrsf1b	21938	tumor necrosis factor receptor superfamily, member 1b	-0.66	1.379
Anxa4	11746	annexin A4	-0.48	1.379
Phlda1	21664	pleckstrin homology-like domain, family A, member 1	-0.35	1.379
E030024N20Rik	319427	RIKEN cDNA E030024N20 gene	-0.09	1.379
Slc22a5	20520	solute carrier family 22 (organic cation transporter), member 5	0.04	1.379
C80638	97086	expressed sequence C80638	-1.36	1.322
Gabarapl1	57436	gamma-aminobutyric acid (GABA(A)) receptor-associated protein-like 1	-0.24	1.322
Xdh	22436	xanthine dehydrogenase	-0.1	1.322
Ppm1l	242083	protein phosphatase 1 (formerly 2C)-like	-0.08	1.322
Tnfsf11	21943	tumor necrosis factor (ligand) superfamily, member 11	-1.19	1.263
Penk1	18619	preproenkephalin 1	-0.9	1.263
Zfp52	22710	zinc finger protein 52	-0.67	1.263
Eno2	13807	enolase 2, gamma neuronal	-0.56	1.263
Igf2r	16004	insulin-like growth factor 2 receptor	-0.45	1.263
Eea1	216238	early endosome antigen 1	-0.43	1.263
Rhoq	104215	ras homolog gene family, member Q	-0.42	1.263
Ggta1	14594	glycoprotein galactosyltransferase alpha 1, 3	-0.39	1.263
Arhgef12	69632	Rho guanine nucleotide exchange factor (GEF) 12	-0.28	1.263
1810054D07Rik	69863	RIKEN cDNA 1810054D07 gene	-0.2	1.263
C230081A13Rik	244895	RIKEN cDNA C230081A13 gene	0.04	1.263
Ga17	98221	Dendritic cell protein GA17	0.11	1.263
6330509M05Rik	102913	RIKEN cDNA 6330509M05 gene	-0.51	1.202
Scamp1	107767	secretory carrier membrane protein 1	-0.37	1.202
Eef2k	13631	eukaryotic elongation factor-2 kinase	-0.34	1.202
Zfp467	68910	zinc finger protein 467	-0.27	1.202
Ddc	13195	dopa decarboxylase	-0.26	1.202
2510042P03Rik	72421	RIKEN cDNA 2510042P03 gene	-0.11	1.202
Neb	17996	nebulin	0.04	1.202
4631427C17Rik	74340	RIKEN cDNA 4631427C17 gene	0.11	1.202
Nrn1	68404	neuritin 1	-1.4	1.138
Samsn1	67742	SAM domain, SH3 domain and nuclear localization signals, 1	-1.1	1.138
Trpm1	17364	transient receptor potential cation channel, subfamily M, member 1	-0.69	1.138
Ndrg1	17988	N-myc downstream regulated gene 1	-0.61	1.138
Csda	56449	cold shock domain protein A	-0.4	1.138
Myo1e	71602	myosin IE	-0.38	1.138
Entpd1	12495	ectonucleoside triphosphate diphosphohydrolase 1	-0.36	1.138
Rnase4	58809	ribonuclease, RNase A family 4	-0.22	1.138
C030046G05	327885	hypothetical protein C030046G05	-1.03	1.070
Inpp5f	101490	inositol polyphosphate-5-phosphatase F	-0.78	1.070
Osbpl3	71720	oxysterol binding protein-like 3	-0.66	1.070
A530052I06Rik	99953	RIKEN cDNA A530052I06 gene	-0.62	1.070
Kcnk6	52150	potassium inwardly-rectifying channel, subfamily K, member 6	-0.45	1.070
Gphn	268566	gephyrin	-0.44	1.070
Ndrl	17990	N-myc downstream regulated-like	-0.31	1.070
4930566A11Rik	75871	RIKEN cDNA 4930566A11 gene	-0.28	1.070
Galm	319625	galactose mutarotase	-0.21	1.070
Slamf1	27218	signaling lymphocytic activation molecule family member 1	-0.18	1.070
Ccrl2	54199	chemokine (C-C motif) receptor-like 2	-0.13	1.070
Gadd45g	23882	growth arrest and DNA-damage-inducible 45 gamma	-0.08	1.070
Rab6ip1	19347	Rab6 interacting protein 1	0.01	1.070
4833442J19Rik	320204	RIKEN cDNA 4833442J19 gene	0.2	1.070
Slc14a1	108052	solute carrier family 14 (urea transporter), member 1	0.21	1.070
1300007C21Rik	67527	RIKEN cDNA 1300007C21 gene	-1.32	1.000
Ptprs	19280	protein tyrosine phosphatase, receptor type, S	-0.95	1.000
Alcam	11658	activated leukocyte cell adhesion molecule	-0.92	1.000
Nr4a2	18227	Nuclear receptor subfamily 4, group A, member 2	-0.69	1.000
Syt11	229521	synaptotagmin XI	-0.51	1.000
Snag1	170625	sorting nexin associated golgi protein 1	-0.31	1.000
2510005D08Rik	68043	RIKEN cDNA 2510005D08 gene	-0.22	1.000
Matn2	17181	matrilin 2	-0.18	1.000
AI875142	103885	expressed sequence AI875142	-0.17	1.000
Vps54	245944	vacuolar protein sorting 54 (yeast)	-0.16	1.000
Cdk6	12571	cyclin-dependent kinase 6	-0.1	1.000
Smyd2	226830	SET and MYND domain containing 2	-0.02	1.000
Samhd1	56045	SAM domain and HD domain, 1	0.29	1.000

Table S4A

Tgm2	21817	transglutaminase 2, C polypeptide	0.47	1.000
Sh3bgrl	56726	SH3-binding domain glutamic acid-rich protein like	-0.63	0.926
Tgfbr1	21812	transforming growth factor, beta receptor I	-0.57	0.926
Myo1c	17913	myosin IC	-0.5	0.926
Socs5	56468	suppressor of cytokine signaling 5	-0.46	0.926
Evi5	14020	ecotropic viral integration site 5	-0.44	0.926
Gem	14579	GTP binding protein (gene overexpressed in skeletal muscle)	-0.31	0.926
D630039A03Rik	242484	RIKEN cDNA D630039A03 gene	-0.27	0.926
Zfpn1a3	22780	zinc finger protein, subfamily 1A, 3 (Aiolos)	-0.22	0.926
Bmpr2	12168	bone morphogenic protein receptor, type II (serine/threonine kinase)	-0.19	0.926
Plcb4	18798	phospholipase C, beta 4	-0.03	0.926
Lta	16992	lymphotoxin A	0.06	0.926
2810402A17Rik	67062	RIKEN cDNA 2810402A17 gene	0.12	0.926
Egr2	13654	early growth response 2	-1.45	0.848
Ephx1	13849	epoxide hydrolase 1, microsomal	-0.96	0.848
Tank	21353	TRAF family member-associated Nf-kappa B activator	-0.7	0.848
Plp2	18824	proteolipid protein 2	-0.49	0.848
Tnfrsf18	21936	tumor necrosis factor receptor superfamily, member 18	-0.42	0.848
Capg	12332	capping protein (actin filament), gelsolin-like	-0.37	0.848
Slc12a2	20496	solute carrier family 12, member 2	-0.32	0.848
D18Ertd653e	52662	DNA segment, Chr 18, ERATO Doi 653, expressed	-0.24	0.848
Prkca	18750	Protein kinase C, alpha	-0.18	0.848
E2f3	13557	E2F transcription factor 3	-0.16	0.848
Laptm4b	114128	lysosomal-associated protein transmembrane 4B	-0.12	0.848
Fabp5	16592	fatty acid binding protein 5, epidermal	-0.11	0.848
Nipa2	93790	non imprinted in Prader-Willi/Angelman syndrome 2 homolog (human)	-0.11	0.848
Mgat5	107895	mannoside acetylglucosaminyltransferase 5	-0.11	0.848
Casp7	12369	caspase 7	-0.1	0.848
Sla	20491	Src-like adaptor	-0.06	0.848
Bid	12122	BH3 interacting domain death agonist	-0.06	0.848
Abcb1a	18671	ATP-binding cassette, sub-family B (MDR/TAP), member 1A	-0.01	0.848
Lpxn	107321	leupaxin	0.02	0.848
Slc4a7	218756	solute carrier family 4, sodium bicarbonate cotransporter, member 7	0.03	0.848
2010111I01Rik	72061	RIKEN cDNA 2010111I01 gene	0.04	0.848
BC053440	234344	cDNA sequence BC053440	0.06	0.848
Pros1	19128	protein S (alpha)	0.07	0.848
Sec24a	77371	SEC24 related gene family, member A (S. cerevisiae)	0.39	0.848
Pdcd1lg2	58205	programmed cell death 1 ligand 2	-0.54	0.766
Lxn	17035	latexin	-0.5	0.766
Ptger4	19219	prostaglandin E receptor 4 (subtype EP4)	-0.48	0.766
Ebi3	50498	Epstein-Barr virus induced gene 3	-0.45	0.766
A530088I07Rik	212167	RIKEN cDNA A530088I07 gene	-0.43	0.766
5830474E16Rik	76094	RIKEN cDNA 5830474E16 gene	-0.36	0.766
D10Ucla1	28193	DNA segment, Chr 10, University of California at Los Angeles 1	-0.28	0.766
Nfil3	18030	nuclear factor, interleukin 3, regulated	-0.24	0.766
Gprasp2	245607	G protein-coupled receptor associated sorting protein 2	-0.23	0.766
Icam1	15894	intercellular adhesion molecule	-0.2	0.766
Tnfrsf25	85030	tumor necrosis factor receptor superfamily, member 25	-0.2	0.766
Atp6v0a1	11975	ATPase, H+ transporting, lysosomal V0 subunit A1	-0.19	0.766
Rora	19883	RAR-related orphan receptor alpha	-0.18	0.766
2810051F02Rik	72704	RIKEN cDNA 2810051F02 gene	-0.12	0.766
Bcl2l1	12048	Bcl2-like 1	-0.11	0.766
Ibrdc3	75234	IBR domain containing 3	-0.06	0.766
AI480653	268880	expressed sequence AI480653	-0.06	0.766
Pscd3	19159	pleckstrin homology, Sec7 and coiled-coil domains 3	0.08	0.766
P2ry10	78826	purinergic receptor P2Y, G-protein coupled 10	0.29	0.766
Gzmb	14939	granzyme B	0.41	0.766
Cst7	13011	cystatin F (leukocystatin)	-0.78	0.678
4732460K03Rik	58894	RIKEN cDNA 4732460K03 gene	-0.77	0.678
Birc1e	17951	baculoviral IAP repeat-containing 1e	-0.43	0.678
6330403K07Rik	103712	RIKEN cDNA 6330403K07 gene	-0.41	0.678
Sdc4	20971	syndecan 4	-0.41	0.678
Crsp9	66213	cofactor required for Sp1 transcriptional activation, subunit 9	-0.38	0.678
Atpif1	11983	ATPase inhibitory factor 1	-0.36	0.678
2610027H17Rik	71811	RIKEN cDNA 2610027H17 gene	-0.35	0.678
Sypl	19027	synaptophysin-like protein	-0.23	0.678
Homer1	26556	homer homolog 1 (Drosophila)	-0.21	0.678
Unc119	22248	unc-119 homolog (C. elegans)	-0.18	0.678
Arrdc4	66412	arrestin domain containing 4	-0.15	0.678
Pafah1b3	18476	platelet-activating factor acetylhydrolase, isoform 1b, alpha1 subunit	-0.15	0.678
3110045G13Rik	73182	RIKEN cDNA 3110045G13 gene	-0.07	0.678
Ahnak	11621	AHNAK nucleoprotein (desmoyokin)	0.01	0.678
Csrp2	13008	cysteine and glycine-rich protein 2	0.09	0.678
Car12	76459	carbonic anyhydrase 12	0.09	0.678
Tlr7	170743	toll-like receptor 7	0.15	0.678
P4ha1	18451	procollagen-proline, 2-oxoglutarate 4-dioxygenase (proline 4-hydroxylase), alpha 1 polypeptide	0.21	0.678
2810474O19Rik	67246	RIKEN cDNA 2810474O19 gene	0.36	0.678
Irf4	16364	interferon regulatory factor 4	-1.02	0.585
Twsg1	65960	twisted gastrulation homolog 1 (Drosophila)	-0.86	0.585
E130308A19Rik	230259	RIKEN cDNA E130308A19 gene	-0.71	0.585
1700108L22Rik	214048	RIKEN cDNA 1700108L22 gene	-0.44	0.585
Mapk12	29857	mitogen-activated protein kinase 12	-0.38	0.585
Dapp1	26377	dual adaptor for phosphotyrosine and 3-phosphoinositides 1	-0.17	0.585
Hdgfrp3	29877	hepatoma-derived growth factor, related protein 3	-0.01	0.585
Hist2h2be	319190	histone 2, H2be	-0.25	-0.515

Table S4A

Bcl9	77578	B-cell CLL/lymphoma 9	-0.11	-0.515
Sestd1	228071	SEC14 and spectrin domains 1	-0.07	-0.515
1300007F04Rik	67477	RIKEN cDNA 1300007F04 gene	0.01	-0.515
Entpd5	12499	ectonucleoside triphosphate diphosphohydrolase 5	0.39	-0.515
Klhdc2	69554	kelch domain containing 2	-0.55	-0.737
Igsf6	80719	immunoglobulin superfamily, member 6	-0.44	-0.737
Pde7a	NA	phosphodiesterase 7A	-0.12	-0.737
Pim2	18715	proviral integration site 2	-0.06	-0.737
Bach2	12014	BTB and CNC homology 2	0.01	-0.737
Elovl7	74559	ELOVL family member 7, elongation of long chain fatty acids (yeast)	0.07	-0.737
Ranbp10	74334	RAN binding protein 10	0.09	-0.737
Tle4	21888	transducin-like enhancer of split 4, homolog of Drosophila E(spl)	0.1	-0.737
Gsn	227753	gelsolin	0.12	-0.737
Spnb2	20742	spectrin beta 2	0.12	-0.737
Nlk	18099	nemo like kinase	0.13	-0.737
B930041F14Rik	230991	RIKEN cDNA B930041F14 gene	0.2	-0.737
Add3	27360	adducin 3 (gamma)	0.21	-0.737
Rassf3	192678	Ras association (RalGDS/AF-6) domain family 3	0.22	-0.737
Itk	16428	IL2-inducible T-cell kinase	0.24	-0.737
Gpd2	14571	glycerol phosphate dehydrogenase 2, mitochondrial	0.35	-0.737
C230085N15Rik	320556	RIKEN cDNA C230085N15 gene	0.35	-0.737
Tmie	20776	transmembrane inner ear	0.38	-0.737
Rsn	56430	restin (Reed-Steinberg cell-expressed intermediate filament-associated protein)	0.39	-0.737
Lef1	16842	lymphoid enhancer binding factor 1	0.47	-0.737
C230093N12Rik	98952	RIKEN cDNA C230093N12 gene	0.53	-0.737
C030002B11Rik	78540	RIKEN cDNA C030002B11 gene	0.61	-0.737
Pdk1	228026	pyruvate dehydrogenase kinase, isoenzyme 1	0.67	-0.737
Dntt	21673	deoxynucleotidyltransferase, terminal	0.83	-0.737
5830431A10Rik	76024	RIKEN cDNA 5830431A10 gene	0.88	-0.737
1500005K14Rik	76566	RIKEN cDNA 1500005K14 gene	1.14	-0.737
4930431H11Rik	73785	RIKEN cDNA 4930431H11 gene	-0.43	-1.000
Ebi2	321019	Epstein-Barr virus induced gene 2	-0.39	-1.000
Gpr171	229323	G protein-coupled receptor 171	-0.32	-1.000
A430107P09Rik	320898	RIKEN cDNA A430107P09 gene	-0.14	-1.000
2210010L05Rik	98682	RIKEN cDNA 2210010L05 gene	-0.02	-1.000
Evi2b	216984	ecotropic viral integration site 2b	0.05	-1.000
AB124611	382062	cDNA sequence AB124611	0.05	-1.000
Ggtla1	23887	gamma-glutamyltransferase-like activity 1	0.06	-1.000
Tgfbr2	21813	transforming growth factor, beta receptor II	0.1	-1.000
Ust	338362	Uronyl-2-sulfotransferase	0.12	-1.000
Daam1	208846	dishevelled associated activator of morphogenesis 1	0.21	-1.000
Gmfg	63986	glia maturation factor, gamma	0.21	-1.000
Lyst	17101	lysosomal trafficking regulator	0.22	-1.000
Sema4a	20351	sema domain, Ig domain (Ig), transmembrane domain (TM) and short cytoplasmic domain, (semaphorin) 4A	0.22	-1.000
1110038D17Rik	68778	RIKEN cDNA 1110038D17 gene	0.22	-1.000
Slc20a1	20515	solute carrier family 20, member 1	0.22	-1.000
Ugcg	22234	UDP-glucose ceramide glucosyltransferase	0.24	-1.000
C030044B11Rik	68128	RIKEN cDNA C030044B11 gene	0.25	-1.000
Rtn4rl1	237847	reticulon 4 receptor-like 1	0.25	-1.000
Rasgrp2	19395	RAS, guanyl releasing protein 2	0.26	-1.000
Ggt1	14598	gamma-glutamyltransferase 1	0.28	-1.000
1190002H23Rik	66214	RIKEN cDNA 1190002H23 gene	0.31	-1.000
D730035F11Rik	320010	RIKEN cDNA D730035F11 gene	0.33	-1.000
Scml4	268297	sex comb on midleg-like 4 (Drosophila)	0.34	-1.000
Slc12a7	20499	solute carrier family 12, member 7	0.39	-1.000
E430014B02Rik	320908	RIKEN cDNA E430014B02 gene	0.46	-1.000
Satb1	20230	Special AT-rich sequence binding protein 1	0.59	-1.000
E430004N04Rik	210757	RIKEN cDNA E430004N04 gene	0.72	-1.000
Atp1b1	11931	ATPase, Na+/K+ transporting, beta 1 polypeptide	0.98	-1.000
F2rl1	14063	coagulation factor II (thrombin) receptor-like 1	-0.54	-1.322
Snn	20621	stannin	-0.5	-1.322
Ppic	19038	peptidylprolyl isomerase C	-0.21	-1.322
Klhdc1	271005	kelch domain containing 1	-0.06	-1.322
Gpr97	54672	G protein-coupled receptor 97	-0.05	-1.322
Ccl5	20304	chemokine (C-C motif) ligand 5	-0.04	-1.322
Phxr4	18689	per-hexamer repeat gene 4	-0.03	-1.322
Tmem23	208449	transmembrane protein 23	0.03	-1.322
Cyp2s1	74134	cytochrome P450, family 2, subfamily s, polypeptide 1	0.09	-1.322
Ibtk	108837	inhibitor of Bruton agammaglobulinemia tyrosine kinase	0.14	-1.322
Adh1	11522	alcohol dehydrogenase 1 (class I)	0.18	-1.322
Tcf7	21414	transcription factor 7, T-cell specific	0.38	-1.322
Chd7	320790	chromodomain helicase DNA binding protein 7	0.43	-1.322
Emb	13723	embigin	0.53	-1.322
A130092J06Rik	241303	RIKEN cDNA A130092J06 gene	0.83	-1.322
Pole2	18974	polymerase (DNA directed), epsilon 2 (p59 subunit)	-0.56	-1.737
Itgb3	16416	integrin beta 3	-0.16	-1.737
Lair1	52855	leukocyte-associated Ig-like receptor 1	-0.05	-1.737
Btbd14a	67991	BTB (POZ) domain containing 14A	0.01	-1.737
Acpl2	235534	acid phosphatase-like 2	0.02	-1.737
Wwox	80707	WW domain-containing oxidoreductase	0.1	-1.737
Myo10	17909	Myosin X	0.1	-1.737
Sema4f	20355	sema domain, immunoglobulin domain (Ig), TM domain, and short cytoplasmic domain	0.11	-1.737
Enc1	13803	ectodermal-neural cortex 1	0.15	-1.737
Ntrk3	18213	Neurotrophic tyrosine kinase, receptor, type 3	0.25	-1.737
2810001G20Rik	66456	RIKEN cDNA 2810001G20 gene	0.27	-1.737

Table S4A

Rgmb	68799	RGM domain family, member B	0.3	-1.737
Tgfbr3	21814	transforming growth factor, beta receptor III	0.31	-1.737
Acpp	56318	acid phosphatase, prostate	0.42	-1.737
Cnga1	12788	cyclic nucleotide gated channel alpha 1	0.42	-1.737
2010004M13Rik	76511	RIKEN cDNA 2010004M13 gene	0.46	-1.737
Klhl5	71778	kelch-like 5 (Drosophila)	0.51	-1.737
Igfbp4	16010	insulin-like growth factor binding protein 4	0.93	-1.737
Pou2af1	18985	POU domain, class 2, associating factor 1	-0.43	-2.322
Hs3st3b1	54710	heparan sulfate (glucosamine) 3-O-sulfotransferase 3B1	-0.04	-2.322
Epas1	13819	Endothelial PAS domain protein 1	0.03	-2.322
Dip3b	216190	Dip3 beta	0.11	-2.322
Sgk	20393	serum/glucocorticoid regulated kinase	0.3	-2.322
2610019F03Rik	72148	RIKEN cDNA 2610019F03 gene	0.67	-2.322
Slc16a5	217316	solute carrier family 16 (monocarboxylic acid transporters), member 5	0.95	-2.322
2310010M24Rik	71897	RIKEN cDNA 2310010M24 gene	-0.1	-3.322
Klrd1	16643	killer cell lectin-like receptor, subfamily D, member 1	-0.06	-3.322
AI646023	192734	expressed sequence AI646023	-0.02	-3.322
Il1rl2	107527	interleukin 1 receptor-like 2	0.07	-3.322
Atp8b4	241633	ATPase, class I, type 8B, member 4	0.1	-3.322
App	11820	amyloid beta (A4) precursor protein	0.14	-3.322
Pde3b	18576	phosphodiesterase 3B, cGMP-inhibited	0.24	-3.322
Rapgef4	56508	Rap guanine nucleotide exchange factor (GEF) 4	0.37	-3.322
Pdlim4	30794	PDZ and LIM domain 4	0.81	-3.322
2310032F03Rik	76747	RIKEN cDNA 2310032F03 gene	0.96	-3.322

Table S4 B

Symbol	Gene ID	Description	Treg Lck fold change (log_2)	Treg/Tconv signature (log_2)
Dusp4	319520	dual specificity phosphatase 4	-1.3	3.621
4631408O11Rik	66693	RIKEN cDNA 4631408O11 gene	-0.79	3.392
Ctla4	12477	cytotoxic T-lymphocyte-associated protein 4	-0.62	3.202
Zfpn1a2	22779	zinc finger protein, subfamily 1A, 2 (Helios)	-0.79	2.848
Nrp1	18186	neuropilin 1	-0.83	2.807
Tiam1	21844	T-cell lymphoma invasion and metastasis 1	-1.1	2.766
Hmgn3	94353	high mobility group nucleosomal binding domain 3	-1.05	2.678
Itgae	16407	integrin, alpha E, epithelial-associated	-0.9	2.609
Ptger2	19217	prostaglandin E receptor 2 (subtype EP2)	-1.06	2.511
Sh3bgrl2	212531	*SH3 domain binding glutamic acid-rich protein like 2*	0.99	2.459
Tnfrsf4	22163	tumor necrosis factor receptor superfamily, member 4	-1.47	2.406
Mbnl3	171170	muscleblind-like 3 (Drosophila)	-0.68	2.406
1110032F04Rik	68725	RIKEN cDNA 1110032F04 gene	-0.83	2.350
Arhgap20	244867	Rho GTPase activating protein 20	-0.7	2.350
Serpina3g	20715	serine (or cysteine) peptidase inhibitor, clade A, member 3G	-0.86	2.233
5830456J23Rik	76105	RIKEN cDNA 5830456J23 gene	-0.59	2.104
Rabgap1l	29809	RAB GTPase activating protein 1-like	-0.69	2.000
Spry1	24063	sprouty homolog 1 (Drosophila)	-0.59	2.000
Cd200	17470	Cd200 antigen	-0.98	1.926
Cd83	12522	CD83 antigen	-1.81	1.848
Eno3	13808	enolase 3, beta muscle	-0.89	1.807
Bhlhb2	20893	basic helix-loop-helix domain containing, class B2	-0.76	1.807
Nt5e	23959	5' nucleotidase, ecto	-0.87	1.766
Ndg2	103172	Nur77 downstream gene 2	-0.82	1.766
Rcn1	19672	*reticulocalbin 1*	0.67	1.766
Hivep3	16656	human immunodeficiency virus type I enhancer binding protein 3	-0.77	1.722
Swap70	20947	SWA-70 protein	-0.89	1.678
Capn3	12335	calpain 3	-0.59	1.678
Phtf2	68770	Putative homeodomain transcription factor 2	-0.59	1.585
Stx11	74732	syntaxin 11	-0.83	1.536
Cd81	12520	CD 81 antigen	-0.73	1.536
Gsto1	14873	glutathione S-transferase omega 1	-1.22	1.485
Ccr6	12458	chemokine (C-C motif) receptor 6	-0.78	1.485
Sh3gl3	20408	SH3-domain GRB2-like 3	-0.9	1.433
Cyfip1	20430	cytoplasmic FMR1 interacting protein 1	-0.88	1.433
Tnfrsf9	21942	tumor necrosis factor receptor superfamily, member 9	-1.21	1.379
Tnfrsf1b	21938	tumor necrosis factor receptor superfamily, member 1b	-0.66	1.379
C80638	97086	expressed sequence C80638	-1.36	1.322
Tnfsf11	21943	tumor necrosis factor (ligand) superfamily, member 11	-1.19	1.263
Penk1	18619	preproenkephalin 1	-0.9	1.263
Zfp52	22710	zinc finger protein 52	-0.67	1.263
Nrn1	68404	neuritin 1	-1.4	1.138
Samsn1	67742	SAM domain, SH3 domain and nuclear localization signals, 1	-1.1	1.138
Trpm1	17364	transient receptor potential cation channel, subfamily M, member 1	-0.69	1.138
Ndrg1	17988	N-myc downstream regulated gene 1	-0.61	1.138
C030046G05	327885	hypothetical protein C030046G05	-1.03	1.070
Inpp5f	101490	inositol polyphosphate-5-phosphatase F	-0.78	1.070
Osbpl3	71720	oxysterol binding protein-like 3	-0.66	1.070
A530052I06Rik	99953	RIKEN cDNA A530052I06 gene	-0.62	1.070
Ptprs	19280	protein tyrosine phosphatase, receptor type, S	-0.95	1.000
Alcam	11658	activated leukocyte cell adhesion molecule	-0.92	1.000
Nr4a2	18227	Nuclear receptor subfamily 4, group A, member 2	-0.69	1.000
Sh3bgrl	56726	SH3-binding domain glutamic acid-rich protein like	-0.63	0.926
Egr2	13654	early growth response 2	-1.45	0.848
Ephx1	13849	epoxide hydrolase 1, microsomal	-0.96	0.848
Tank	21353	TRAF family member-associated Nf-kappa B activator	-0.7	0.848
Cst7	13011	cystatin F (leukocystatin)	-0.78	0.678
4732460K03Rik	58894	RIKEN cDNA 4732460K03 gene	-0.77	0.678
Irf4	16364	interferon regulatory factor 4	-1.02	0.585
Twsg1	65960	twisted gastrulation homolog 1 (Drosophila)	-0.86	0.585
E130308A19Rik	230259	RIKEN cDNA E130308A19 gene	-0.71	0.585
C030002B11Rik	78540	RIKEN cDNA C030002B11 gene	0.61	-0.737
Pdk1	228026	pyruvate dehydrogenase kinase, isoenzyme 1	0.67	-0.737
Dntt	21673	deoxynucleotidyltransferase, terminal	0.83	-0.737
5830431A10Rik	76024	RIKEN cDNA 5830431A10 gene	0.88	-0.737
1500005K14Rik	76566	RIKEN cDNA 1500005K14 gene	1.14	-0.737
Satb1	20230	Special AT-rich sequence binding protein 1	0.59	-1.000
E430004N04Rik	210757	RIKEN cDNA E430004N04 gene	0.72	-1.000
Atp1b1	11931	ATPase, Na+/K+ transporting, beta 1 polypeptide	0.98	-1.000
A130092J06Rik	241303	RIKEN cDNA A130092J06 gene	0.83	-1.322
Igfbp4	16010	insulin-like growth factor binding protein 4	0.93	-1.737
2610019F03Rik	72148	RIKEN cDNA 2610019F03 gene	0.67	-2.322
Slc16a5	217316	solute carrier family 16 (monocarboxylic acid transporters), member 5	0.95	-2.322
Pdlim4	30794	PDZ and LIM domain 4	0.81	-3.322
2310032F03Rik	76747	RIKEN cDNA 2310032F03 gene	0.96	-3.322
